# High-throughput analysis of candidate imprinted genes and allele-specific gene expression in the human term placenta

**DOI:** 10.1186/1471-2156-11-25

**Published:** 2010-04-19

**Authors:** Caroline Daelemans, Matthew E Ritchie, Guillaume Smits, Sayeda Abu-Amero, Ian M Sudbery, Matthew S Forrest, Susana Campino, Taane G Clark, Philip Stanier, Dominic Kwiatkowski, Panos Deloukas, Emmanouil T Dermitzakis, Simon Tavaré, Gudrun E Moore, Ian Dunham

**Affiliations:** 1 Wellcome Trust Sanger Institute, Hinxton, Cambridge, CB10 1 SA, UK; 2 Molecular and Clinical Genetics Unit, Institute of Child Health, London, WC1 1EH, UK; 3 Department of Obstetrics and Gynecology, Institute for Women's Health, University College London, London, WC1E 6HX, UK; 4 Department of Oncology, University of Cambridge, CRUK Cambridge Research Institute, Li Ka Shing Centre, Robinson Way, Cambridge, CB2 0RE, UK; 5 Bioinformatics Division, The Walter and Eliza Hall Institute of Medical Research, Parkville, Victoria 3052, Australia; 6 Department of Genetic Medicine and Development, University of Geneva Medical School, Geneva, Switzerland; 7 European Bioinformatics Institute, Hinxton, Cambridge, CB10 1SD, UK

## Abstract

**Background:**

Imprinted genes show expression from one parental allele only and are important for development and behaviour. This extreme mode of allelic imbalance has been described for approximately 56 human genes. Imprinting status is often disrupted in cancer and dysmorphic syndromes. More subtle variation of gene expression, that is not parent-of-origin specific, termed 'allele-specific gene expression' (ASE) is more common and may give rise to milder phenotypic differences. Using two allele-specific high-throughput technologies alongside bioinformatics predictions, normal term human placenta was screened to find new imprinted genes and to ascertain the extent of ASE in this tissue.

**Results:**

Twenty-three family trios of placental cDNA, placental genomic DNA (gDNA) and gDNA from both parents were tested for 130 candidate genes with the Sequenom MassArray system. Six genes were found differentially expressed but none imprinted. The Illumina ASE BeadArray platform was then used to test 1536 SNPs in 932 genes. The array was enriched for the human orthologues of 124 mouse candidate genes from bioinformatics predictions and 10 human candidate imprinted genes from EST database mining. After quality control pruning, a total of 261 informative SNPs (214 genes) remained for analysis. Imprinting with maternal expression was demonstrated for the lymphocyte imprinted gene *ZNF331 *in human placenta. Two potential differentially methylated regions (DMRs) were found in the vicinity of *ZNF331*. None of the bioinformatically predicted candidates tested showed imprinting except for a skewed allelic expression in a parent-specific manner observed for *PHACTR2*, a neighbour of the imprinted *PLAGL1 *gene. ASE was detected for two or more individuals in 39 candidate genes (18%).

**Conclusions:**

Both Sequenom and Illumina assays were sensitive enough to study imprinting and strong allelic bias. Previous bioinformatics approaches were not predictive of new imprinted genes in the human term placenta. *ZNF331 *is imprinted in human term placenta and might be a new ubiquitously imprinted gene, part of a primate-specific locus. Demonstration of partial imprinting of *PHACTR2 *calls for re-evaluation of the allelic pattern of expression for the *PHACTR2-PLAGL1 *locus. ASE was common in human term placenta.

## Background

Although diploid organisms have two copies of each gene, they are not always equally expressed. For some genes, only one allele is active while the other is almost completely silenced. Two different groups of genes fall into this category: genes that exhibit random monoallelic expression, e.g. the odorant receptor genes and genes coding for immunoglobulins [1,2]; and imprinted genes that exhibit monoallelic expression in a parent-of-origin specific manner [3]. Imprinted genes have been shown to be important in fetal and placental development, postnatal growth, behaviour and metabolism [4]. Their regulation has been found to be disturbed in numerous cancers and dysmorphic syndromes [5].

To date, 56 genes have been identified as imprinted in humans and 98 in mice [6]. A catalogue of human imprinted genes is kept and regularly updated at http://igc.otago.ac.nz/home.html[7]. However, since most imprinted have been discovered by direct approaches, the total number of imprinted genes is not yet known. Recently, a bioinformatics approach based on DNA sequence characteristics of known imprinted genes predicted 600 imprinted genes in mice [8]. In the human, statistical models have been developed to identify genes with unequal representation of alternative alleles in the public EST libraries, suggesting a further 55 candidate imprinted genes [9]. Many imprinted genes are expressed in a parent-of-origin specific manner in the placenta, making it a "first choice" tissue in which to screen for new imprinted genes [10].

Imprinted expression is at the extreme end of the autosomal allelic imbalance spectrum. However, more subtle allelic variations around the expected 50:50 ratio of expression have been documented. Yan et al. were the first to report such ASE in human [11]. They studied 13 genes and detected 1.3 to 4.3-fold expression differences between alleles for six of them. Lo et al. studied 1063 genes (using Affymetrix HuSNP array) in seven fetuses, where of the 602 genes that were heterozygous, 326 showed preferential expression of one allele in at least one individual (54%), while 170 (28%) showed more than a four-fold difference between the two alleles [12]. Several oligonucleotide microarrays have been used to study ASE in lymphoblastoid cell lines (LCLs). Pant et al. used a custom made microarray (Perlegen, USA) and found allelic expression differences in at least one individual in 53% of the 1389 genes targeted by heterozygous single nucleotide polymorphisms (SNPs) [13]. More recently, Gimelbrant et al. found monoallelic expression for 7.3% of the genes they tested in clonal lymphoblastoid cells [14]. Strong ASE differences (ASE ratio >4 or <1/4) have been found by Bjornsson et al. in 10% of SNPs in LCLs [15]. Hence, it seems that ASE is frequent, possibly underlying much of human variability [11-15].

We have screened human term placenta for novel imprinted genes and ASE using two technologies that have been shown to be able to quantify allelic expression in a medium and high-throughput manner: the MassArray system (Sequenom, Inc.) [16] and the Illumina ASE Bead Array™[17], respectively.

## Results

### Sequenom

The MassArray system (Sequenom, Inc.) was used to test 143 genes for ASE in at least 23 family-trios. Each trio consisted of placental genomic DNA (gDNA), placental cDNA and both parental gDNAs. We analysed six imprinted control genes, seven biallelically expressed genes, seven orthologues of mouse imprinted genes, 99 orthologues of mouse imprinted candidate genes [8], and 26 human imprinted candidate genes [9] (Additional file 1: Supplemental Table S1). For 123 genes (86%), the cDNA amplification was successful and at least two placentas were heterozygous. A *t*-test (followed by FDR-moderation) was used to verify the null hypothesis that there was no allelic imbalance between the ratios of alleles in gDNA and in cDNA (Table 1 and Methods).

**Table 1:** SNPs and corresponding genes statistically significant when tested for ASE by Sequenom Assay.

Gene	SNP_ID	fdr.p.values	Difference	SR ratio	Mode of ASE
*DLK1*	rs1802710	3.62E-23	88.6	0.89	Imprinting
*PEG3*	rs1860565	2.74E-22	98.1	1.00	Imprinting
*IGF2*	rs680	2.32E-16	94.6	1.04	Imprinting
*PEG10*	rs13073	4.19E-08	98.4	0.84	Imprinting
*PHLDA2*	rs13390	4.19E-08	98.1	1.13	Imprinting
*DISC1*	rs821616	0.022009568	15.3	0.91	Random ASE†
*RASGRF1*	rs11855231	0.022009568	75.7	0.95	Random mono†
*C9orf93*	rs1539172	0.039790941	30.0	0.78	Preferential†
*TF*	rs8649	0.04122505	56.9	0.78	Random ASE†
*ACSS2*	rs4911163	0.04122505	21.9	0.86	Preferential
*KIAA0523*	rs3744725	0.04122505	36.5	0.96	Random ASE†

The *p*-value is adjusted for multiple testing (false discovery rate bound). The average difference of expression between the two alleles in the cDNA of heterozygous individuals is greatest for imprinted genes. SR ratio is the ratio of genotyping success rate of cDNA on gDNA. Mode of ASE summarises the pattern of ASE based on the quantitative allelic expression data. †False positive pattern probably due to a low expression level (see text for details).

Five imprinted control genes exhibited imprinting (no informative sample for rs2066707-*ATP10A*). In the subset of genes with acceptable cDNA genotyping success (arbitrarily set at a ratio between cDNA and gDNA genotyping higher than 75%, see Methods), six candidate genes were significant for allelic imbalance in cDNA (p < 0.05) (Table 1). None of these genes had an allelic expression pattern that was compatible with imprinting. Of these, *RASGRF1 *had the most allelic difference (76%) and it is notable that the mouse orthologue *Rasgrf1 *is imprinted in the brain [18]. Its mode of allelic expression in human term placenta was compatible with random monoallelic expression (no allelic preference; four paternal, one maternal and three biallelic mode of expression; data not shown). We checked the mode of expression of *RASGFR1 *in the human term placenta by Sanger sequencing. Biallelic expression (with sometimes a very slight random bias between alleles) was found in seven informative term placenta samples (data not shown). The average fluorescence level of *RASGFR1 *on the Illumina array was below our cut-off suggesting low expression level (see below). We thus considered *RASGRF1 *random monoallelic ASE to be a false positive.

Using rs4911163 as a readout, *ACSS2 *showed a statistically significant (two-tailed *t*-test, p = 0.0075) preferential mode of ASE (Additional file 2). Using the Genevar database (T-P. Yang and E. Dermitzakis, manuscript in preparation), variable level of expression for *ACSS2 *in relation to rs4911163 genotype was also found in lymphoblastoid cells of HapMap3 individuals (B. Stranger and E. Dermitzakis, manuscript in preparation; [19,20]). *ACSS2 *is a cytosolic enzyme that catalyzes the activation of acetate for use in lipid synthesis and energy generation. It has no known function in relation to placenta.

The four other genes presented a much less convincing ASE pattern and were probably false positives. Three of them (*DISC1*, *C9orf93*, *TF*) were present on the Illumina array (see below) and had low expression levels (average log_2 _fluorescence lower than 11.25). In conclusion, the Sequenom platform can detect ASE and imprinting, but no new imprinted gene was found in this study.

### ASE Illumina Array

To test more candidate genes, we increased our screening throughput by using the ASE BeadArray™ (Illumina, Inc., USA). With this technique a total of 1536 SNPs, located in 932 genes (214 expected to be expressed in placenta, see Methods) (Additional file 1: Supplemental Table S2), were tested for ASE and imprinting across 23 of the family-trios. The candidate imprinted genes included ten orthologues of known murine imprinted genes whose status was unknown in human, 124 orthologues of 600 mouse candidate imprinted genes [8], ten human candidate imprinted genes [9], and 18 known control imprinted genes [6,13,21] (Additional file 1: Supplemental Table S2). Genes specifically expressed in the placenta compared to other tissues and genes differentially expressed according to the birth weight may influence fetal growth and so may also be imprinted. We therefore tested 46 such genes [22]. The remaining 1179 SNPs (718 genes) on the array were chosen for unrelated research purposes and were thus randomly selected in terms of this study. This study also duplicated 38 genes from the Sequenom analysis on the same samples.

### Comparison of platforms

**Figure 1:**
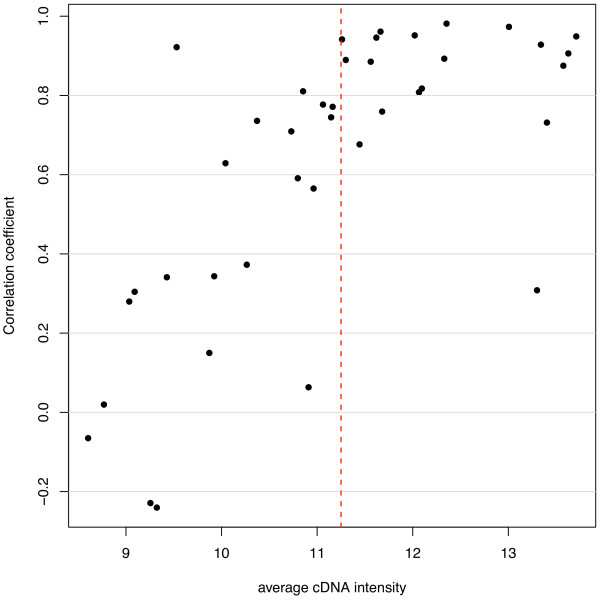
**Correlation of Sequenom and Illumina allele quantification**. Pearson's correlation coefficient (r) is calculated for the allele-specific quantification on the two platforms and plotted against cDNA intensity (average log_2 _fluorescence for the 23 placentas) on Illumina. The correlation drops when cDNA average intensity is lower than the 11.25 cut-off (dashed red line).

For comparison, we analysed the results obtained for the 38 genes tested on both platforms for the same family-trios (Figure 1). These results were used to determine the minimum cDNA intensity necessary for the Illumina platform to correlate for ASE with the Sequenom system, i.e. reliable Illumina allelic expression quantification. A cDNA intensity threshold of 11.25 units (average log_2 _fluorescence) was chosen; below this value, ASE correlation was noted to be weaker (Figure 1). The 576 SNPs with average cDNA intensities above the threshold on the Illumina arrays are listed in Additional file 1: Supplemental Table S3.

### Illumina array sensitivity for ASE detection

**Figure 2:**
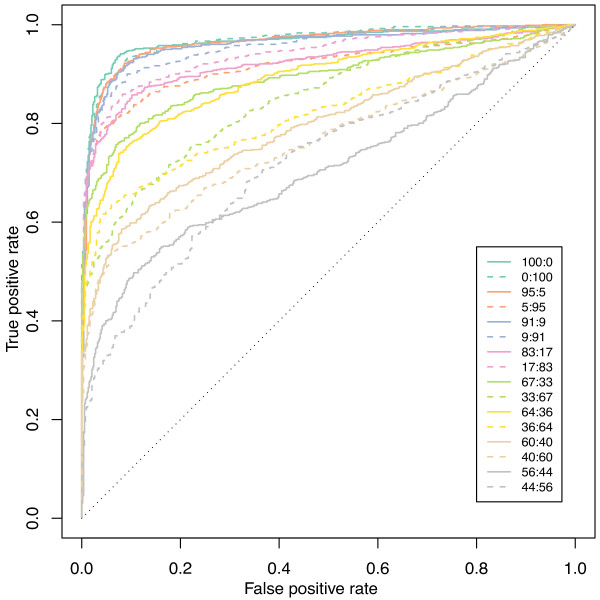
**ROC plots for the mixture control data set**.

**Figure 3:**
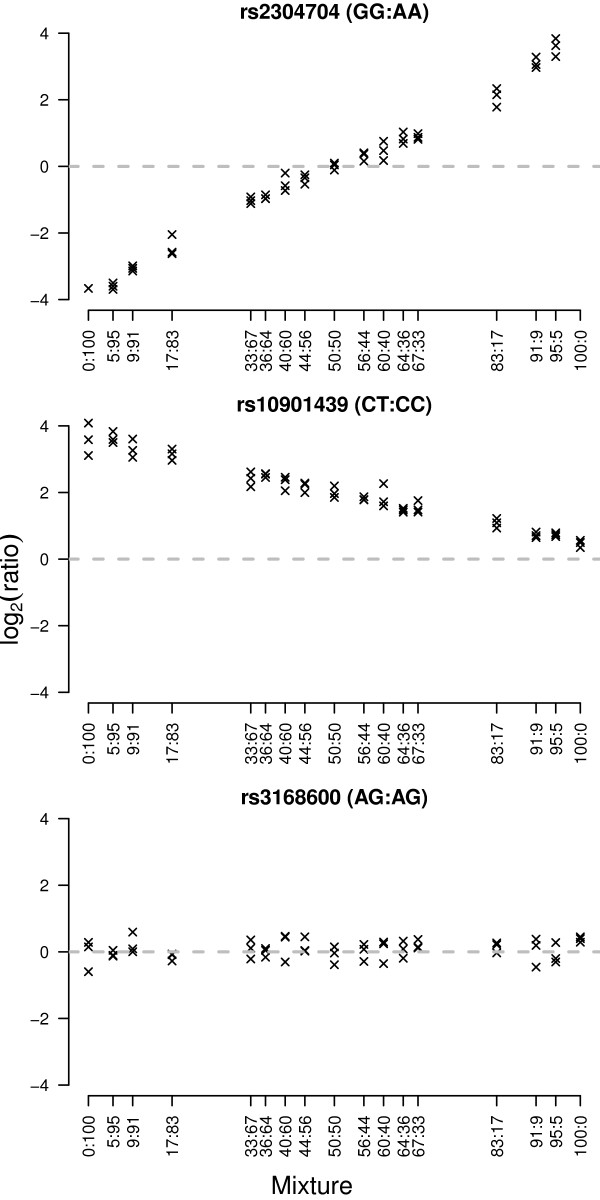
**Log-ratios for three SNPs tested in different DNA proportions of two control HapMap samples (NA12892:NA19092, from 0:100 to 100:0) on the Illumina ASE Beadarray**. From top to bottom, we see SNPs that exhibit extreme allelic imbalance, intermediate allelic imbalance and no allelic imbalance, respectively. The first two examples are true positives while the last one is a true negative for our sensitivity and specificity analysis.

To assess the capacity of the Illumina BeadArray™ ASE platform to detect ASE, we hybridised varying proportions of homozygous and heterozygous DNAs on the array (Figures 2, 3 and Methods). These 'mixture curves' show that this platform performs well to detect imprinting and strong ASE (≥ 66-34 ratio) (mean 66-34/34-66 area under the ROC curve ≥ 0.81) but less well to detect moderate ASE (≤ 60-40 ratio) (area under the ROC curve ≤ 0.77) (Figure 2).

### Illumina array sensitivity for imprinting detection

**Figure 4:**
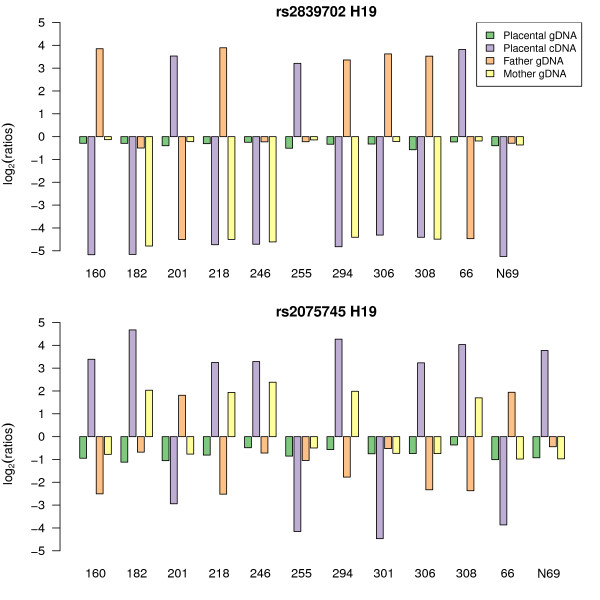
**Informative samples for SNP rs2839702 (Fig. 2A) and rs2075745 (Fig. 2B) in the human *H19 *imprinted gene**. For each informative (heterozygous placental genomic DNA) SNP, allelic log-ratios were plotted to compare gDNA and cDNA results. Each family trio is represented by a number on the X-axis and consists of placental gDNA (green), placental cDNA (purple), paternal gDNA (orange), and maternal gDNA (yellow). In the placental gDNA, both alleles occur almost equally and the log-ratio is close to zero. *H19 *is an imprinted gene. Hence in the placental cDNA, only one allele is expressed. The sign of the log-ratio for the cDNA sample changes depending upon the allele expressed. Imprinted genes cDNA log-ratio will show a typical oscillation of signal across the y-axis because it is not the allele that is important but its parent-of-origin [21]. When at least one parent is homozygous for the SNP under study, the parent-of-origin of the expressed allele can be ascertained. In the case of *H19*, the maternal allele is the one expressed as expected (i.e. for homozygous parents gDNA, the maternal gDNA allelic log-ratio has the same sign as the placental cDNA log-ratio and the paternal gDNA allelic log-ratio has the opposite sign to the placental cDNA).

**Figure 5:**
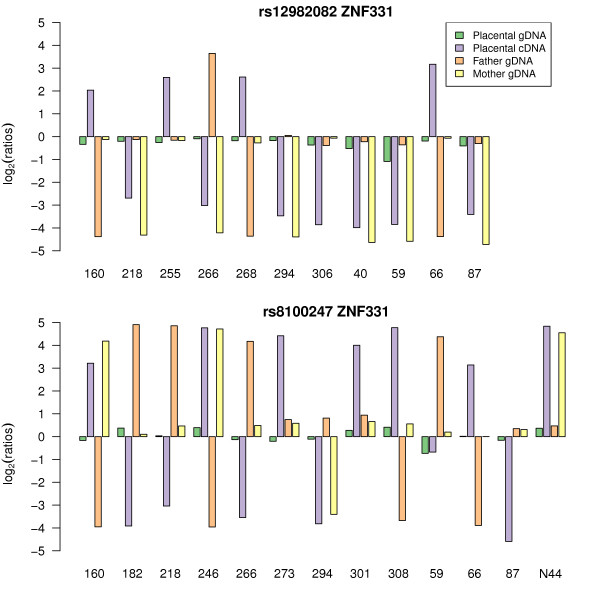
**Monoallelic maternal expression of *ZNF331 *as detected by two SNPs rs12982082 and rs8100247**. Bar chart designed as in Figure 4.

Having demonstrated the ability of the Illumina array to quantify strong ASE, we analysed the expression pattern of the 18 imprinted control genes present on the array (Table 2). To detect differential allelic expression, we designed a statistical test (ASE test, see Methods). Being an extreme form of ASE, we should detect imprinting easily if the imprinted gene is sufficiently expressed in human term placenta. Eleven imprinted control genes had a mean cDNA intensity >11.25 units (average log_2 _fluorescence). Eight genes - *H19 *(Figure 4), *PEG3, DLK1, PLAGL1, PEG10, MEST, IGF2AS *and *ZNF331 *(Figure 5, see below) - displayed a pattern characteristic of imprinting (parent-of-origin dependant monoallelic expression). One imprinted control gene, *GNAS*, was tested by two SNPs, rs3730171 and rs8386, which both had hybridisation intensities above 11.25. Only one placenta was heterozygous for each of the *GNAS *SNPs, and those two different placentas showed biallelic *GNAS *expression (Table 2). So, as found by others [23], *GNAS *was not imprinted in human term placenta. For *PHLDA2*, only one informative trio was available and showed maternal expression as expected (both parents were heterozygous in the other case). *IGF2R*, was found to be biallelic for 13 informative samples, as expected in human term placenta [24].

**Table 2:** Imprinted control genes, expression threshold and analysis by our statistical ASE test on the Illumina array.

SNP ID	Gene	Chr	Average intensity across all samples	Total number of heterozygous samples (hets)	Hets showing ASE: p < 0.01 & |lfc|>0.585	Percentage of hets which show ASE
rs2075745	*H19*	11	14.01	12	12	100%
rs1860565	*PEG3*	19	13.63	9	9	100%
rs1802710	*DLK1*	14	13.57	15	14	93%
rs3730171	*GNAS*	20	13.54	1	0	0%^†^
rs2839702	*H19*	11	13.48	11	11	100%
rs998075	*IGF2R*	6	12.78	13	0	0%*
rs8100247	*ZNF331*	19	12.61	13	12	92%
rs9373409	*PLAGL1*	6	12.48	11	9	82%
rs13390	*PHLDA2*	11	12.33	2	2	100%
rs10863	*MEST*	7	12.32	4	4	100%
rs12982082	*ZNF331*	19	12.27	12	10	83%
rs13073	*PEG10*	7	12.09	11	10	91%
rs8386	*GNAS*	20	11.97	1	0	0%^†^
rs1055359	*PEG3*	19	11.8	10	10	100%
rs1003483	*IGF2AS*	11	11.32	8	7	88%

rs854541	*PPP1R9A*	7	11.2	13	0	0%
rs2285185	*L3MBTL*	20	10.97	10	1	10%
rs2171492	*CPA4*	7	10.57	12	0	0%
rs2071970	*L3MBTL*	20	10.51	11	0	0%
rs854524	*PPP1R9A*	7	10.41	10	0	0%
rs8234	*KCNQ1*	11	10.18	10	0	0%
rs1049846	*PLAGL1*	6	10.06	13	3	23%
rs1800504	*GRB10*	7	9.98	14	0	0%
rs3741208	*IGF2AS*	11	9.67	10	0	0%
rs367035	*SLC22A18*	11	9.09	13	0	0%
rs3816800	*ATP10A*	15	9.09	15	0	0%
rs1570070	*IGF2R*	6	9.06	0	0	0%
rs1800900	*GNAS*	20	8.86	9	2	22%
rs2066710	*ATP10A*	15	8.8	16	0	0%

Imprinted genes tested on the array are listed alongside the exonic SNP used and the intensity obtained for the cDNA (average log_2 _fluorescence for the 23 placentas). The fore last column shows the number of heterozygous samples that are significant for ASE (p < 0.01) and that have a good probe hybridisation signal on the array (absolute log-fold change (lfc) >0.58). Examination of the last column (percentage of heterozygous placentas which exhibit statistically significant ASE) shows the difference for reliable detection of imprinting above or below the 11.25 average intensity threshold. *IGF2R is known to be biallelically expressed in human term placenta. ^†^The two heterozygous *GNAS *placentas showed biallelic expression.

For SNPs of imprinted control genes with intensities <11.25, the imprinting pattern became less consistent (Table 2), confirming the value of the threshold determined by the comparison of allelic expression for genes present on both platforms.

### Allele specific expression

Having established that the Illumina system could detect imprinting and strong allelic expression imbalance, we examined all the genes for evidence of ASE. SNPs were considered to show statistically significant ASE if they satisfied the following criteria: average cDNA intensity across all samples >11.25; showed allelic imbalance in expression according to our test (see Methods) in at least 80% of homozygous cDNA samples; and showed allelic imbalance in expression according to our test (see Methods) in at least two heterozygous cDNA samples.

576 out of 1536 SNPs on the array passed the 11.25 intensity threshold indicating sufficient expression in the term placenta for reliable ASE detection (Table 3 and Additional file 1: Supplemental Table 3 for full list). Of these 576 SNPs, 497 (86%) were polymorphic in our population for at least two individuals and so were informative for the detection of ASE. 261 SNPs passed the additional signal-based quality control criteria (see Methods and Table 3). Using our statistical test, ASE was detected in 56 out of these 261 SNPs. Of these, 44 SNPs targeted 39 candidate genes and 12 SNPs targeted nine control imprinted genes (Table 3).

**Table 3:** Summary of the DNA and cDNA genotyping for the Illumina assay.

	Description	Genes	SNPs
A	Tested on the array	932	1536
B	Above intensity threshold (11.25)	446	576
C	As in B with at least two heterozygous samples	393	497
D	As in C with good quality probe hybridisation in homozygotes	214	261
E	As in D with at least two heterozygotes significant for ASE (p < 0.01)	49	56
F	As in E for the candidate genes only	39 (18.2%)	44 (16.9%)

Numbers of SNPs and genes that are (A) tested on the Illumina array, (B) have passed the intensity cut-off, (C) have sufficient heterozygous placentas, (D) have passed hybridisation probe quality controls and (E) for which our statistical test detected ASE. Column F is the same as E but without taking into account the imprinted control genes.

Five different types of ASE were looked for in the 44 SNPs targeting 39 genes: (1) imprinted, monoallelic expression in a parent-of-origin dependent manner; (2) ASE in a parent-of-origin manner, also called partial imprinting; (3) preferential ASE, where the same allele is expressed at higher levels in each heterozygote whatever its parent-of-origin; (4) random monoallelic expression, where one of the two alleles is completely silenced in a random way; (5) random ASE, where different alleles are expressed at higher levels in different heterozygotes without parental bias (Table 4). To determine which of these patterns of allelic imbalance in expression was detected, log-ratios of informative family-trios were plotted as described in Figure 4 and subjectively categorised (Additional file 3). The patterns of allelic imbalance identified for the 56 SNPs are reported in Table 4.

**Table 4:** The ASE pattern of genes reaching statistical significance (Illumina Assay).

rsID	Name	Chr	Imprinting status	Average intensity >11.25	Number of hets	Number of hets with p < 0.01	Pattern of ASE	Alleles
rs1802710	*DLK1*	14	control	13.57	15	14	imprinting	
rs8100247	*ZNF331*	19	control	12.61	13	12	imprinting	
rs2075745	*H19*	11	control	14.01	12	12	imprinting	
rs2839702	*H19*	11	control	13.48	11	11	imprinting	
rs1082	*PHACTR2*	6	candidate	12.09	14	10	partial imprinting	
rs12982082	*ZNF331*	19	control	12.27	12	10	imprinting	
rs13073	*PEG10*	7	control	12.09	11	10	imprinting	
rs1055359	*PEG3*	19	control	11.8	10	10	imprinting	
rs9373409	*PLAGL1*	6	control	12.48	11	9	imprinting	
rs1860565	*PEG3*	19	control	13.63	9	9	imprinting	
rs2309428	*TJP2*	9	candidate	12.86	9	8	random ASE	
rs178077	*SNAP29*	22	candidate	11.95	10	7	random ASE	
rs1003483	*IGF2AS*	11	control	11.32	8	7	imprinting	
rs8585	*UBE2V1*	20	candidate	12.36	13	6	preferential	A>G
rs1130663	*CD151*	11	candidate	12.44	18	5	random ASE	
rs4664114	*FMNL2*	2	candidate	11.51	14	5	random ASE	
rs4944960	*XRRA1*	11	candidate	12.43	12	5	preferential	G>C
rs2282336	*TJP2*	9	candidate	12.32	9	5	random ASE	
rs6633	*CDK2AP1*	12	candidate	11.34	8	5	random ASE	
rs4614	*VPS11*	11	candidate	13.09	13	4	random ASE	
rs3817672	*TFRC*	3	candidate	12.07	13	4	* preferential	T>C
rs2905	*C14orf130*	14	candidate	11.33	12	4	random ASE	
rs12190287	*TCF21*	6	candidate	12.88	10	4	random ASE	
rs3809865	*ITGB3*	17	candidate	12.09	10	4	random ASE	
rs10863	*MEST*	7	control	12.32	4	4	imprinting	
rs915894	*NOTCH4*	6	candidate	13.44	16	3	* preferential	A>C
rs754615	*CAST*	5	candidate	13.71	14	3	preferential	G>C
rs838896	*SCARB1*	12	candidate	12.62	10	3	random ASE	
rs5758651	*TCF20*	22	candidate	12.4	10	3	random ASE	
rs11699879	*NCOA3*	20	candidate	12.68	9	3	random ASE	
rs838891	*SCARB1*	12	candidate	11.85	9	3	random ASE	
rs2425009	*MYH7B*	20	candidate	11.68	9	3	random ASE	
rs9749449	*ZNF211*	19	candidate	11.71	6	3	random ASE	
rs4797	*SQSTM1*	5	candidate	14.48	18	2	preferential	G>A
rs1128933	*MAN2C1*	15	candidate	12.71	16	2	preferential	C>T
rs10277	*SQSTM1*	5	candidate	11.78	16	2	preferential	G>A
rs2249057	*NM_006031*	21	candidate	13.31	13	2	* preferential	C>A
rs7226091	*MGC16597*	17	candidate	11.83	13	2	* preferential	C>G
rs1043618	*HSPA1A*	6	candidate	13.6	12	2	random ASE	
rs17085249	*ELL2*	5	candidate	12.98	12	2	random ASE	
rs2255255	*CRNKL1*	20	candidate	12.86	12	2	random ASE	
rs2013162	*IRF6*	1	candidate	12.64	12	2	* preferential	C>A
rs11121567	*PGD*	1	candidate	12.03	12	2	* preferential	A>G
rs3780473	*ACO1*	9	candidate	11.67	12	2	random ASE	
rs4669	*TGFBI*	5	candidate	13.27	11	2	random ASE	
rs7242	*SERPINE1*	7	candidate	13.01	11	2	random ASE	
rs2788478	*FLJ10300*	7	candidate	13.04	10	2	random ASE	
rs2271108	*DOCK5*	8	candidate	12.85	10	2	random ASE	
rs552282	*PPFIA1*	11	candidate	11.58	10	2	* preferential	C>T
rs1044116	*NOTCH3*	19	candidate	12.21	9	2	random ASE	
rs7204628	*MGC24665*	16	candidate	11.84	9	2	random ASE	
rs844	*FCGR2B*	1	candidate	13.05	8	2	* preferential	C>T
rs11156878	*KIAA0391*	14	candidate	12.15	5	2	random ASE	
rs12780	*PRDM8*	4	candidate	12.02	5	2	random ASE	
rs5919	*ITGB3*	17	candidate	11.55	4	2	random ASE	
rs13390	*PHLDA2*	11	control	12.33	2	2	imprinting	

All SNPs had an average intensity above 11.25 and all or a subset of heterozygous samples had a significant statistical ASE test (p < 0.01). For each SNP, the ASE pattern (see text for details) was subjectively determined by examination of the bar charts, designed as in Figure 4, for all heterozygous samples. In case of preferential expression, the allele that was more expressed is indicated in the last column. *For these SNPs, the preferential bias is weaker.

For the genes exhibiting a statistically significant ASE effect, an imprinting ASE pattern was found for all control imprinted genes and *ZNF331 *(encoding a zinc finger protein on chromosome 19q13.41, RefSeq NM_018555). Using two SNPs on the Illumina system, rs8100247 (exon 1, 5'UTR) and rs12982082 (exon 2, 5'UTR), *ZNF331 *showed a consistent pattern of maternal origin for the expressed allele (Figure 5). These results strongly suggest that the *ZNF331 *transcripts targeted by the SNPs present on the array are imprinted and maternally expressed in the human term placenta. RT-PCR amplification and Sanger sequencing of SNPs in two exons of the *ZNF331 *transcript (exon 1, 5'UTR and exon 7, CDS) confirmed the maternal expression seen with the Illumina method (Additional file 4).

**Figure 6:**
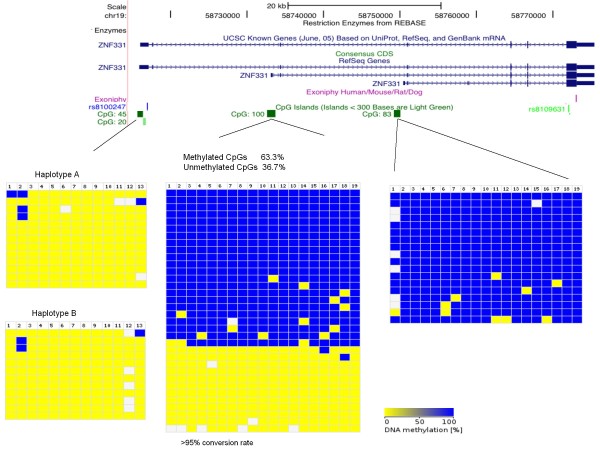
**Methylation levels of the CpG islands within *ZNF331***. The figure shows the position of *ZNF331 *and the CpG islands in the UCSC genome browser. In the bottom part of the figure, the methylation pattern of the three CpG islands studied is represented. Bisulphite sequencing data was compiled with the BDPC webtool [53]. The blue colour indicates metylated CpGs and the yellow colour unmethylated CpGs. Each column represents a single CpG site and each row represents one clone. The sequences for two CpGs (45 and 83) were sorted according to their genotype.

*ZNF331 *is thus imprinted in human term placenta. Usually differentially methylated CpG islands are necessary to achieve imprinting. The four 'promoter' CpG islands (Figure 6) that we could find at the 5' extremity of each isoform of *ZNF331 *were tested for differential methylation. We have been able to amplify 3 CpG islands in bisulphite-treated human term placental DNA. The CpG 100 (promoter of *ZNF331 *second longest isoform) showed a typical DMR pattern (amplicons are either fully methylated or unmethylated). Unfortunately, no SNP was present in the amplified regions to determine the parental specific methylation of the DMR.

**Figure 7:**
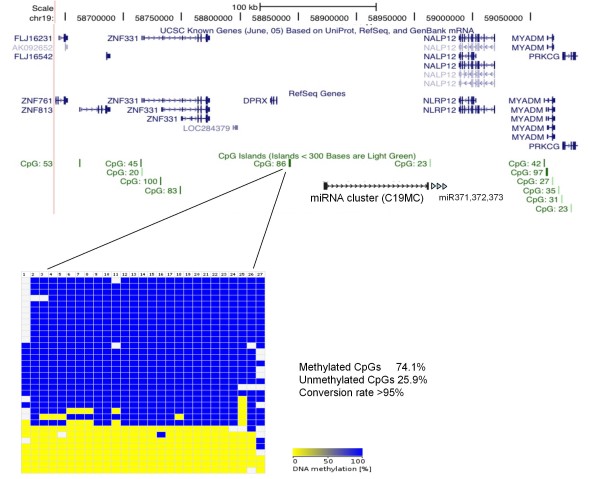
**Methylation levels of CpG86 in *ZNF331 *locus**. The figure, generated with UCSC genome browser, indicates the position of the CpG island in relation to the location of *ZNF331*, its neighbouring genes and miRNAs. Bisulphite sequencing data was compiled with the BDPC webtool as in Figure 6. No SNP was present in the amplified sequence.

As imprinted genes are often found in clusters, we analysed the CpG island closest to *ZNF331 *for differential methylation (Figure 7). We found this CpG (located between the *DPRX *gene and the *C19MC *miRNA cluster and called CpG 86) to show a typical DMR pattern. Again, no SNP was available to test its parent-specific methylation. So these data suggests that *ZNF331 *could be part of a new imprinted locus with (at least) two DMRs.

**Figure 8:**
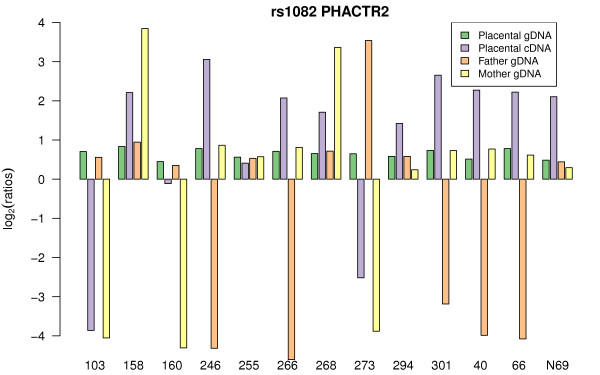
**rs1082 *PHACTR2***. The ASE pattern of rs1082-*PHACTR2 *is consistent with partial imprinting (maternal expression bias). Bar chart designed as in Figure 4.

The second imprinted candidate, based on our Illumina array ASE test, is *PHACTR2 *(phosphatase and actin regulator 2 gene). The *PHACTR2 *gene contains the SNP rs1082, located in the 3'UTR of the gene, and 10 of 14 informative placentas exhibited ASE dependent on the parent-of-origin of the allele (Figure 8). The fact that the cDNA log-ratio is always smaller than the one seen for homozygous gDNA suggests partial imprinting. Parental genotyping shows that it is always the maternal allele that is more highly expressed.

**Figure 9:**
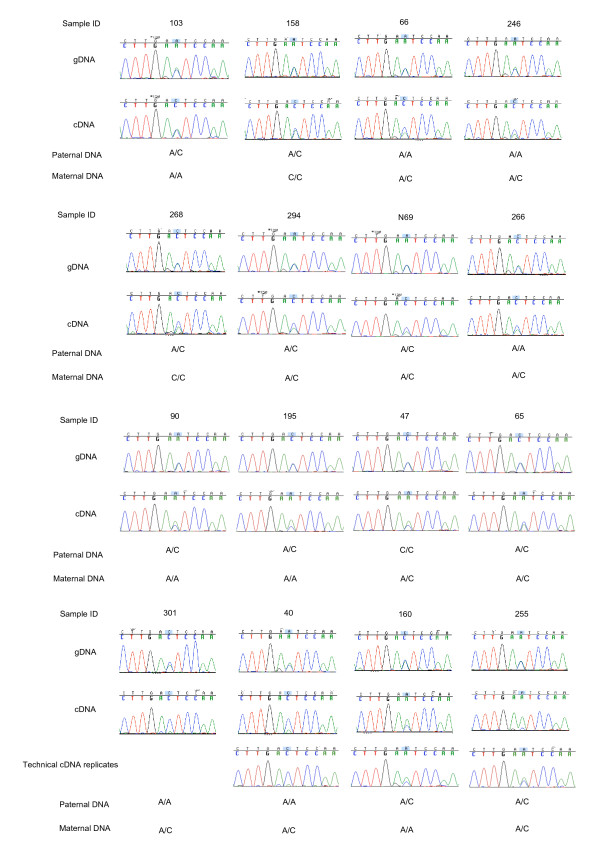
***PHACTR2 *cDNA allelic expression ratio is biased towards the maternal allele**. Sequences for rs1082 (3'UTR) of all available informative term placenta samples in cDNA and gDNA are shown with corresponding maternal and paternal genotyping data.

Partial imprinting of *PHACTR2*, was confirmed using Sanger sequencing on fourteen placental samples. A recurrent maternal bias was seen between gDNA and cDNA sequence traces overlapping the same *PHACTR2 *3'UTR SNP (rs1082) (Figure 9). These sequencing results confirm the partial imprinting of *PHACTR2 *in human term placenta and the ability of the Illumina BeadArray™ platform to detect ASE.

**Figure 10:**
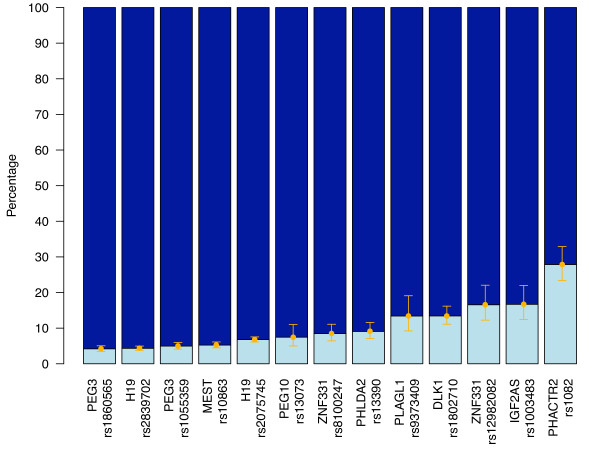
**Lack of complete repression of the silenced allele for imprinted genes**. Average quantification of the expressed allele (dark blue) and of the silenced allele (light blue) in all informative samples for all expressed control imprinted genes (*PEG3, H19, MEST, PEG10, PHLDA2*, *PLAGL1, DLK1 and IGF2AS*), for *ZNF331 *and for *PHACTR2*.

To examine further the strength of allelic silencing observed in our data for all imprinted genes (i.e. complete to partial imprinting), raw allelic values, averaged over all cDNAs from informative individuals, were plotted for the imprinted control genes and the most significant imprinted candidate gene on the array (Figure 10). The difference of expression between the two alleles of a known imprinted gene varies from a 23-fold difference (*PEG3*-rs1860565) to a 6.4-fold difference (*DLK1*-rs1802710). For *ZNF331*, the difference is 5-fold for rs12982082 and 11-fold for rs8100247, and for the partially imprinted gene *PHACTR2*, 2.6-fold (Figure 10). These results show that the repression of the silenced allele is not complete for all control imprinted genes and that there is a continuum from 'complete imprinting' to 'partial imprinting'. While our results could suggest that it is likely that most or all 'completely imprinted' genes have already been found in the placenta (see discussion), our *PHACTR2 *study indicates that partially imprinted genes could have been labelled as 'biallelic' and that several other partially imprinted genes could still be found and characterised.

Of the 56 SNPs (49 genes) statistically significant with our ASE test, 12 SNPs were located in nine of our selected imprinted control genes (*DLK1, H19, IGF2AS, MEST, PEG3, PEG10, PLAGL1, PHLDA2, ZNF331*) and one SNP was localised in *PHACTR2 *and its ASE pattern was compatible with partial imprinting (see above).

Of the 43 remaining SNPs (39 genes), six (five genes) showed an allelic preferential pattern when visually examined (*UBE2V1, XRRA1, CAST, SQSTM1, MAN2C1*; see Additional file 5) and eight showed possible allelic preference (Table 4 and Additional file 3). The others were too variable to be assigned a precise ASE pattern and could correspond to random allelic bias, epistatic allelic preferential expression, bipolar ASE (see Discussion) [25] or false positives.

To investigate these 43 significant ASE SNPs further, we used the Genevar Database (T-P. Yang and E. Dermitzakis, manuscript in preparation) to check for *cis*-effects for the same 43 SNPs and 39 genes in LCLs from eight HapMap3 populations (CEU, CHB, JPT, GIH, MKK, YRI, LWK, MEX) (B. Stranger and E. Dermitzakis, manuscript in preparation). The database allows searching for a specific SNP-gene pair showing an expression quantitative trait locus (eQTL), for *cis*-eQTLs arising from a specific SNP or for *cis*-eQTL SNPs acting on a specific gene [19,20]. In other words, using this database, we can look for the effect of a specific SNP on the transcription of a specific gene (SNP-gene pair eQTL), the effect of a specific SNP on all (tested) genes located in the vicinity of this SNP (SNP cis-eQTL) or we can examine the effect on the transcription level of a specific gene by any SNPs located in the vicinity of this gene (gene cis-eQTL). We can also examine transcription level of a specific gene by any tested SNPs in the vicinity of the gene (*cis*-effect) or far away from the gene (*trans*-effect). We found respectively nine, four and two of these types of eQTLs in the database corresponding to our ASE SNPs and genes. This suggests that 15 of our 43 ASE SNPs (35%) could be genuine examples of allelic preferential expression in two different human tissues, namely term placenta and LCLs [26]. Five of the 15 eQTLs were found to overlap with the six ASE significant SNPs-genes pairs showing a prominent allelic preferential bias (see Additional file 5): four SNP-gene pair eQTLs (*SQSTM1*-rs10277, - rs 4797; *MAN2C1*-rs1128933; *CAST*-rs754615) and one gene *cis*-eQTL (*XRRA1 *(rs4944960 does not exist in Genevar)). *UBE2V1 *showed only a marginal gene-eQTL effect while rs8585 was also not in the Genevar database. So all four SNP-gene pairs tested in both tissues and four of the five (80%) genes showing significant preferential allelic bias in placenta also showed a strong preferential allelic bias in LCLs. In addition to the validation of our placental experiments, this overlap strongly suggests that our most significant preferential allelic biases (Additional file 5) are genuine (and probably ubiquitous).

## Discussion

Our data demonstrate that quantitative genotyping technologies like the Sequenom Mass Spectrometer and Illumina Beadarray™ platforms are reliable in the detection of strong allelic skewing as shown by the correct identification of known imprinted genes and different patterns of ASE from the data. We have found that allelic imbalances in expression are common in the candidates we analysed in the human term placenta and that true monoallelic expression (imprinted or random) is a rare phenomenon. We found only one new 'partially imprinted' gene (0.5%), while ASE was present in 18% of the candidate genes passing our quality control criteria. Such levels of ASE are similar to the results seen in cell lines or other somatic tissues [12-15,21].

Our data show that *ZNF331 *is imprinted in human term placenta and expressed from the maternal allele. *ZNF331 *(also known as *ZNF463*) was first shown to exhibit monoallelic expression in a parent-of-origin manner in lymphoblastoid cell lines [13,21], although the parent-of-origin orientation of *ZNF331 *in these studies was not clear (paternal in one study, maternal in the other). No obvious explanation would easily explain this discrepancy. It would be interesting to study *ZNF331 *allelic mode of expression in a range of human tissues and in an isoform-specific manner.

In addition, our methylation results (Figures 6 and 7) suggest that *ZNF331 *could be part of a new imprinted locus with (at least) two DMRs. Recently, Tsai and colleagues showed the same DMR pattern for the CpG 86 (the one located between *DPRX *and *C19MC *genes) independently suggesting that the '*ZNF331-C19MC*' locus could be a new imprinted locus [27]. *C19MC *seems to be mainly expressed in placenta and fetal brain [28-30], a pattern that would perfectly suit the expression of an imprinted gene. Finally, *ZNF331 *and *C19MC *seem to be primate specific genes (no murine orthologue for *ZNF331 *was found using Ensembl or UCSC; and *C19MC *is primate-specific [28-30]). This probably explains why this locus was not found in previous mouse genome wide screens for imprinted loci. Hence, all aggregated results suggest a possible importance of the *ZNF331-C19MC *locus in human placental-fetal growth, metabolism and cancer. Being primate specific genes, the determination of their functional role in development will be a challenge.

We found *PHACTR2 *to be partially imprinted in placenta (Figures 8 and 9). *PHACTR2 *is located on chromosome 6q24.2, 114 kb from *PLAGL1 *a known imprinted gene (previously called *ZAC1*). Loss of imprinting of *PLAGL1 *is seen in transient neonatal diabetes [31,32]. PHACTR2 is a member of a family of four actin and protein phosphatase 1 (PP1) binding proteins highly expressed in the brain [33,34]. The function of PHACTR2 in placenta is unknown. PHACTR1, 3 and 4 have roles in brain and neural tube development and in cell spreading [35,36]. Mouse strain allele specific dominant expression has been shown in brain for an isoform of *Phactr3 *(i.e., only the *Phactr3 *NMRI allele of exon 1C is expressed in NMRI/Cast heterozygous F1 progeny whatever the parent-of-origin of the NMRI allele) [37]. So, our results show that *PHACTR2 *is partially imprinted in placenta, and, with other work, suggest that the *PHACTR *gene family could be prone to complex epigenetic regulation.

In total across the two platforms, we experimentally studied 183 genes identified as candidates for imprinted expression by prior bioinformatics approaches [8,9]. Luedi et al. [8] predicted 600 genes to be imprinted out of 23,788 murine autosomal annotated genes. We have tested 155 of these 600 mouse candidates and found one that exhibited (partial) imprinting in the term placenta. In another study of these murine candidates [38], one (*KCNK9*) out of 16 genes selected from the 600 candidates was found to be imprinted in the mouse and human brain. Some of the 16 candidates tested by Ruf et al. [39] were selected due to their proximity to known imprinted genes. In our results the one gene that exhibited partial imprinting, *PHACTR2 *is located adjacent to *PLAGL1*, a known imprinted gene (previously called *ZAC1*). Combined with the prior observations that imprinted genes often occur in clusters, these data suggest that if there are more imprinted genes to be found they may lie close to other imprinted genes.

Recently, Luedi and colleagues generated a list of 156 candidate human imprinted genes [40]. Given that nearly all genes that are imprinted in human are also imprinted in the mouse, it is surprising that the mouse and human prediction lists overlap for only a few candidates. Non-coding features like repeats were used to predict candidates and it is possible that there were differences in the assembly quality of these features in the versions of the human (Ensembl version 20) and mouse (Ensembl version 16) genomes used for these studies [8,40]. It would be interesting to test the algorithms on the most recent assemblies of both genomes. None of the 28 candidates identified by mining EST databases [9] that we tested was imprinted in placenta. Thus, only one of the 183 candidates predicted by bioinformatics methods that we tested was found (partially) imprinted in placenta. The poor specificity of the bioinformatics predictions in placenta raises two possibilities: either, the bioinformatics predictions have low specificity overall and only a handful imprinted genes are still to be discovered or the predictions are correctly identifying imprinting in tissues other than placenta. Most phenotypes with a heritability compatible with imprinted gene disruption have been explained [6]. However, new imprinted genes are still being discovered:*NLRP2 *and *OSBPL1A *in placenta [15], *ZNF331 *in placenta (this work) and in LCLs [13,21], KCNK9 in brain [39,40], *DLGAP2 *in testis [40]. Hence it is possible that new imprinted genes will mainly be discovered in a tissue-specific manner and that more subtle phenotypes could be associated with their disruption.

We analysed five modes of ASE (imprinted, partial imprinting, preferential, monoallelic random, random ASE). Recently, Cheverud and colleagues suggested that different bipolar modes of ASE could exist [25,41]. Bipolar ASE shows allele specific bias depending first on the parent-of-origin of the allele and second on heterozygous or homozygous status for this allele (a mode of allelic expression inheritance that was previously only known in the callipyge sheep [42]). Considering the bipolar associated growth and metabolic phenotypes described by Cheverud et al. in the adult mouse [25], it will be interesting to explore bipolar ASE in human tissues. However, the platforms used in this study would need to test many more trios with more replicates to approach the precision required to investigate such complex ASE patterns.

Our quantitative allelic expression results for the imprinted control genes present on the array showed that the 'silencing' of the repressed allele is not always absolute (Figure 10). It is more of a continuum from complete silencing (e.g. *PEG3, H19*, and *MEST*) to partial silencing (e.g., *DLK1, IGF2AS *and *PHACTR2*). These results agree with the recent work of Lambertini et al, who showed some expression of the 'silenced' allele in human term placenta [23]. For example, for *DLK1 *such incomplete silencing was present for several individuals on both the Illumina and Sequenom platforms. We also documented one placenta showing nearly 50-50 biallelic expression of *DLK1 *(data not shown). Sakatani and colleagues have already described such complete relaxation of imprinting for *IGF2 *[43]. As them, we also found one term placenta (10%) showing biallelic expression for *IGF2 *(data not shown). The pathological importance of such loss of imprinting in a 'healthy' human term placenta is not known. Hence, our quantitative allelic expression in imprinted genes suggest that term placenta can rarely show complete loss of imprinting for *IGF2 *and *DLK1*, that parent-specific allelic expression is a continuum from complete silencing of one parental allele to a parentally biased expression of the two alleles, and that some partially imprinted genes could still be found.

## Conclusion

Both Sequenom MassArray and Illumina GoldenGate platforms were sensitive enough to study imprinting and strong ASE (= 66-34 ratio). Four patterns of ASE (imprinting, partial imprinting, preferential ASE, and random ASE) were found in human term placenta. Prior bioinformatics predictions were not useful to identify new imprinted genes in the human term placenta, suggesting that screening of other tissues and/or refinement of prediction methods may be necessary. We showed that *ZNF331*, a known lymphoblastoid cell imprinted gene, is maternally expressed in human term placenta. The possibility that *ZNF331 *is ubiquitously imprinted argues for further study of its function in metabolism, behaviour, fetal development and cancer. We showed that two potential DMRs are present in the primate-specific ZNF331-C19MC locus. We showed that *PHACTR2*, a neighbour of the imprinted gene *PLAGL1*, is partially imprinted in human placenta, the maternal allele being more highly expressed. Such a result calls for further evaluation of the allelic expression landscape of the complex and gene-rich human *PHACTR2*-*PLAGL1 *locus. Demonstration of incomplete silencing of the repressed allele for several control imprinted genes and *PHACTR2 *indicates that partially imprinted genes can be identified with appropriate screening tools. On the Illumina array, 39 candidate genes were statistically significant for our ASE test (18% of the candidate genes passing quality controls). Finally, our results suggest that ASE is a common variability factor in placental tissue and should be thoroughly studied in normal and pathological pregnancy.

## Methods

### DNA and RNA preparation

Placental trio samples consisting of placental tissues with corresponding maternal and paternal blood samples were collected from consenting pregnant mothers of European ancestry at Queen Charlotte's and Chelsea Hospital (local ethics approval 2001/6029). Samples were washed in sterile PBS and snap frozen in liquid nitrogen. A set of 24 trios was randomly chosen from the tissue bank. For one trio, the genotyping of parental DNAs revealed it was not a biological family and parental information was removed from subsequent analyses. Genomic DNA (gDNA) was extracted from placental tissue samples and peripheral blood using standard phenol-chloroform separation. Total RNA was extracted from homogenised placental tissues using Trizol (Invitrogen). RNA was treated with Turbo DNA-free (Ambion) to minimize genomic DNA contamination, concentrated and further cleaned with RNeasy MinElute columns (Qiagen). Total RNA and gDNA were quantified using a spectrophotometer and either Quant-iT™ RiboGreen^® ^RNA assay or Quant-iT™ PicoGreen^® ^DNA assay (Invitrogen). For the Sequenom platform, single stranded cDNA was synthesised from 250 ng of RNA with Superscript III reverse-transcriptase (RT) (Invitrogen) and random hexamers. Duplicate sets of samples were processed with RT omitted to detect genomic contamination of the RNA. Both sets were diluted at 1/50 before being assayed. For the Illumina platform, double stranded cDNA was synthesised from 250 ng of total RNA. The first strand was synthesised with Superscript™ III RT (Invitrogen) and randoms hexamers. The second strand was synthesised with DNA polymerase I (Invitrogen) and ribonuclease H (Invitrogen). The 96-well plates containing the double-stranded cDNA samples were cleaned using Multiscreen^® ^PCR_μ96 _filtration plates (Millipore) before being assayed on the Illumina ASE array.

### Sequenom Assay

Control and candidate genes were selected for quantitative genotyping using the homogeneous MassEXTEND (hME) assay (Sequenom, Inc.) according to their expression levels in placenta in the Unigene database http://www.ncbi.nlm.nih.gov/UniGene. The SNPs chosen were located in the 5'UTR, 3'UTR, or exons and had a minor allelic frequency (MAF) >0.15 in our population of European ancestry (dbSNP Build ID: 125 and 126, http://www.ncbi.nlm.nih.gov/SNP/. One SNP per gene was studied for seven biallelic controls, six human imprinted genes, seven orthologues of mouse imprinted genes, 26 human candidates [9], and 100 orthologues of mouse candidate imprinted genes [8] (Additional file 1: Supplemental Table S1).

The MassArray system (Sequenom, Inc.) consists of a primer extension assay for genotyping and quantitation of alleles by MALDI-TOF (matrix-assisted laser desorption/ionization time-of-flight) mass spectrometry [44]. Three different primers (two for amplification and one allele-specific MassEXTEND primer) were designed for each targeted SNP using SpectroDesigner (Sequenom, Inc.) within the exon or the UTRs. PCR amplification was followed by shrimp alkaline phosphatase (SAP) treatment. The primer extension reaction generates different mass signals for the two alleles. SNPs were multiplexed in threes according to the termination mix used. Samples were purified using SpectroCLEAN (Sequenom). Samples were then spotted on the chip (SpectroCHIP, Sequenom) with the MassArray nanodispenser and analysed by SpectroREADER mass spectrometer (Sequenom). Genotypes were called by the proprietary software (SpectroTyper v2.0). Primer sequences and thermocycling conditions are available upon request.

### Sequenom analysis

To find new imprinted genes or ASE, genotype calls were filtered to include only the genotypes that had been called with the "conservative" rating. The percentage of genotyping assays called in this way for each SNP was referred to as the *success rate *(SR) and was calculated for gDNA and cDNA. The ratio of cDNA to gDNA SR was used to filter out lowly expressed genes. Genotyping with a SR ratio ≥ 75% was taken forward in the analysis. Calls were then filtered to select trios with heterozygous placental genomic DNA. On these trios, a one-tailed paired *t*-test was used, for each SNP, to compare allelic quantification of the two alleles in placental cDNA and in placental genomic DNA. *P*-values were then adjusted using the Benjamini-Hochberg method to control false discovery rate [45]. The analysis was carried out in R [46].

### Illumina Assay

The oligo pool of 1536 SNPs of the GoldenGate ASE Array (Illumina, Inc., USA) included 18 known imprinted genes, four housekeeping genes, 11 genes shown to be preferentially expressed in the placenta [22], ten genes predicted to be imprinted in humans [9], ten orthologues of mouse imprinted genes, 35 genes that are differentially expressed according to infant weight [22], six polycomb genes and 124 human orthologues of genes predicted to be imprinted in mouse [8]; all of which were selected based on their placental expression in the Unigene database http://www.ncbi.nlm.nih.gov/UniGene (see Additional file 1: Supplemental Table S2 for a list of SNPs and genes). All SNPs chosen were located within the exons or UTRs of the targeted genes in order to be present in the spliced mRNA. SNPs with the highest minor allele frequency (MAF) in our population in the single nucleotide polymorphisms database (dbSNP Build ID: 125 and 126), http://www.ncbi.nlm.nih.gov/SNP/ and best Illumina design scores in our candidate genes were preferred. Alleles were differentiated by Cy3 and Cy5 labelled probes [47].

Paired gDNA (250 ng) and double-stranded cDNA (made from 250 ng total RNA, see above) were identically processed and hybridised to a standard 96-sample Sentrix Array Matrix according to the manufacturer's instructions for GoldenGate genotyping assays (Illumina, Inc., USA) [48]. After hybridisation for 16 hours, arrays were scanned with a Bead Station (Illumina, Inc., USA). For each placental sample, gDNA and cDNA were assayed on the same plate, and the whole plate analysis was replicated on a different day. For two cDNA samples replicates, cDNA amplification was not obtained. Parental gDNA genotyping was performed on a separate plate (not replicated). The genotypes were called using Illumina's proprietary software (BeadStudio and GenCall) with the gDNA signals as input. The composition of the trios was imported so that Mendelian errors could be highlighted during the manual curation of the genotyping. Arrays with a low dynamic range were discarded and repeated. The raw data from this experiment is available in the ArrayExpress database http://www.ebi.ac.uk/arrayexpress under accession number E-TABM-796.

### Illumina Data Analysis

The raw Cy3 and Cy5 intensities from all beads on an array were quantile normalised between channels. Log-ratios (log_2_(Cy5/Cy3)) and average log-intensities (1/2log_2_(Cy5 × Cy3)) were calculated for each bead on each array. Outliers greater than 3 mean absolute deviations (MADs) from the median of each bead type were removed as per Illumina's standard method and the remaining values were averaged to obtain a summary log-ratio and average log-intensity for each bead type (i.e., mean of ~30 beads per SNP) on each array. The summarized data were normalised per array by median centering the log-ratios to have median zero.

To test for ASE, we used the following method. Linear models were fitted to the cDNA log-ratios to summarise the replicate observations. After empirical Bayes shrinkage of the SNP-wise variances, moderated *t*-statistics were calculated [49]. Raw *p*-values from these *t*-tests were adjusted globally for multiple testing using the method of Benjamini and Hochberg to control the false discovery rate [45]. Our criteria for ASE required that SNPs satisfy the following conditions: (1) average intensity across all samples greater than 11.25 (Illumina arbitrary fluorescence units); (2) at least 2 heterozygotes (based on BeadStudio calls from gDNA samples) with adjusted *p*-values less than 0.01 and absolute log-fold-changes greater than 0.585 and (3) at least 80% of homozygotes with adjusted *p*-values less than 0.01 and absolute log-fold-changes greater than 0.585. The intensity cut-off was based on the concordance between Illumina and Sequenom data, with probes expressed below this level less reliably quantified on the Illumina arrays (Figure 1). The log-fold-change cut-off of 0.585 was based on the mixture data (Figure 2). This experiment showed that true positives were more difficult to detect on the Illumina arrays in mixtures at or below 60:40/40:60 (equivalent to absolute log-ratios less than log_2_(60/40) = 0.585). The homozygote criteria (3) ensured that the two alleles could be reliably distinguished in the cDNA samples. All analyses were carried out in R using the beadarray [50] and limma packages [51].

### Mixture Analysis

For the control experiment, gDNA mixtures of two HapMap individuals (NA12892:NA19092) (Coriell, Camden, New Jersey, United States) were created in the following proportions: 0%:100%, 5%:95%, 91%:9%, 83%:17%, 67%:33%, 64%:36%, 60%:40%, 56%:44%, 50%:50%, 44%:56%, 40%:60%, 36%:64%, 33%:67%, 17%:83%, 9%:91%, 5%:95% and 100%:0%. Each mixture was hybridized in duplicate using the same experimental protocol. Data were preprocessed and normalised as described in the previous section.

A linear model was fitted to each SNP as described previously, and contrasts were obtained to give all pairwise comparisons between a given mixture and the 50%:50% mixture. This corrects for dye biases and systematic shifts which are present for SNPs which are either heterozygous and homozygous (i.e. AA:AB, BB:AB, AB:AA or AB:BB) or have the same genotype (AA:AA, BB:BB or AB:AB) in the two individuals. Moderated *t*-statistics were calculated using the empirical Bayes shrinkage procedure [49] to test the null hypothesis that each contrast was equal to 0 (i.e. no allelic imbalance). Sensitivity and specificity calculations were made for each contrast by ranking SNPs by their log-odds and using *a priori *genotype information on which SNPs are true positives/negatives for allelic imbalance.

Genotypes for NA12892 and NA19092 were downloaded from HapMart http://hapmart.hapmap.org/BioMart/martview, version 21, NCBI Build 35) for the SNPs on the array. SNPs with known allelic imbalances between these individuals (782), such as those which are either homozygous and different (AA:BB or BB:AA), or heterozygous and homozygous (AA:AB, BB:AB, AB:AA or AB:BB), form the true positive set. SNPs which have the same genotype for each individual (AA:AA, BB:BB or AB:AB) should not change with mixing concentration, and comprise the true negative set (533). SNPs with missing data (15 with NN calls) and those with IDs that could not be found in HapMart (206) were excluded from the analysis.

### Platform Correlation

Pearson correlation coefficients were calculated for 38 SNPs using log-ratios from samples assayed using both the Illumina arrays and Sequenom assay (log-ratios calculated as log_2 _[(seque_x+1)/(seque_y+1)]).

### Sanger sequencing

Using Primer3 http://frodo.wi.mit.edu/, one set of primers was designed to be used for both PCR and RT-PCR. Primer sequences and thermocycling conditions are available upon request. PCR and RT-PCR products were cleaned with Microclean (Microzone) and sequenced using standard ABI sequencing technology (Big Dye v1.1).

### Methylation study

Bisulphite converted gDNA samples were prepared and cleaned using the EZ DNA methylation-Gold™ kit (Zymo, CA) according to the manufacturer's instructions. For each CpG island of interest, bisulphite primers were designed using the MethPrimer webtool http://www.urogene.org/methprimer/index1.html[52]. Hotstar Taq polymerase (Qiagen, West Sussex, UK) was used for 45 PCR cycles to amplify converted gDNA samples. One to three μl of crude PCR product was ligated into pGEM^®^-T ^® ^Vector System (Promega) as per manufacturer's instructions. Ligations were then incubated at 4°C with JM109 high efficiency competent bacterial cells (Promega) for 30 minutes. The bacterial cells were then heat shocked at 42°C for 45 seconds in a pre-heated water bath and immediately returned on ice for 2 minutes. White colonies were selected for sequencing and resuspended in 100 μl of LB-broth. The resuspended colonies were incubated at 37°C for 1 to 2 hours. Two μl of each colony was amplified by standard PCR reaction with M13 forward and reverse primers or the specific primers designed for the CpG island of interest. Sequences were analysed to determine bisulphite conversion of CpG sites using Bisulphite Sequencing DNA Methylation Analysis (BISMA) webtool http://biochem.jacobs-university.de/BDPC/BISMA/index.php[53].

## Authors' contributions

Conceived and designed the experiments: CD, GS, GEM, ID. Performed the experiments: CD, MSF. Analysed the data: CD, MER, GS, IMS, TC, ST, GEM, ID. Provided reagents and materials: SAA, SC, PS, DK, PD, ETD, GEM, ID. Drafted the paper: CD, MER, GS. Reviewed the paper: PS, ST, GEM, ID. All authors read and approved the final manuscript.

## Supplementary Material

Additional file 1 **Supplemental Table S1: List of SNPs and genes tested on Sequenom platform.** Supplemental Table S2: List of SNPs and genes tested on Illumina platform. Supplemental Table S3: SNPs with an average intensity > 11.25 units Click here for file

Additional file 2 **Figure showing preferential allelic expression of *ACSS2 *on the Sequenom platform**. Averaged allelic ratios for heterozygous gDNA and cDNA were plotted. The higher C/T ratio in cDNA shows preferential C allele expression (t-test p value = 0.0075). Click here for file

Additional file 3 **Figure confirming imprinting of *ZNF331 *in human term placenta by Sanger Sequencing**. Sequences (top for rs8100247 (exon 1, 5'UTR) and bottom for rs8109631 (exon 7, CDS)) of informative term placenta samples in gDNA and cDNA with corresponding genotyping data for the father and the mother. Complete imprinting is visible for the exon 1 SNP, while partial imprinting is present for the exon 7 SNP suggesting an isoform specific imprinting. It is the maternal allele that is (more) expressed. Click here for file

Additional file 4 **Figure showing statistically significant genes exhibiting preferential ASE on the Illumina array**. ASE for *SQSTM1*, *UBE2V1 *and *XRRA1 *is evident while the effect for *CAST *and *MAN2C1 *is more subtle. Click here for file
