# Supplementary figures and images for: High-throughput analysis of candidate imprinted genes and allele-specific gene expression in the human term placenta
Source: BMC Genet. 2010 Apr 19;11:25. doi: 10.1186/1471-2156-11-25 (PMC2871261; doi:10.1186/1471-2156-11-25)

## Slide 1
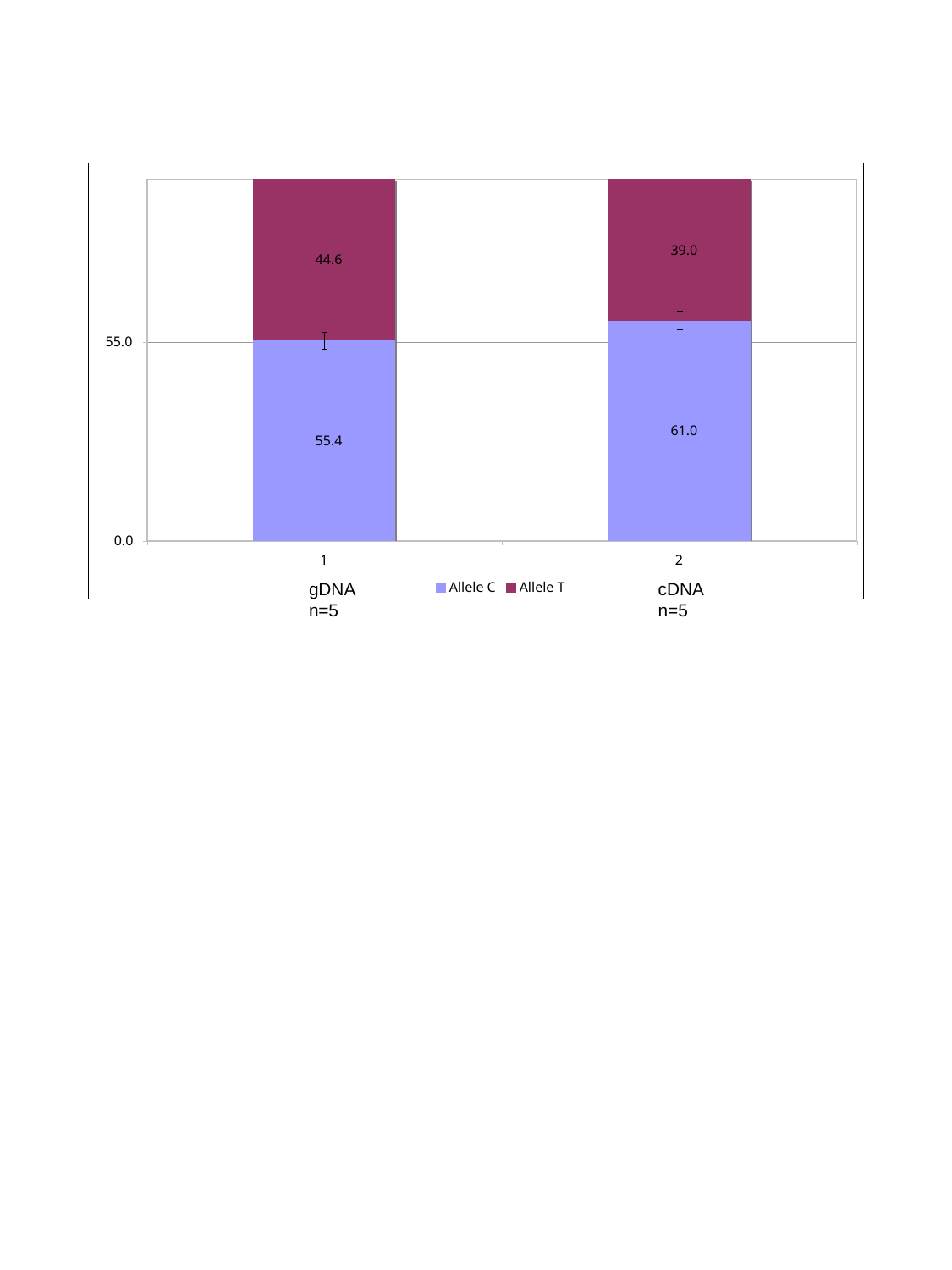

gDNA
n=5
cDNA
n=5

Supplement: Additional file 2 — Figure showing preferential allelic expression of ACSS2 on the Sequenom platform. Averaged allelic ratios for heterozygous gDNA and cDNA were plotted. The higher C/T ratio in cDNA shows preferential C allele expression (t-test p value = 0.0075). [file 1471-2156-11-25-S2.PPT]
