# Supplementary material for: High-throughput analysis of candidate imprinted genes and allele-specific gene expression in the human term placenta
Source: BMC Genet. 2010 Apr 19;11:25. doi: 10.1186/1471-2156-11-25 (PMC2871261; doi:10.1186/1471-2156-11-25)
Supplement: Additional file 3 — Figure confirming imprinting of ZNF331 in human term placenta by Sanger Sequencing. Sequences (top for rs8100247 (exon 1, 5'UTR) and bottom for rs8109631 (exon 7, CDS)) of informative term placenta samples in gDNA and cDNA with corresponding genotyping data for the father and the mother. Complete imprinting is visible for the exon 1 SNP, while partial imprinting is present for the exon 7 SNP suggesting an isoform specific imprinting. It is the maternal allele that is (more) expressed. [file 1471-2156-11-25-S3.PDF]

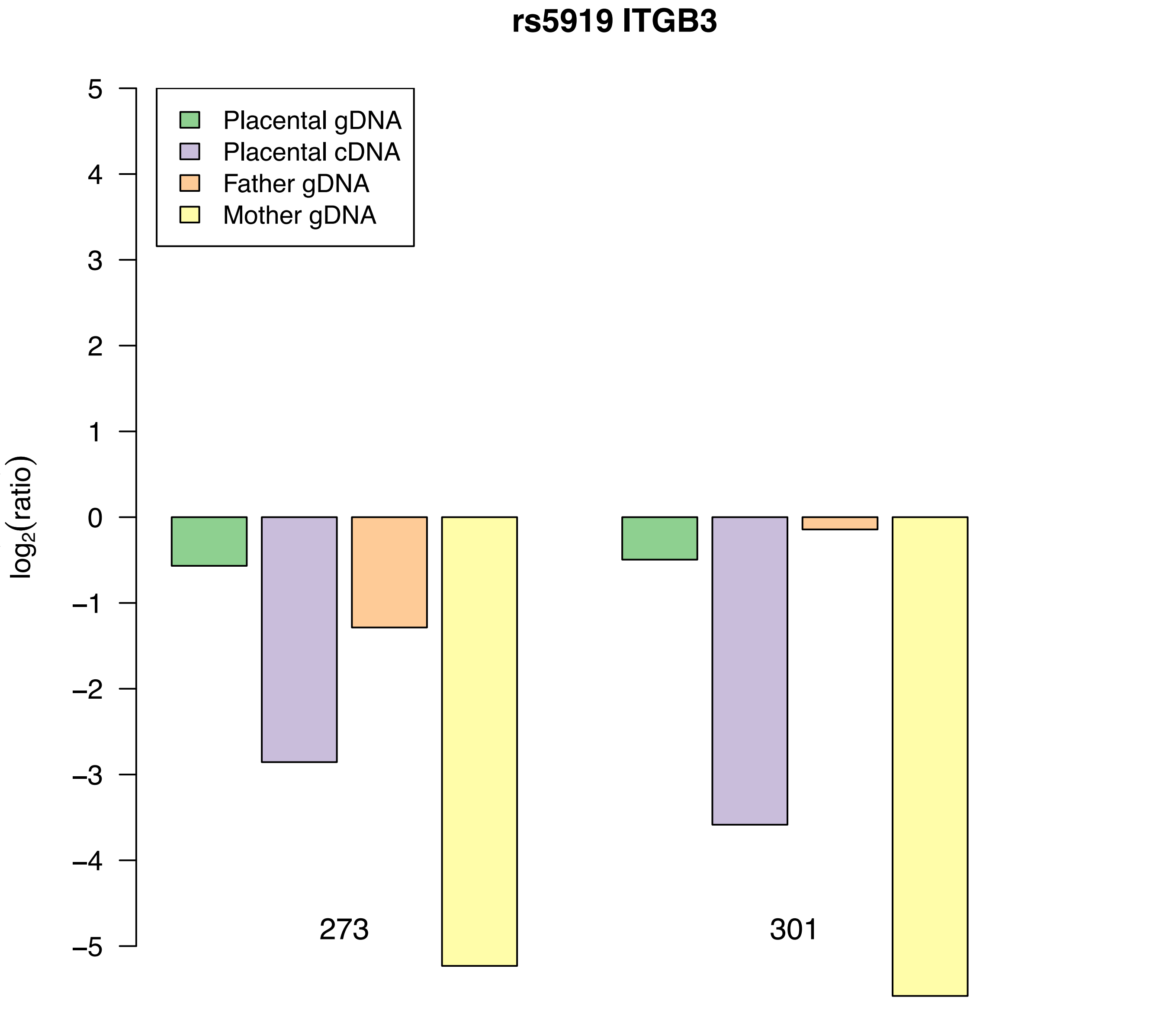

rs5919 ITGB3
Placental gDNA
Placental cDNA
Father gDNA
Mother gDNA
log2(ratio)
5
4
3
2
1
0
−1
−2
−3
−4
−5
273
301

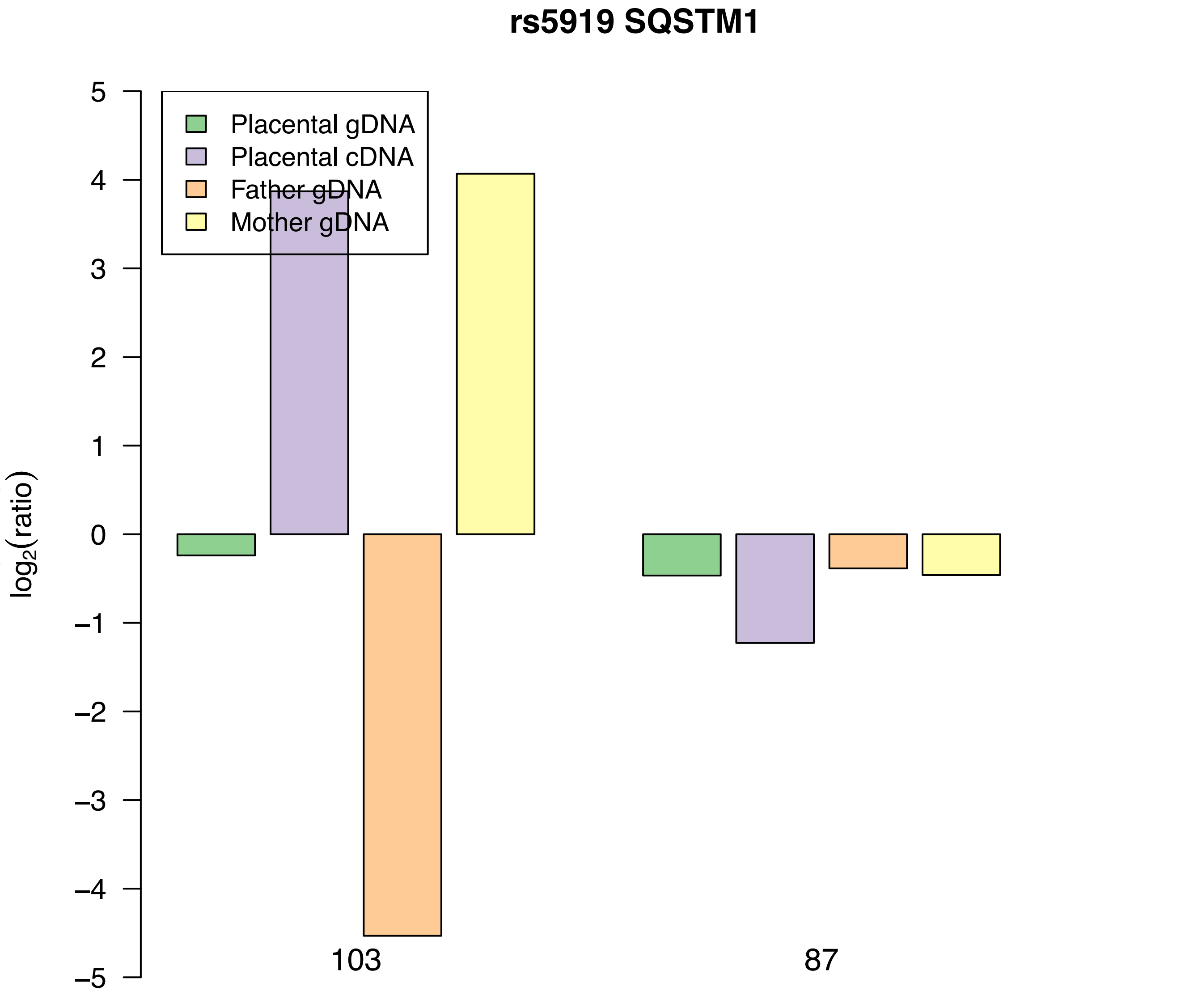
rs5919 SQSTM1
Placental gDNA
Placental cDNA
Father gDNA
Mother gDNA
log2(ratio)
5
4
3
2
1
0
−1
−2
−3
−4
−5
103
87

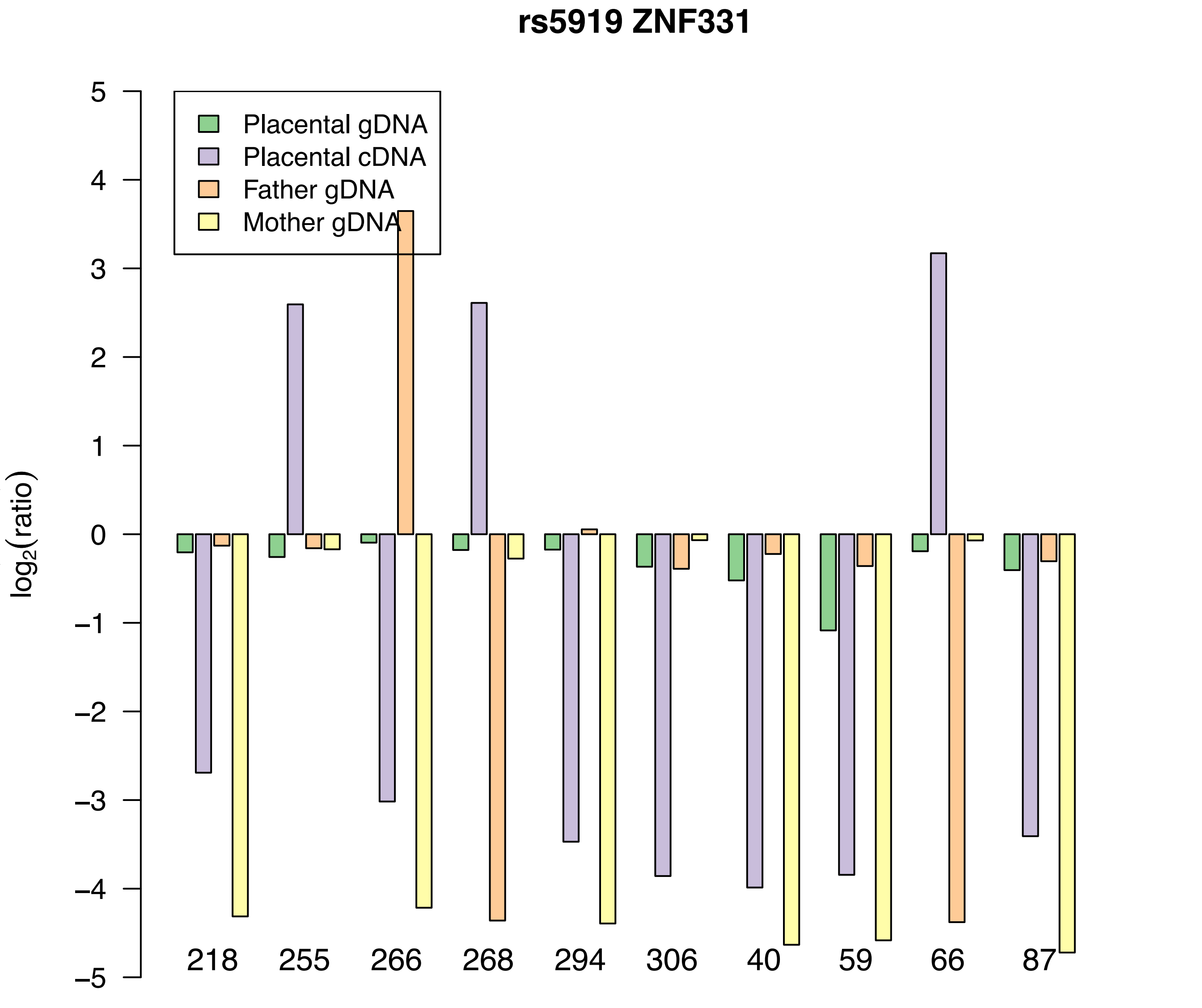
rs5919 ZNF331
log2(ratio)
Placental gDNA
Placental cDNA
Father gDNA
Mother gDNA
5
4
3
2
1
0
−1
−2
−3
−4
−5
218
255
266
268
294
306
40
59
66
87

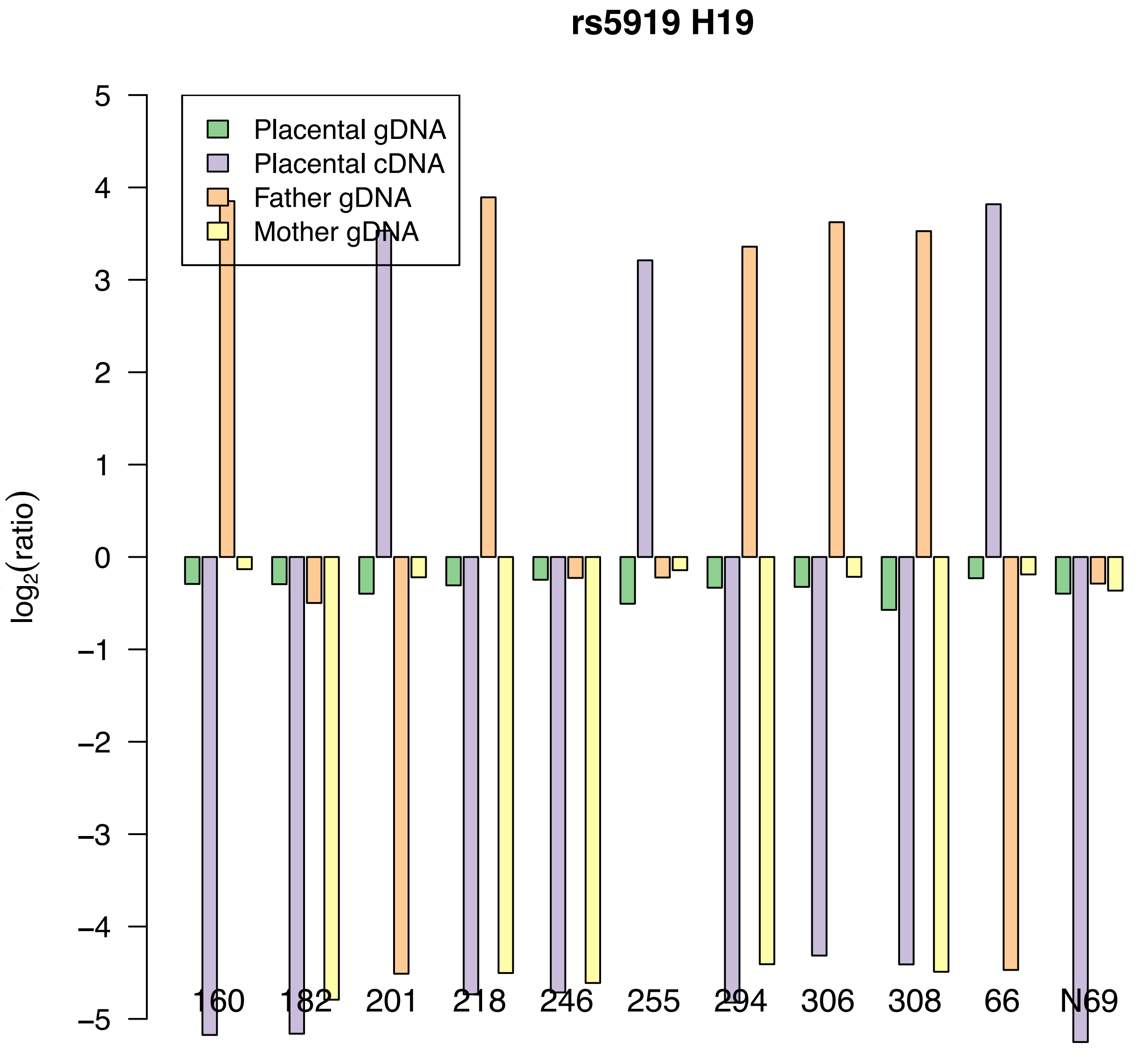

rs5919 H19
5
4
3
2
1
0
−1
−2
−3
−4
−5
log2(ratio)
Placental gDNA
Placental cDNA
Father gDNA
Mother gDNA
160
182
201
218
246
255
294
306
308
66
N69

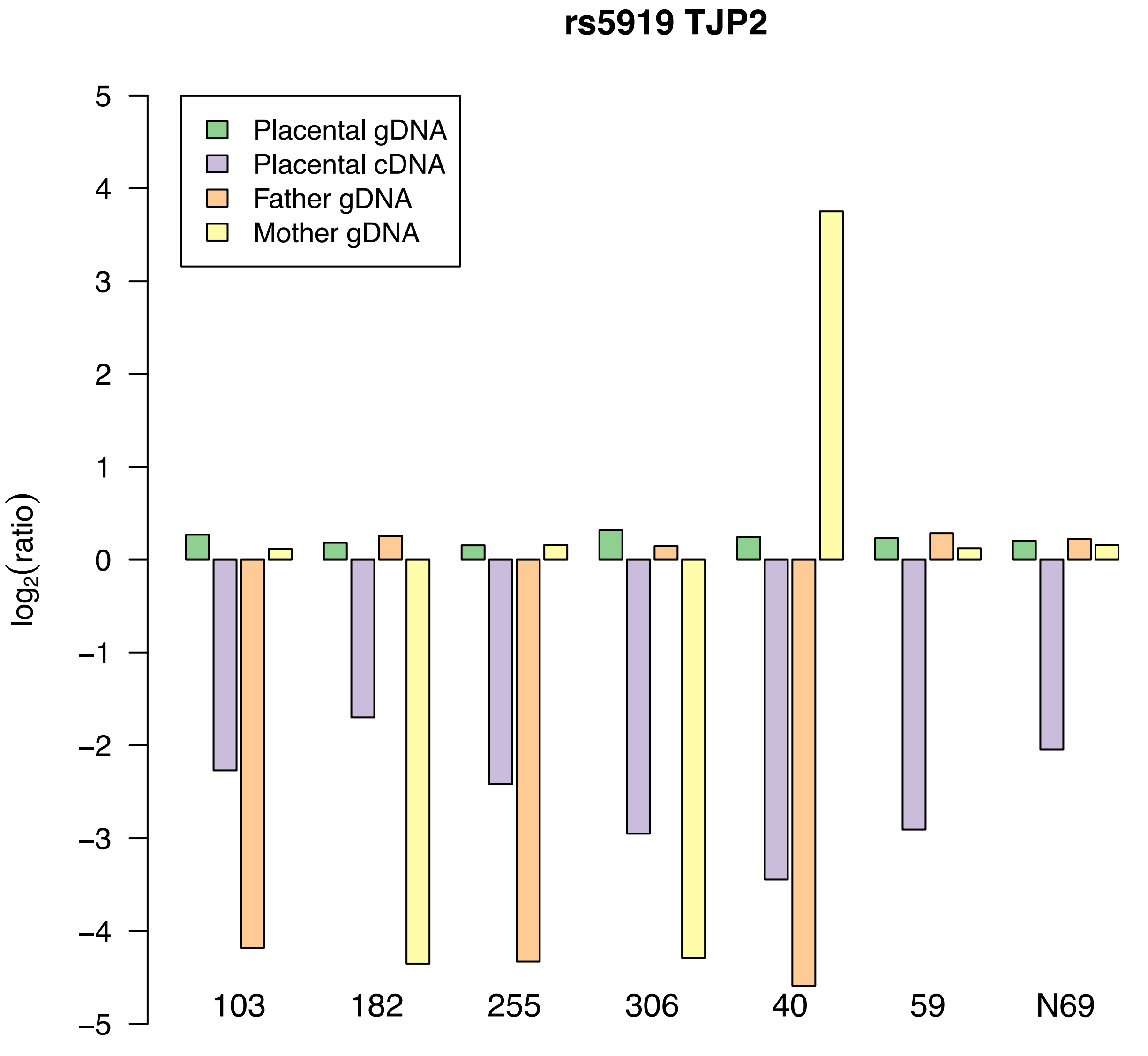
rs5919 TJP2
Placental gDNA
Placental cDNA
Father gDNA
Mother gDNA
log2(ratio)
5
4
3
2
1
0
−1
−2
−3
−4
−5
103
182
255
306
40
59
N69

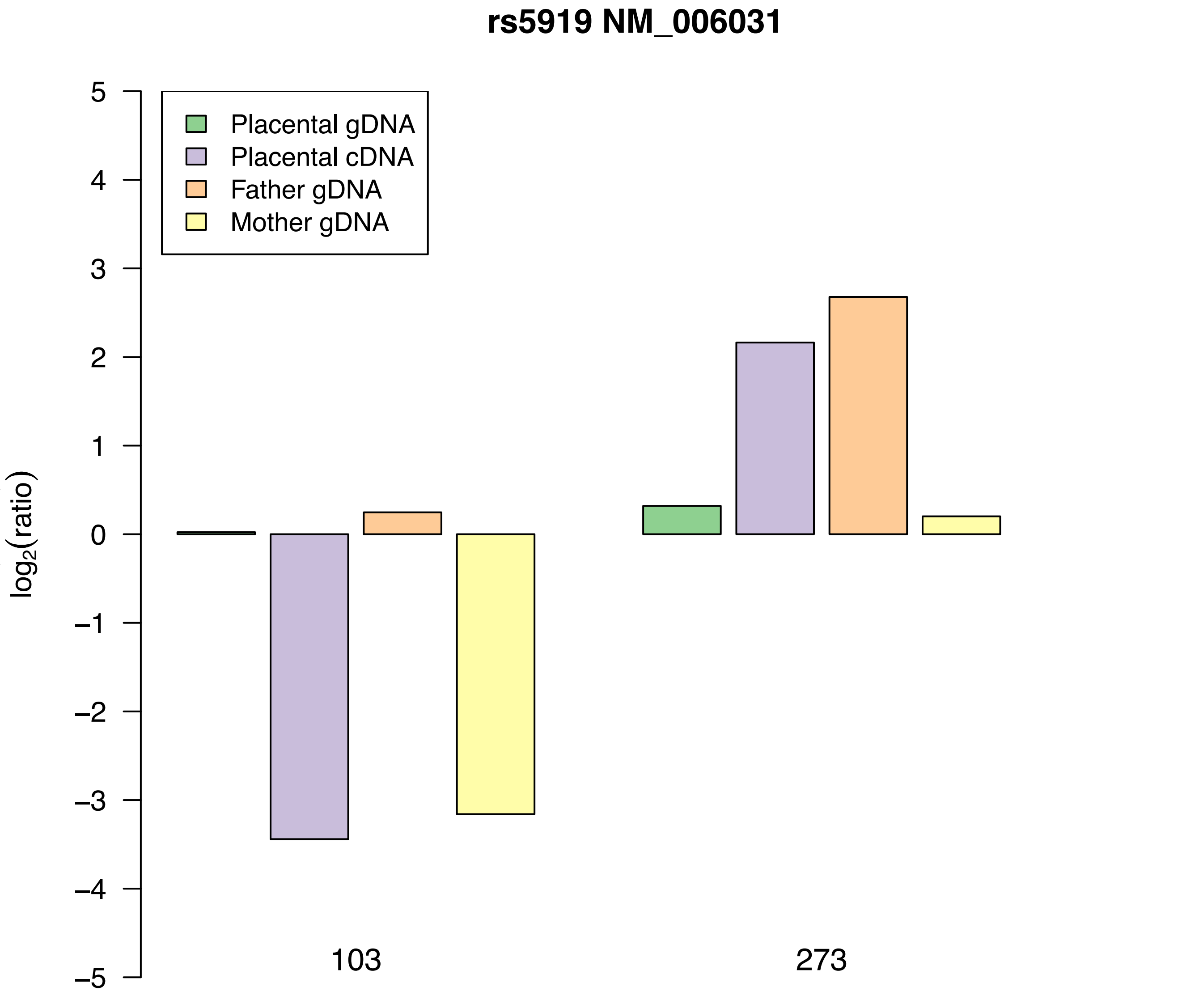
rs5919 NM_006031
Placental gDNA
Placental cDNA
Father gDNA
Mother gDNA
5
4
3
2
1
0
−1
−2
−3
−4
−5
log_2(ratio)
103
273

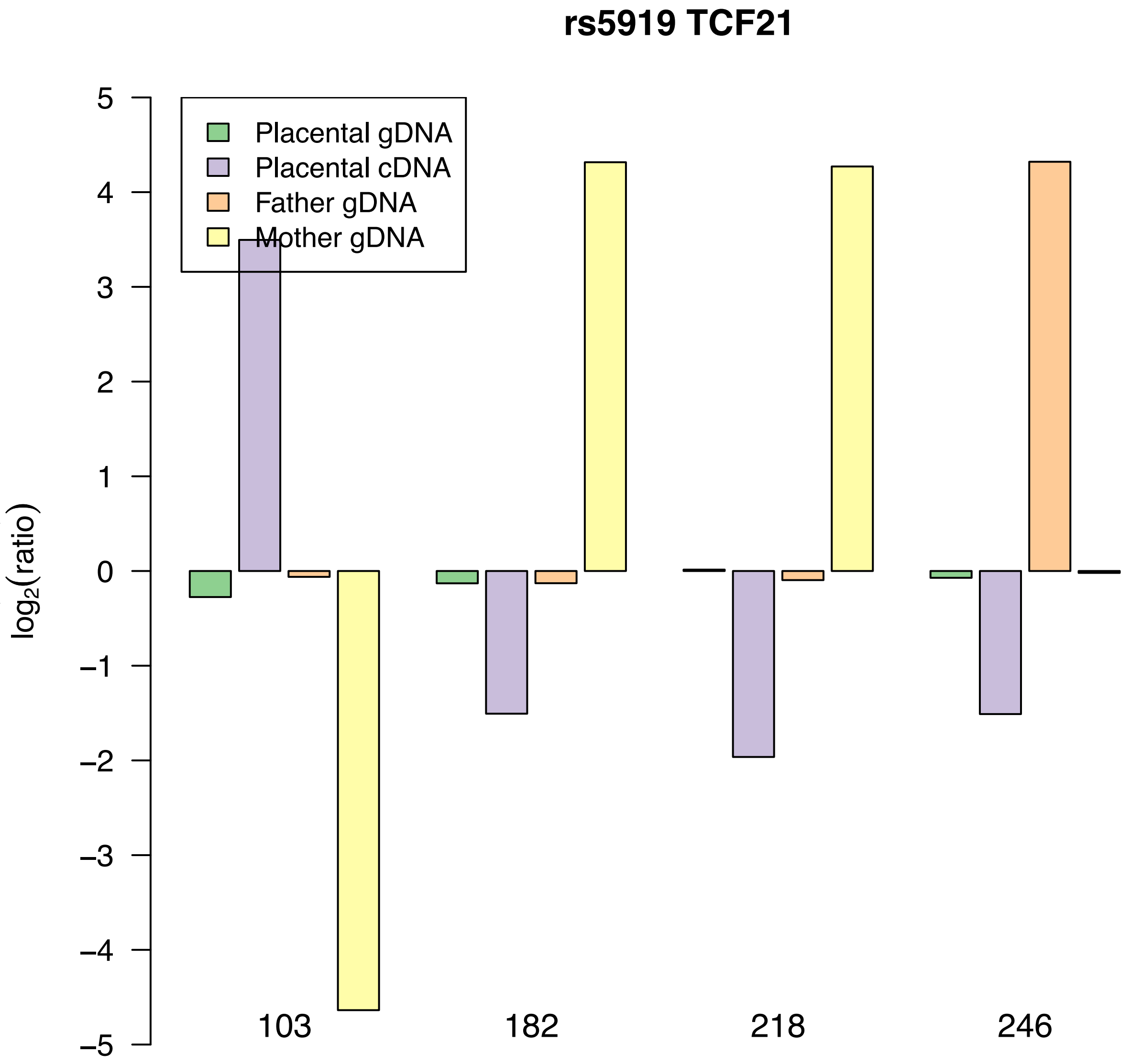
rs5919 TCF21
Placental gDNA
Placental cDNA
Father gDNA
Mother gDNA
log2(ratio)
5
4
3
2
1
0
−1
−2
−3
−4
−5
103
182
218
246

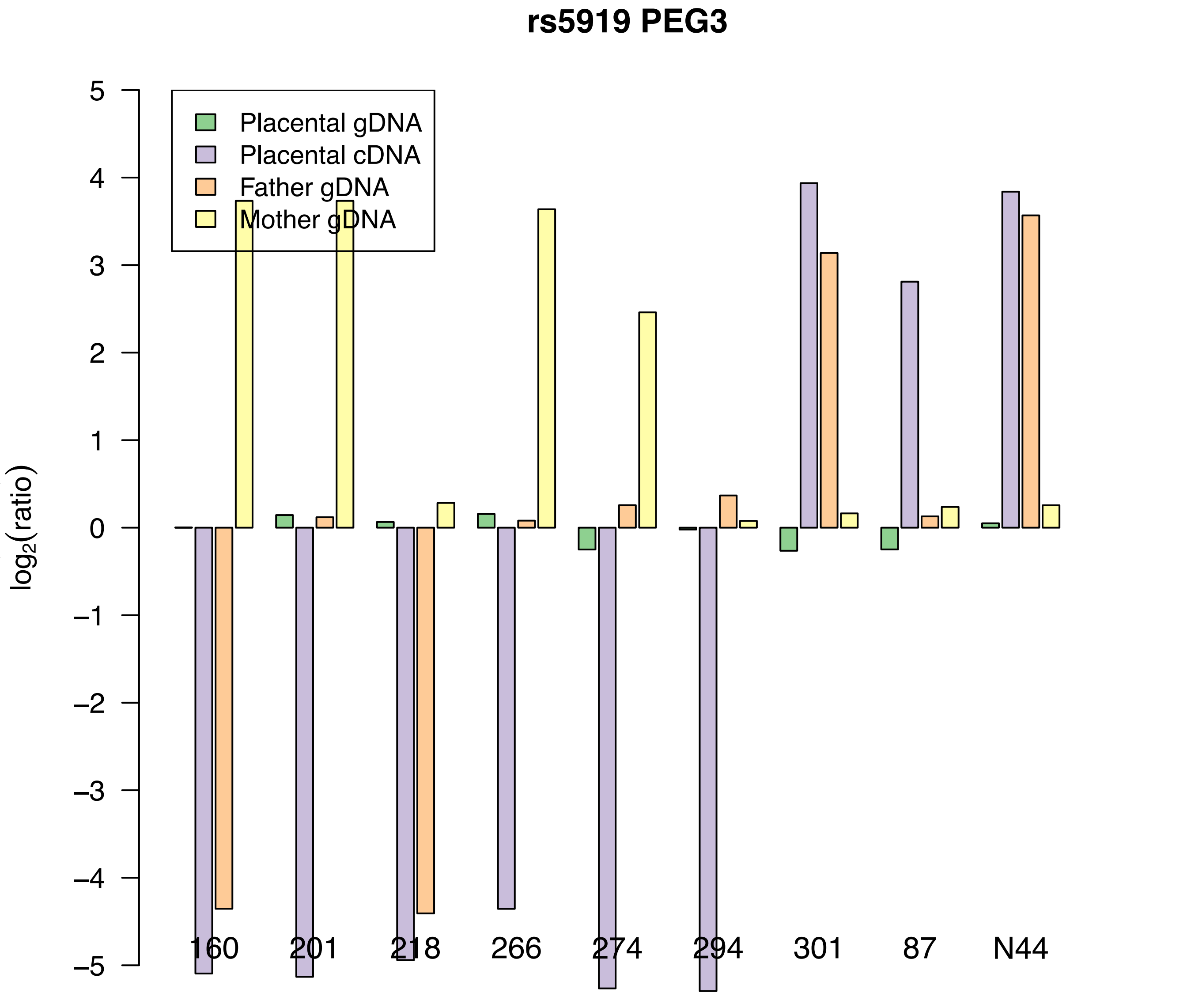
rs5919 PEG3
Placental gDNA
Placental cDNA
Father gDNA
Mother gDNA
log2(ratio)
5
4
3
2
1
0
−1
−2
−3
−4
−5
160
201
218
266
274
294
301
87
N44

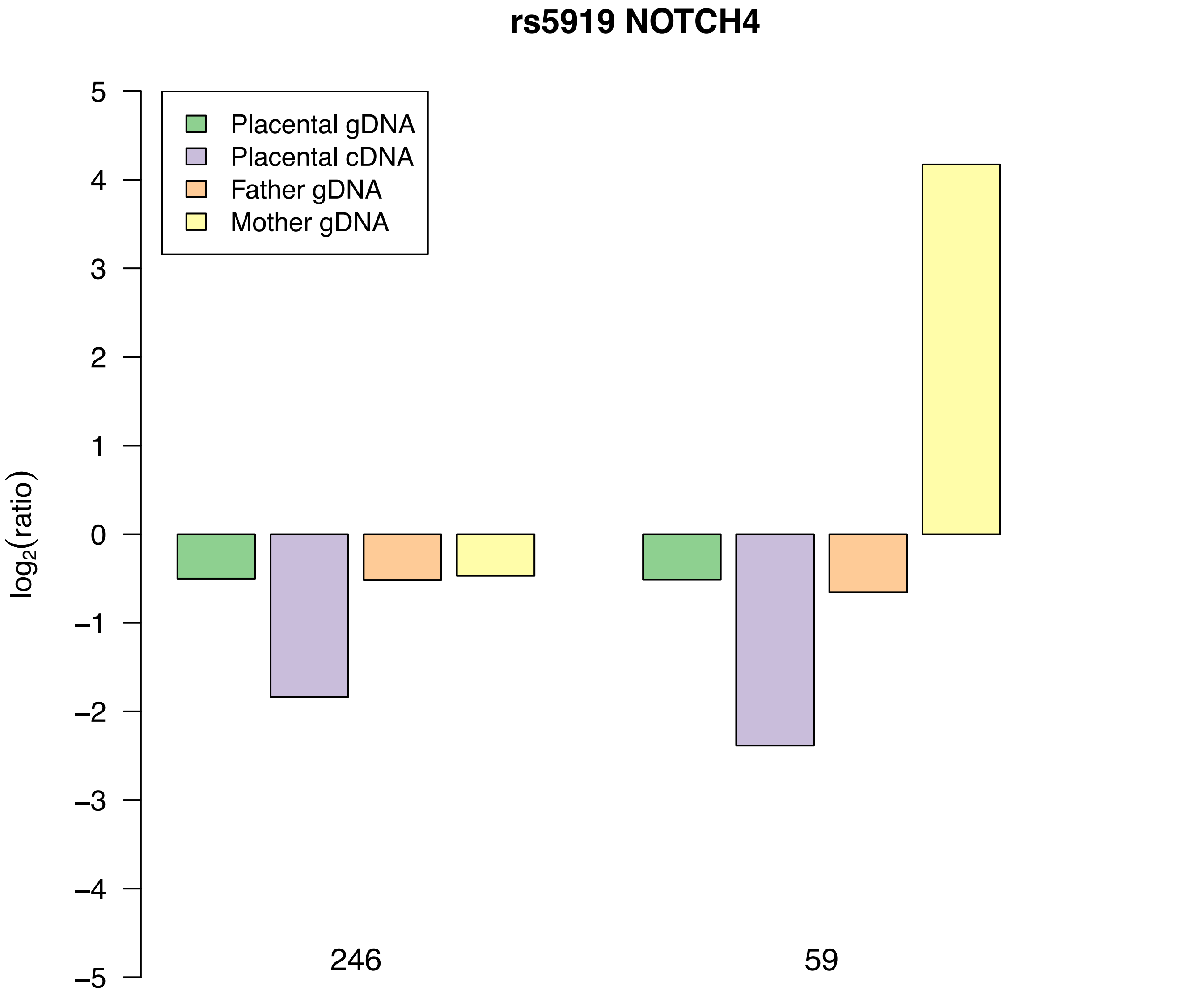
rs5919 NOTCH4
Placental gDNA
Placental cDNA
Father gDNA
Mother gDNA
log2(ratio)
5
4
3
2
1
0
−1
−2
−3
−4
−5
246
59

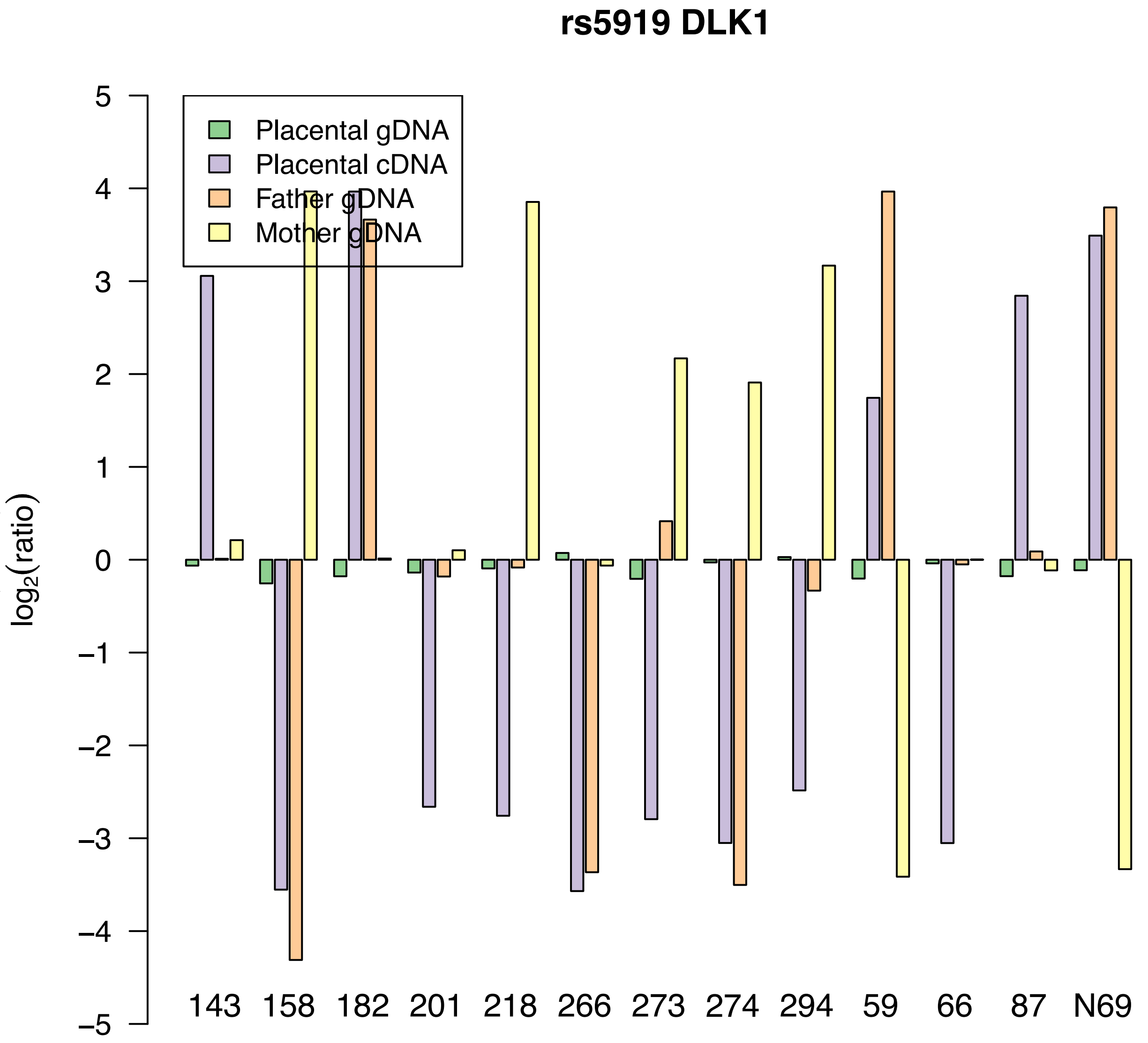
rs5919 DLK1
Placental gDNA
Placental cDNA
Father gDNA
Mother gDNA
log2(ratio)
5
4
3
2
1
0
−1
−2
−3
−4
−5
143
158
182
201
218
266
273
274
294
59
66
87
N69

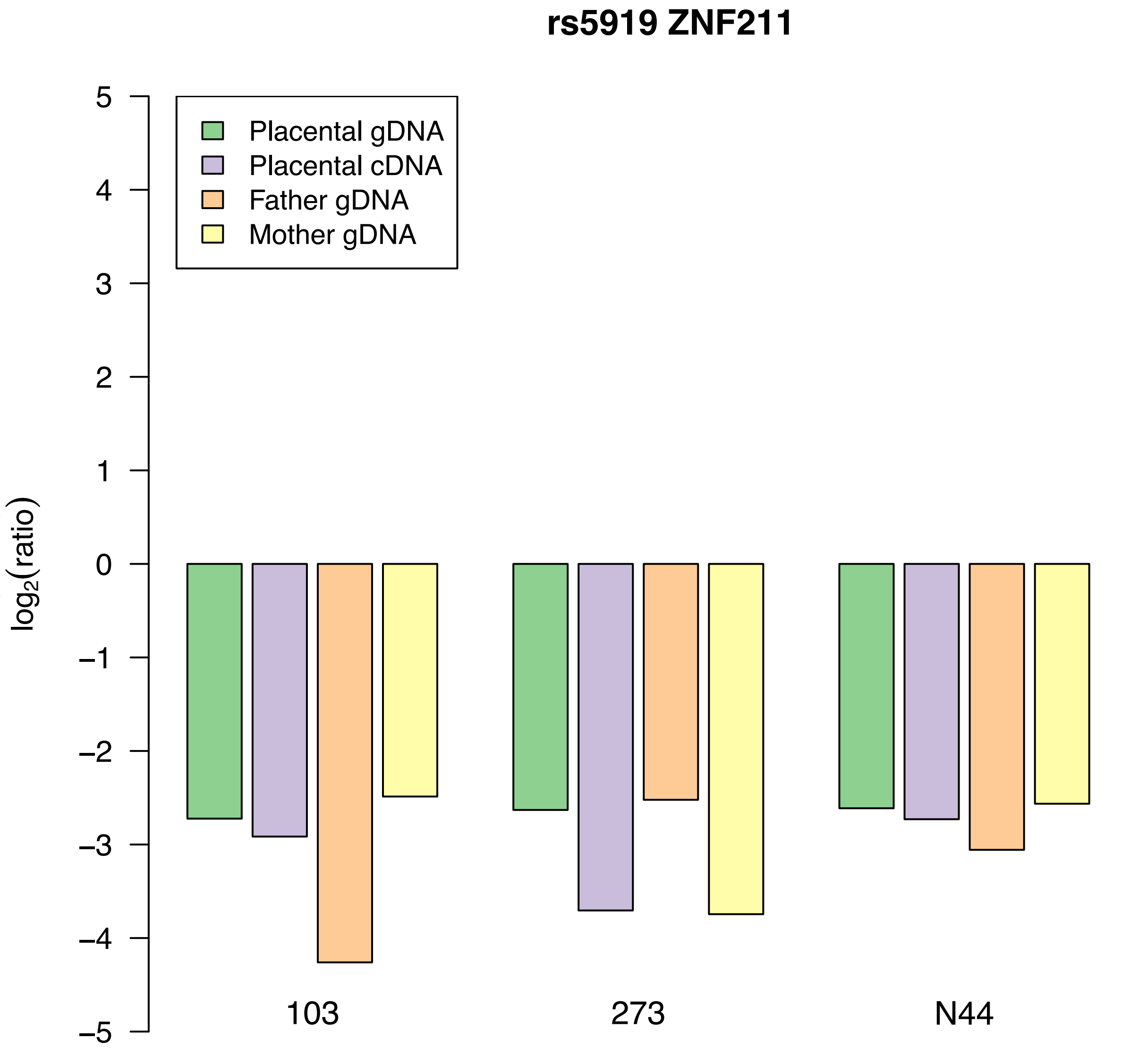
rs5919 ZNF211
log2(ratio)
Placental gDNA
Placental cDNA
Father gDNA
Mother gDNA
5
4
3
2
1
0
−1
−2
−3
−4
−5
103
273
N44

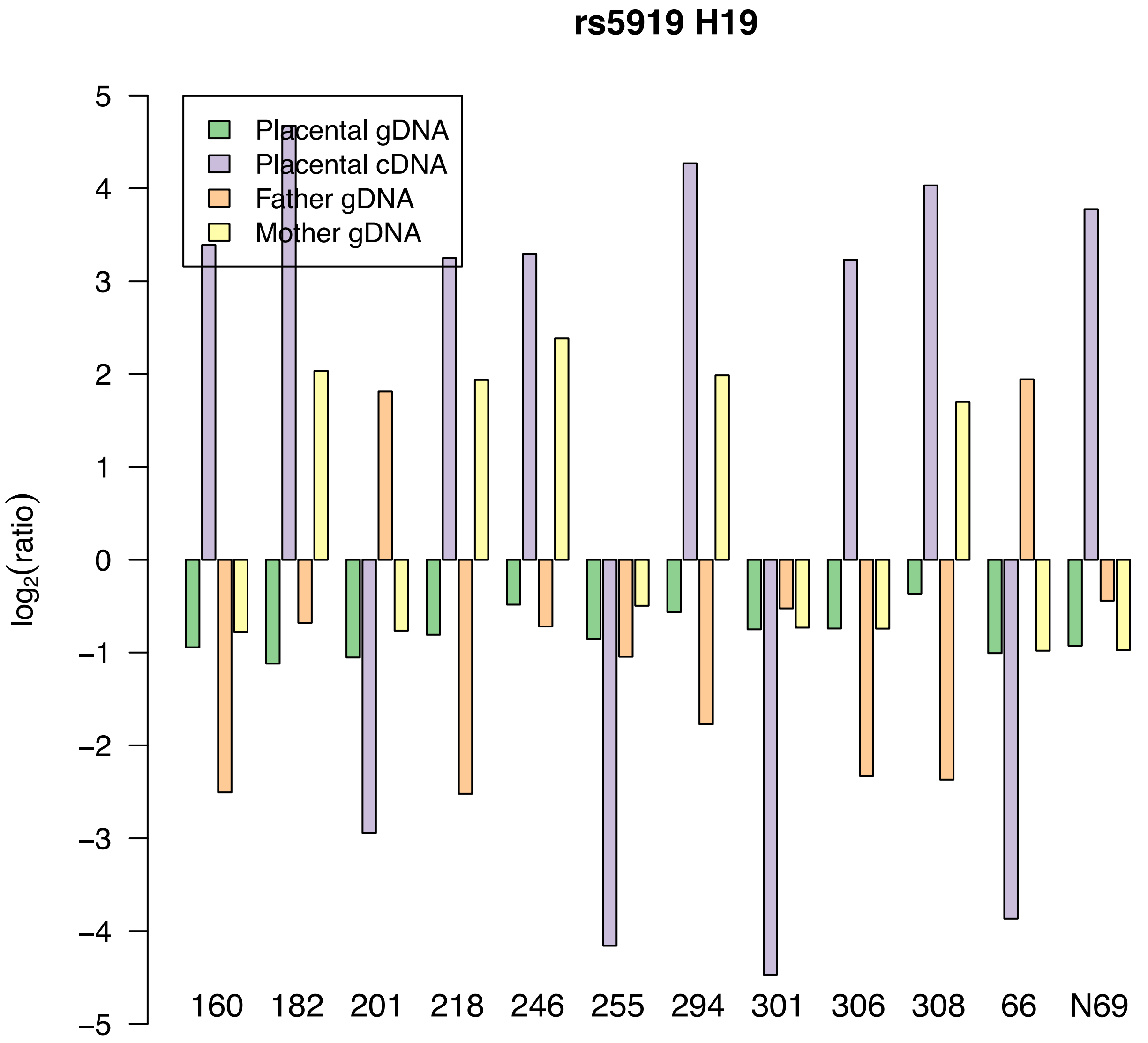
rs5919 H19
Placental gDNA
Placental cDNA
Father gDNA
Mother gDNA
log2(ratio)
5
4
3
2
1
0
−1
−2
−3
−4
−5
160
182
201
218
246
255
294
301
306
308
66
N69

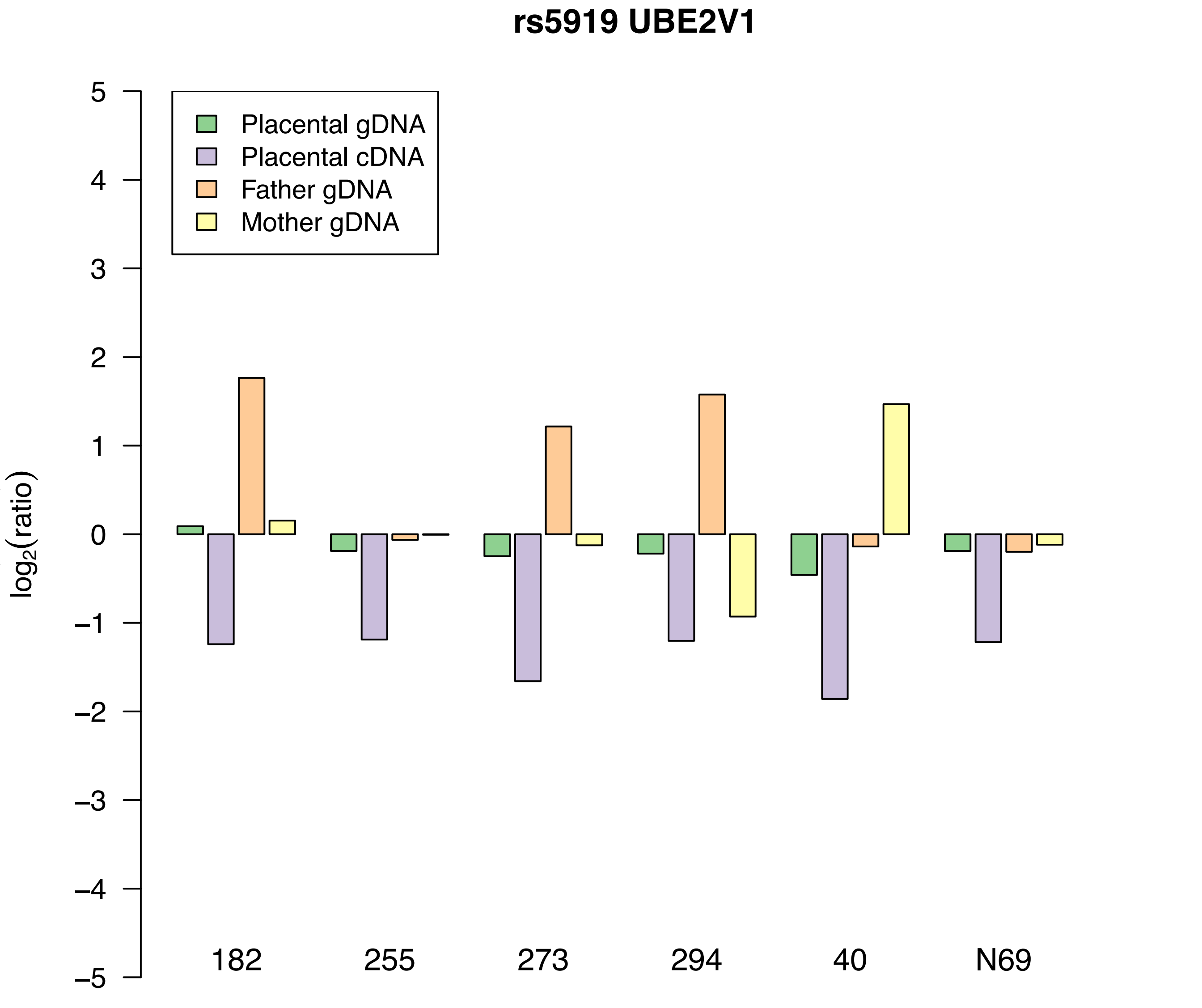
rs5919 UBE2V1
Placental gDNA
Placental cDNA
Father gDNA
Mother gDNA
log2(ratio)
5
4
3
2
1
0
−1
−2
−3
−4
−5
182
255
273
294
40
N69

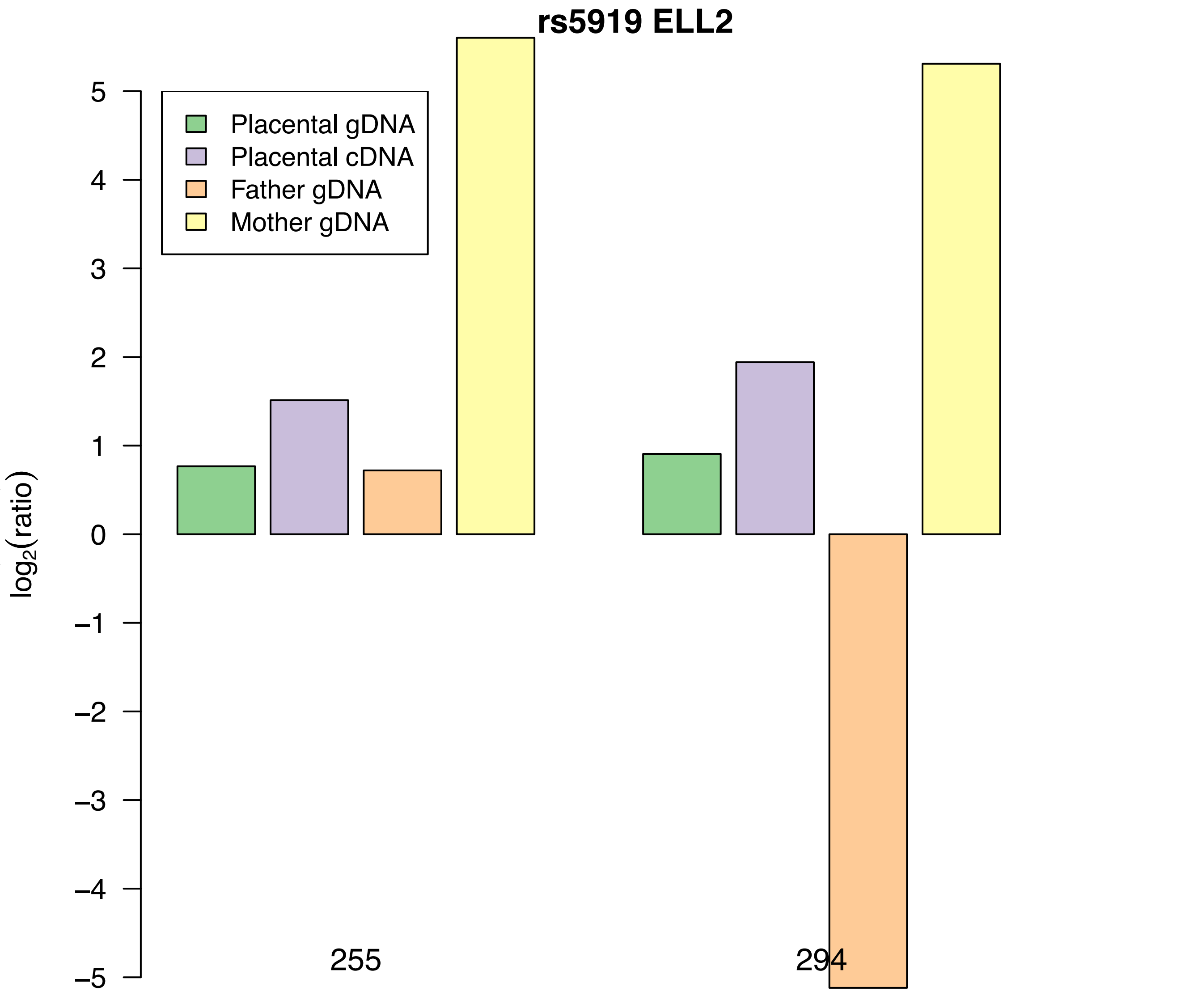
rs5919 ELL2
Placental gDNA
Placental cDNA
Father gDNA
Mother gDNA
log2(ratio)
5
4
3
2
1
0
−1
−2
−3
−4
−5
255
294

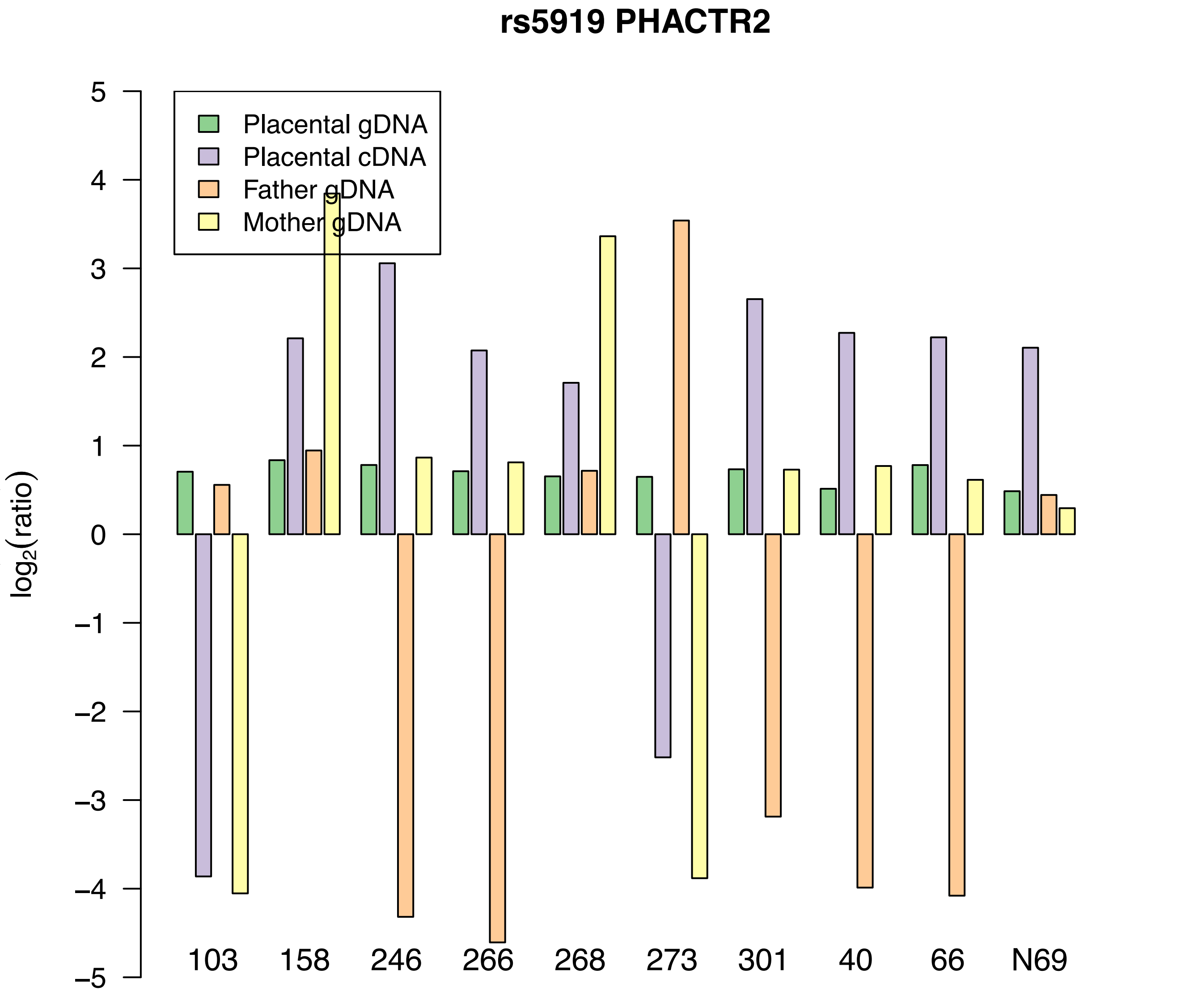
rs5919 PHACTR2
Placental gDNA
Placental cDNA
Father gDNA
Mother gDNA
log2(ratio)
5
4
3
2
1
0
−1
−2
−3
−4
−5
103
158
246
266
268
273
301
40
66
N69

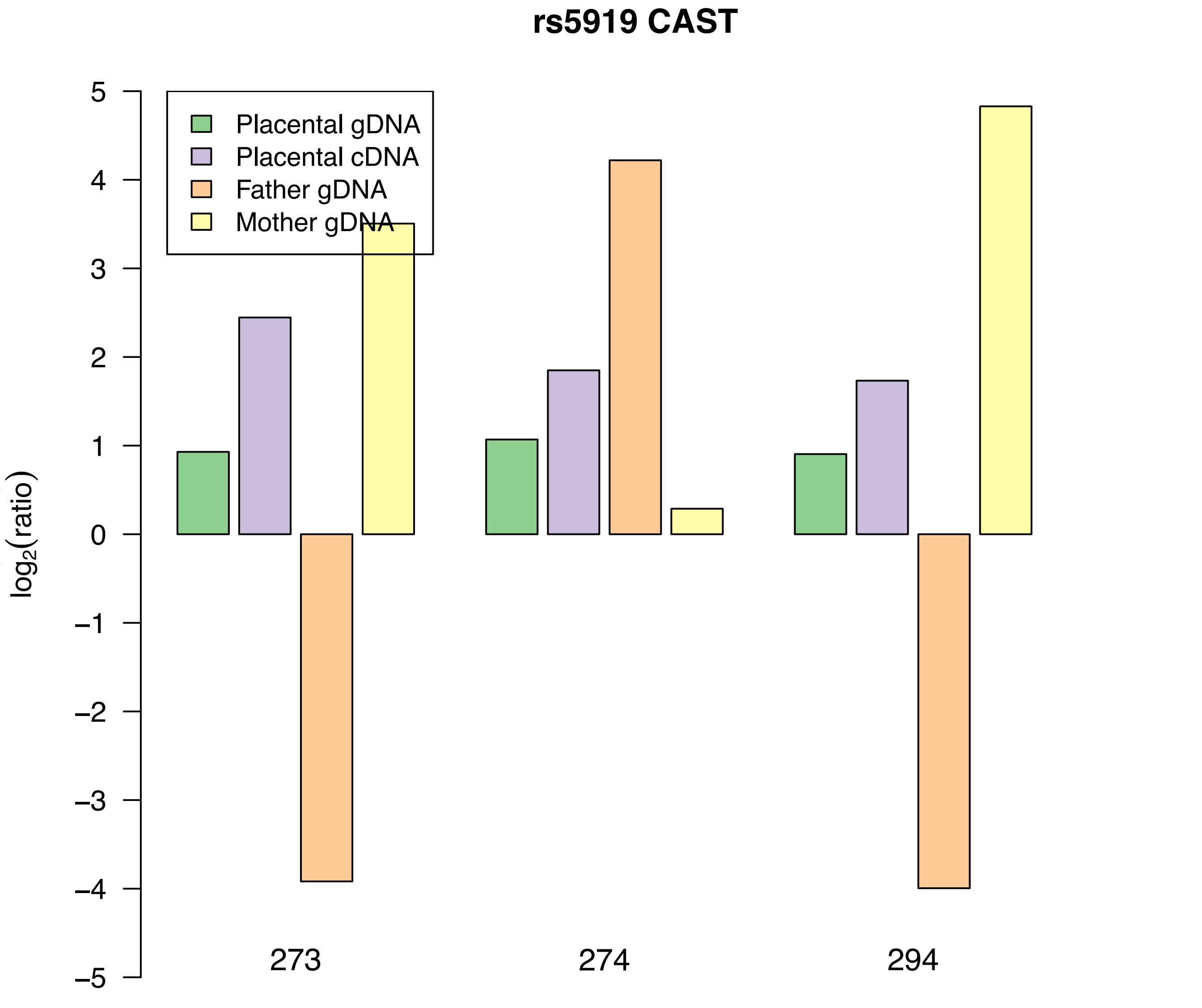

rs5919 CAST
Placental gDNA
Placental cDNA
Father gDNA
Mother gDNA
log2(ratio)
5
4
3
2
1
0
−1
−2
−3
−4
−5
273
274
294

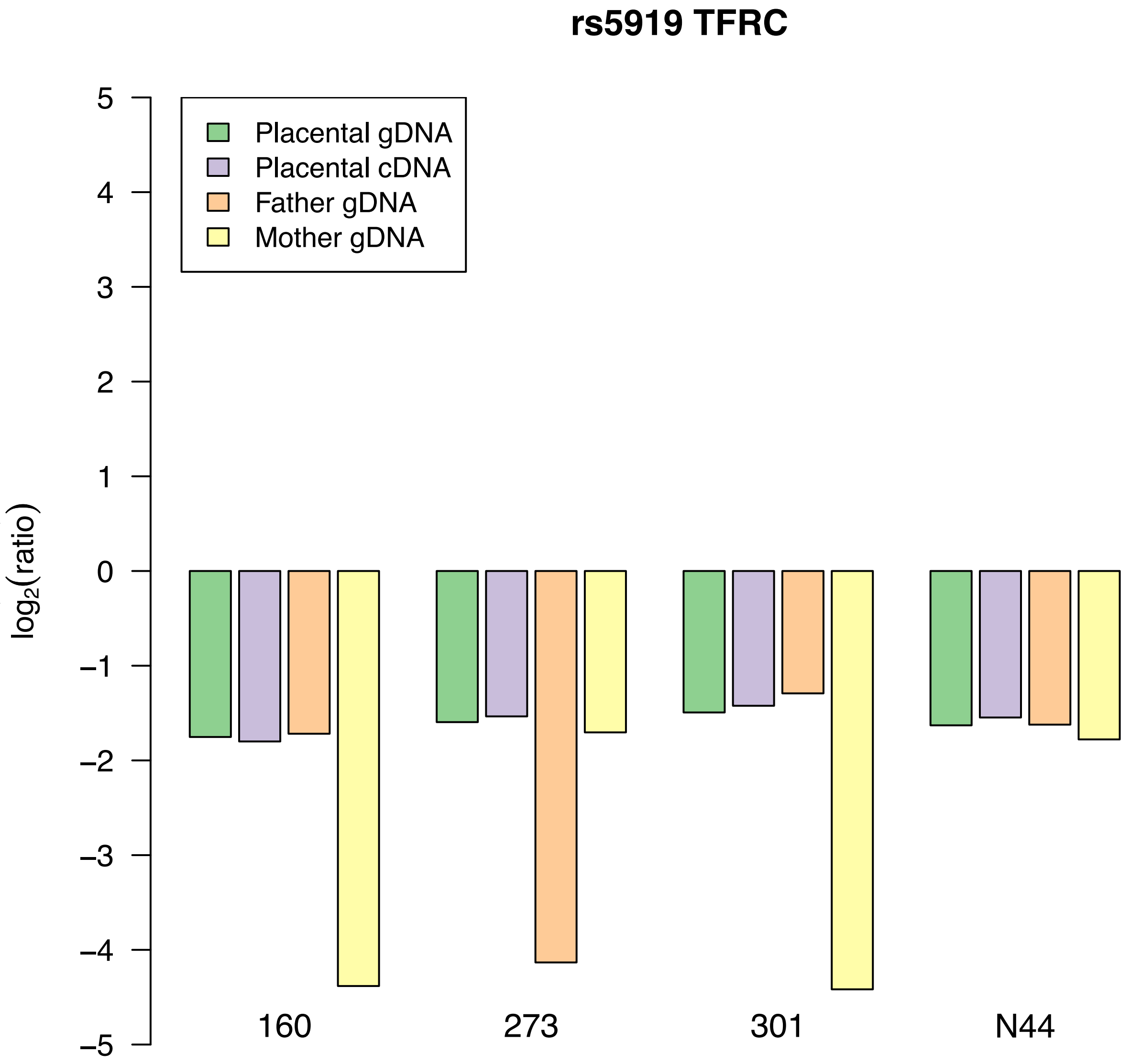

rs5919 TFRC
Placental gDNA
Placental cDNA
Father gDNA
Mother gDNA
log2(ratio)
5
4
3
2
1
0
−1
−2
−3
−4
−5
160
273
301
N44

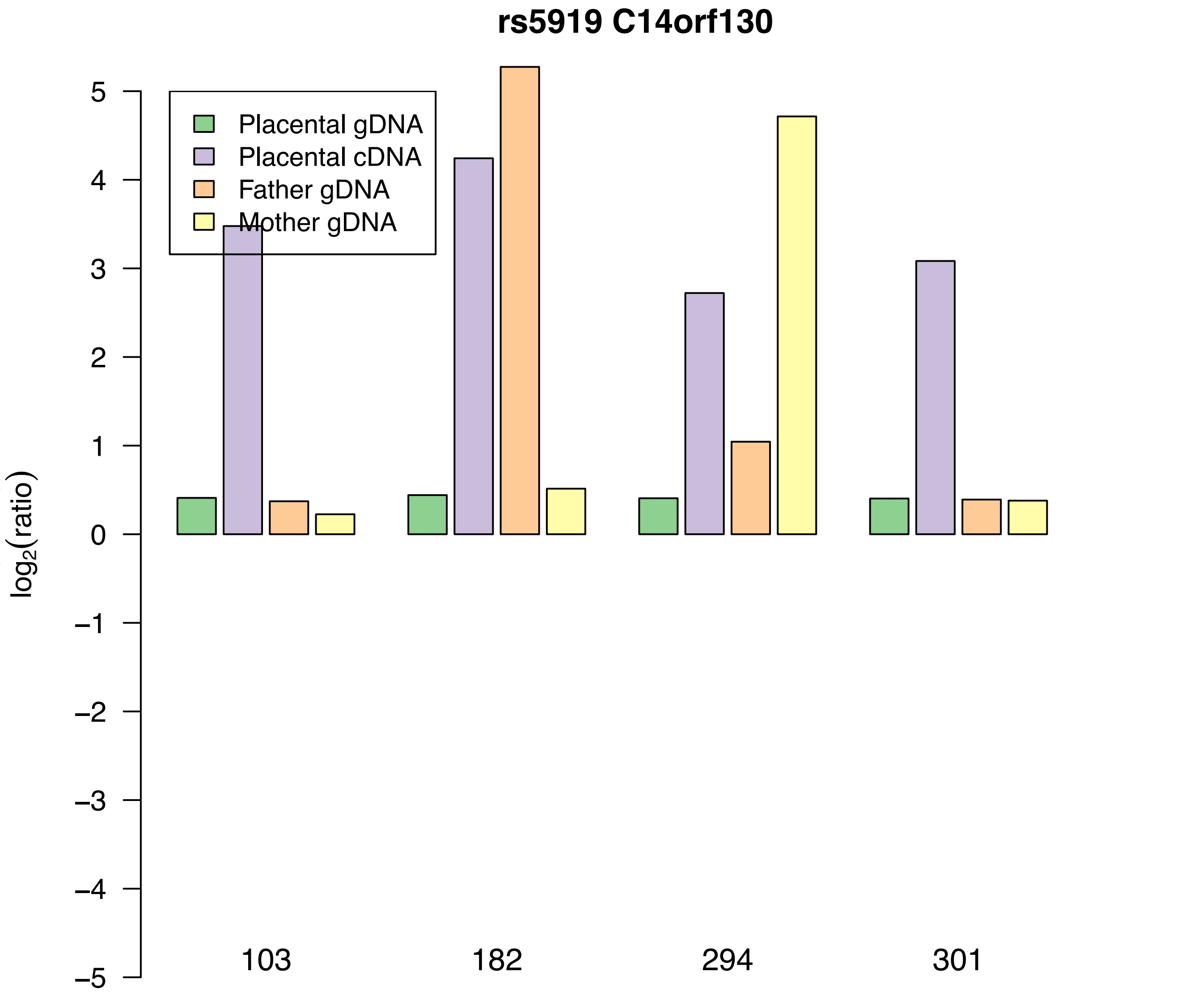
rs5919 C14orf130
Placental gDNA
Placental cDNA
Father gDNA
Mother gDNA
$\log_2(\text{ratio})$
5
4
3
2
1
0
−1
−2
−3
−4
−5
103
182
294
301

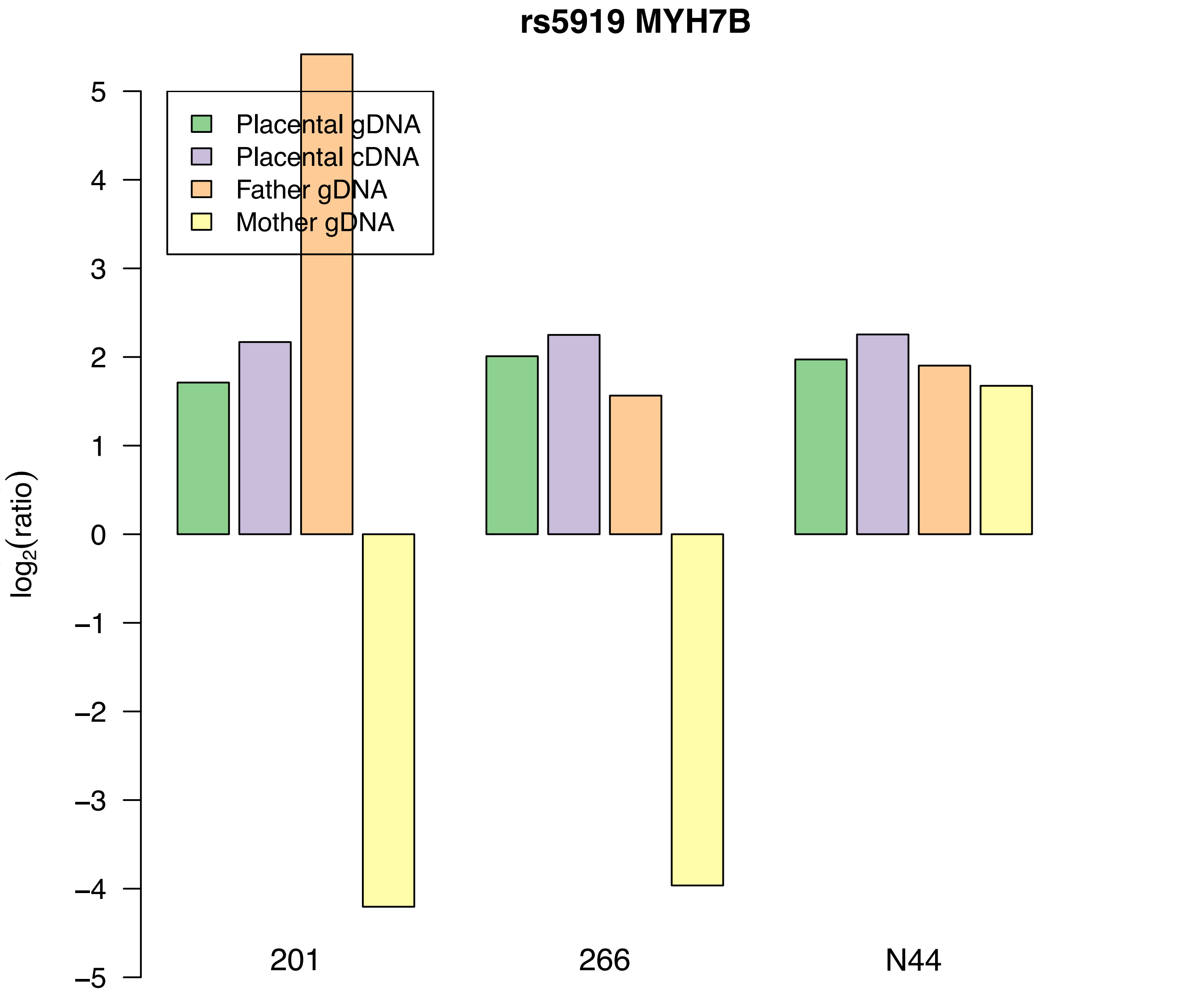
rs5919 MYH7B
Placental gDNA
Placental cDNA
Father gDNA
Mother gDNA
log2(ratio)
5
4
3
2
1
0
−1
−2
−3
−4
−5
201
266
N44

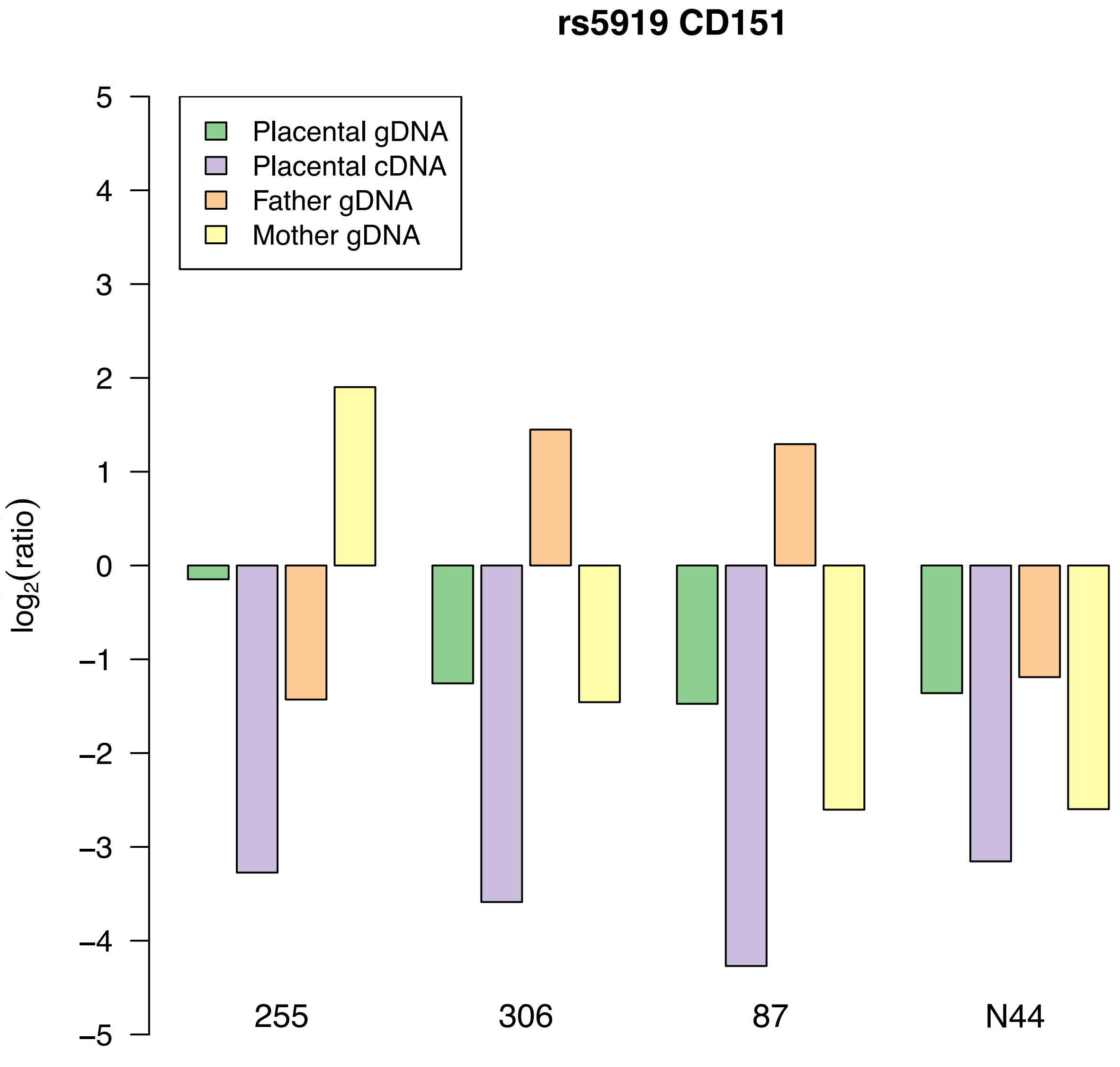

rs5919 CD151
Placental gDNA
Placental cDNA
Father gDNA
Mother gDNA
log2(ratio)
5
4
3
2
1
0
−1
−2
−3
−4
−5
255
306
87
N44

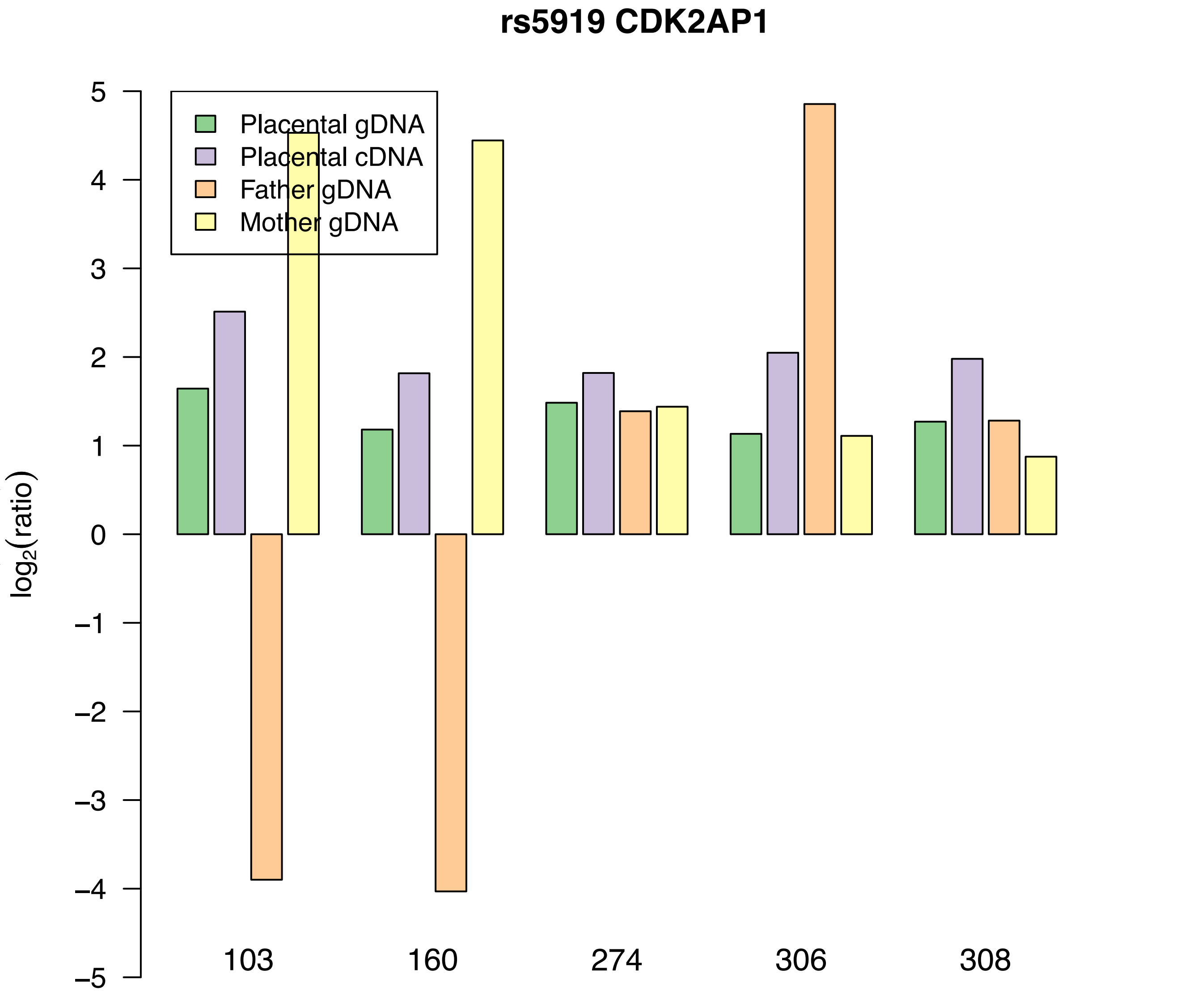
rs5919 CDK2AP1
Placental gDNA
Placental cDNA
Father gDNA
Mother gDNA
log2(ratio)
5
4
3
2
1
0
−1
−2
−3
−4
−5
103
160
274
306
308

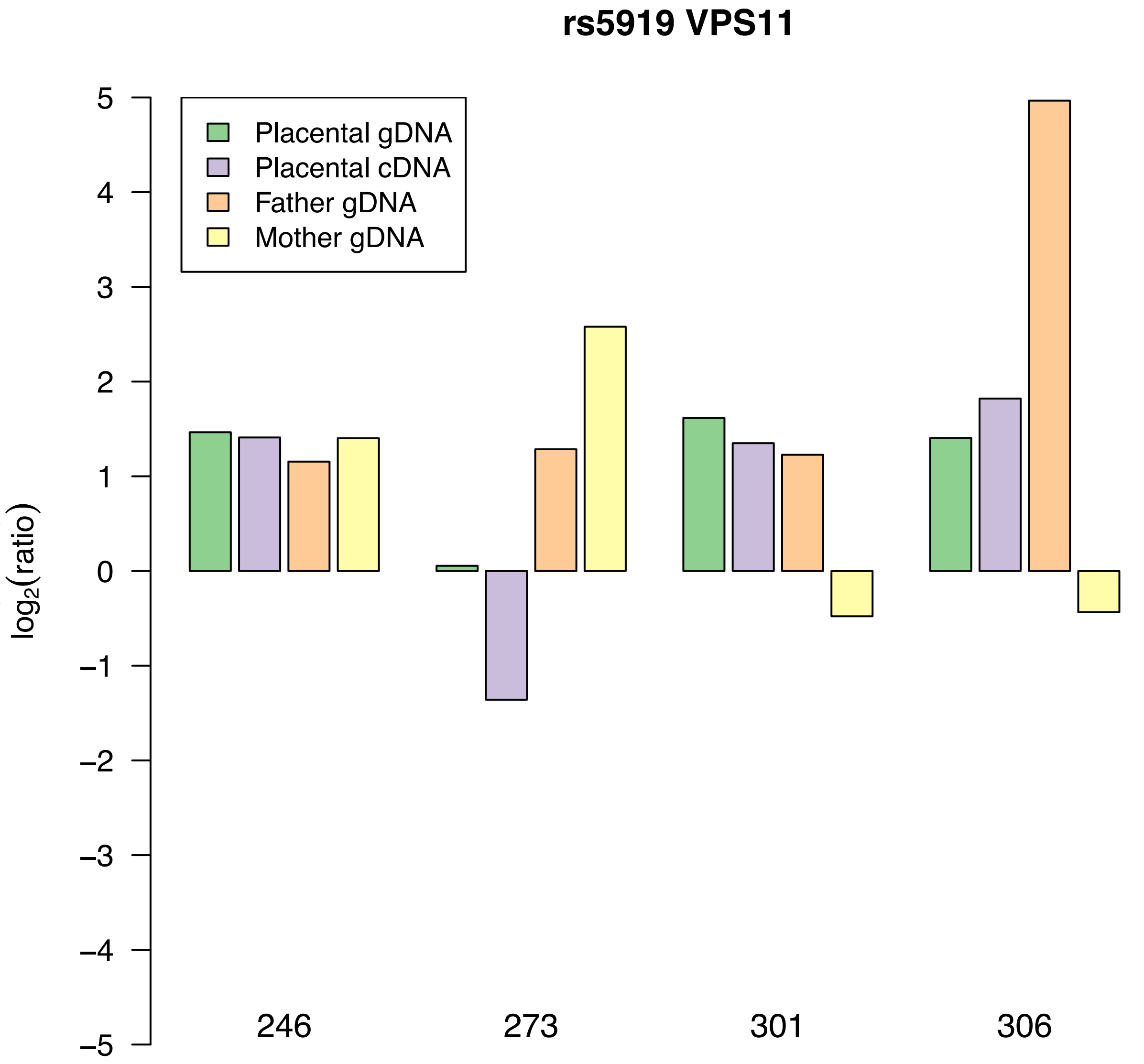

rs5919 VPS11
Placental gDNA
Placental cDNA
Father gDNA
Mother gDNA
5
4
3
2
1
0
−1
−2
−3
−4
−5
$\log_2(\text{ratio})$
246
273
301
306

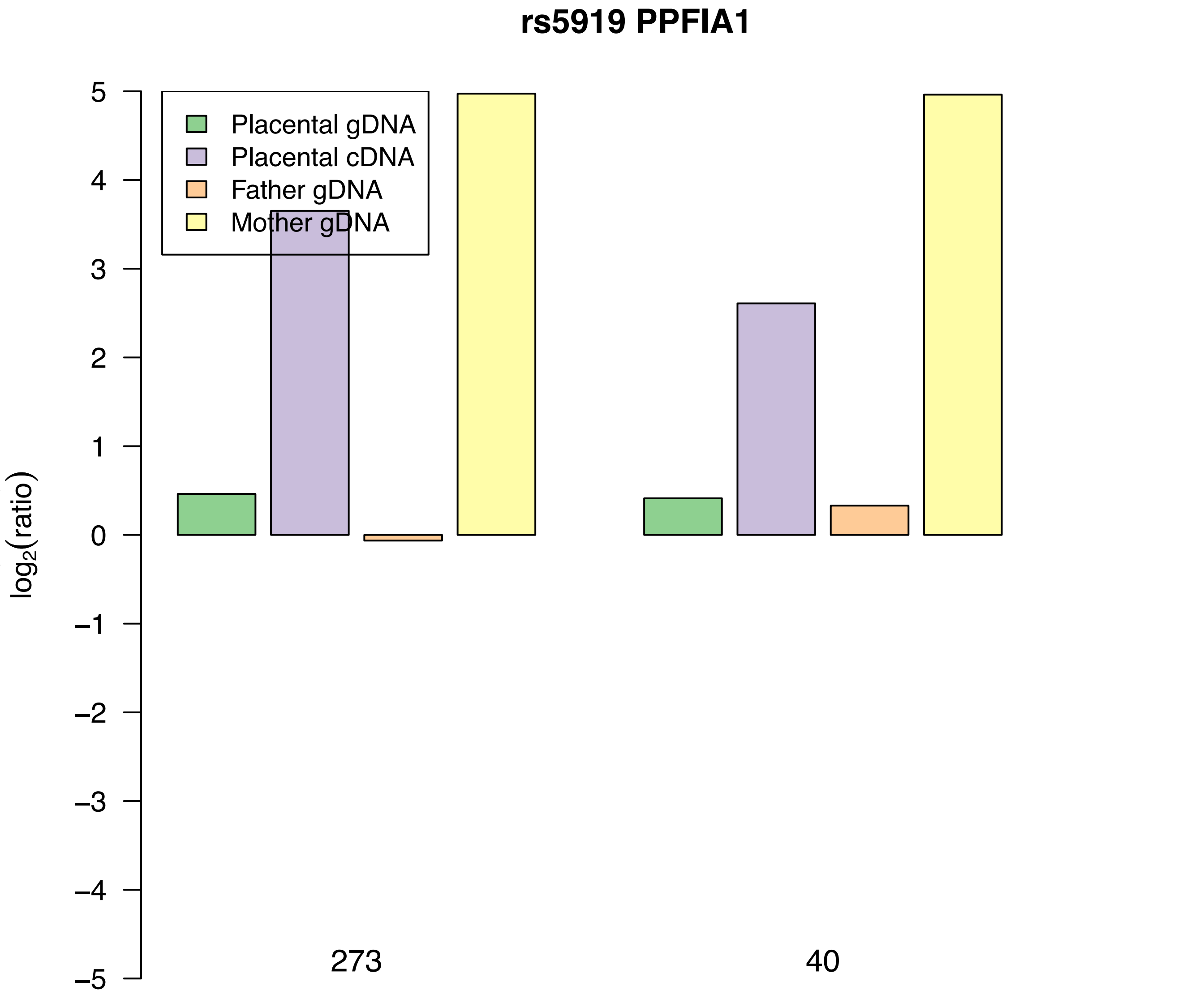
rs5919 PPFIA1
Placental gDNA
Placental cDNA
Father gDNA
Mother gDNA
log2(ratio)
5
4
3
2
1
0
−1
−2
−3
−4
−5
273
40

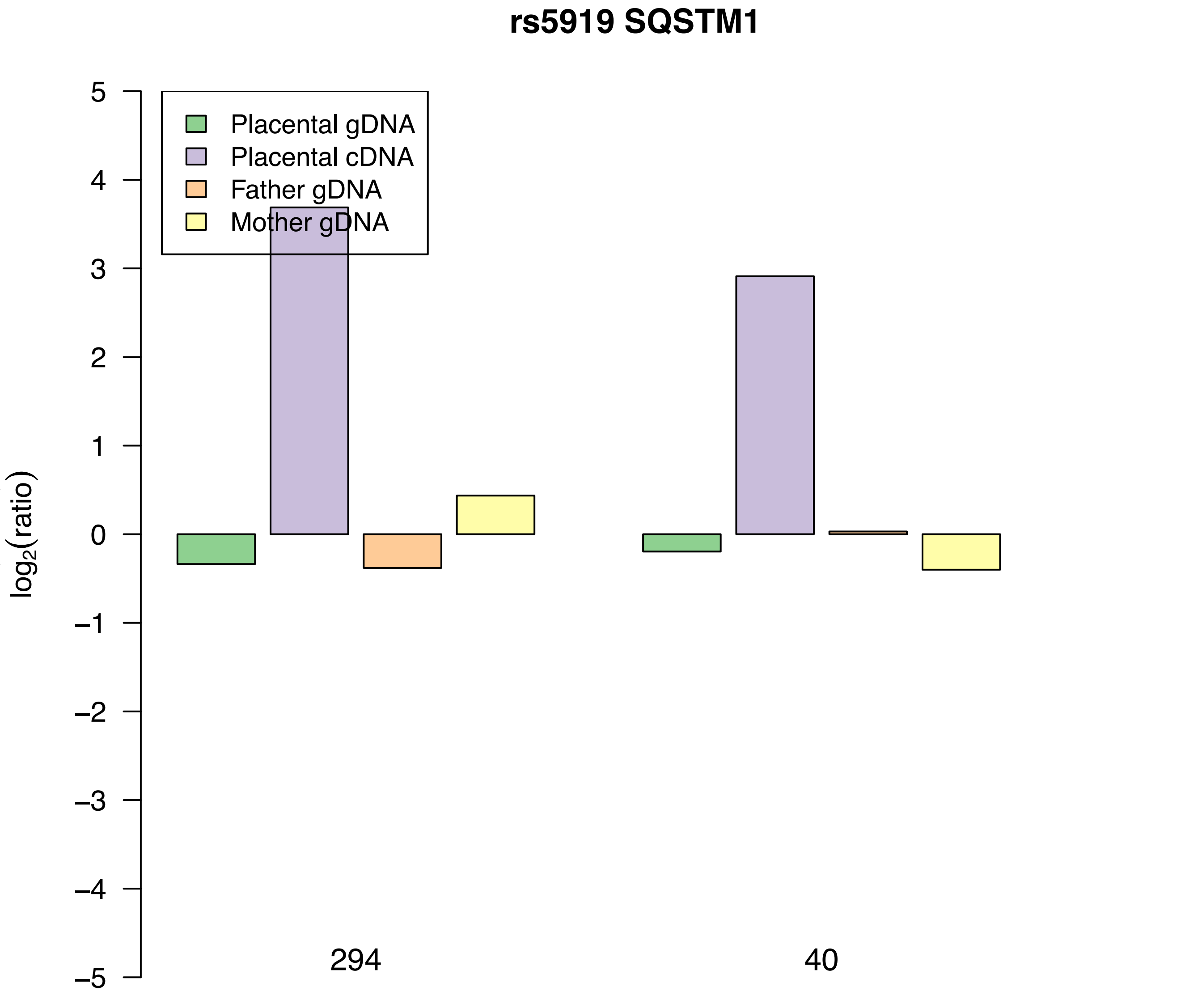
rs5919 SQSTM1
Placental gDNA
Placental cDNA
Father gDNA
Mother gDNA
log2(ratio)
5
4
3
2
1
0
−1
−2
−3
−4
−5
294
40

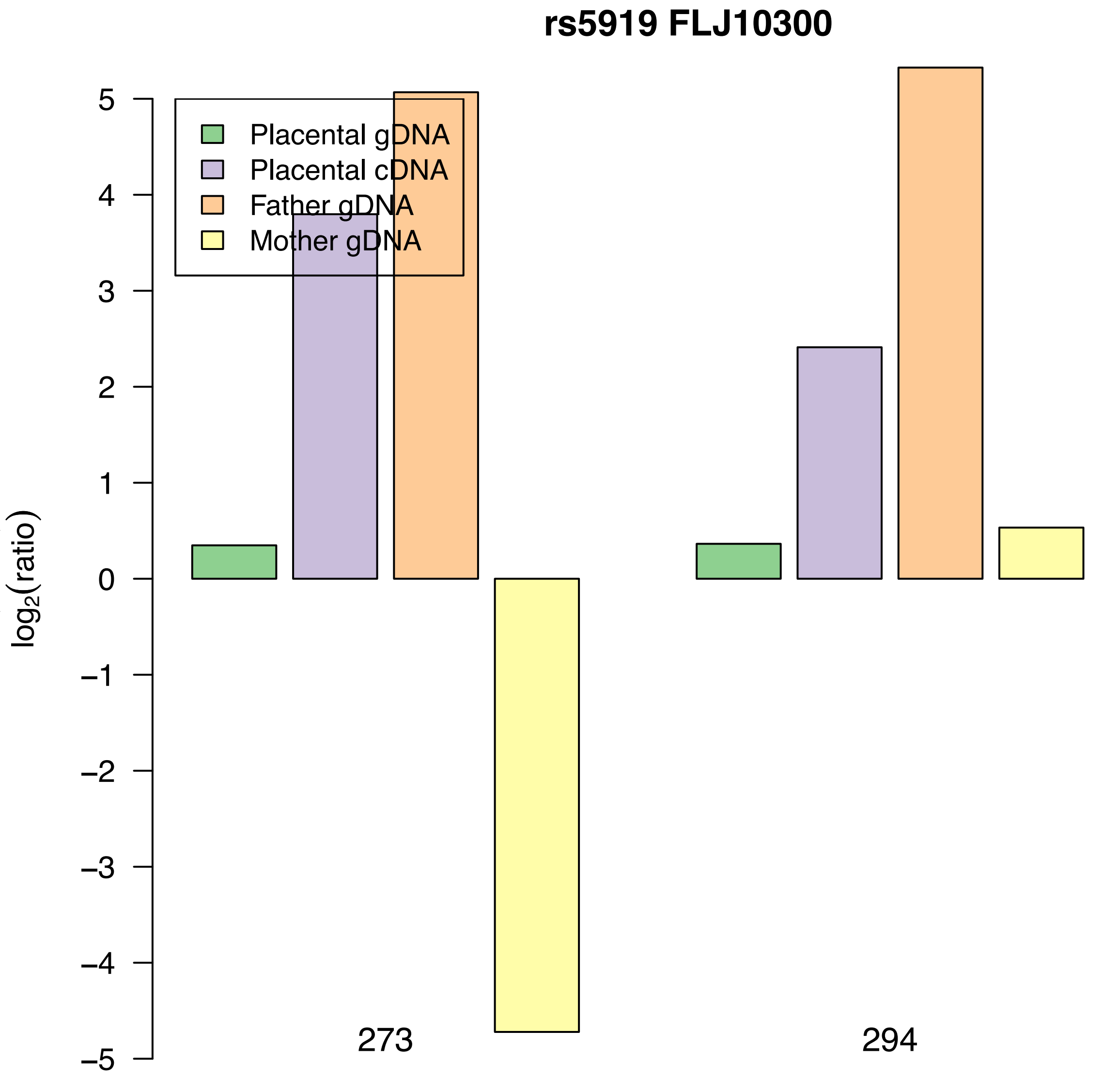

rs5919 FLJ10300
log2(ratio)
5
4
3
2
1
0
−1
−2
−3
−4
−5
Placental gDNA
Placental cDNA
Father gDNA
Mother gDNA
273
294

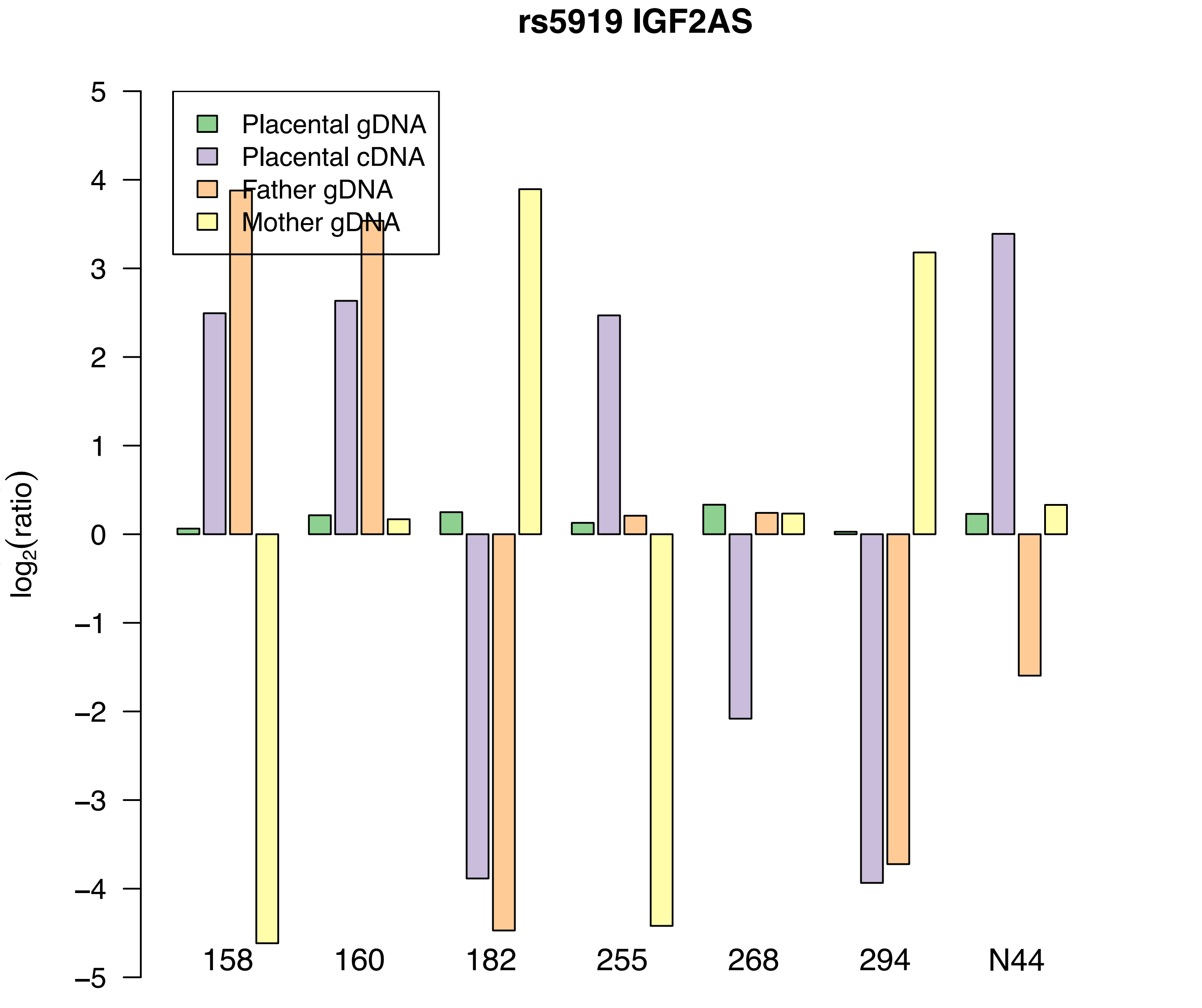
rs5919 IGF2AS
Placental gDNA
Placental cDNA
Father gDNA
Mother gDNA
log2(ratio)
5
4
3
2
1
0
−1
−2
−3
−4
−5
158
160
182
255
268
294
N44

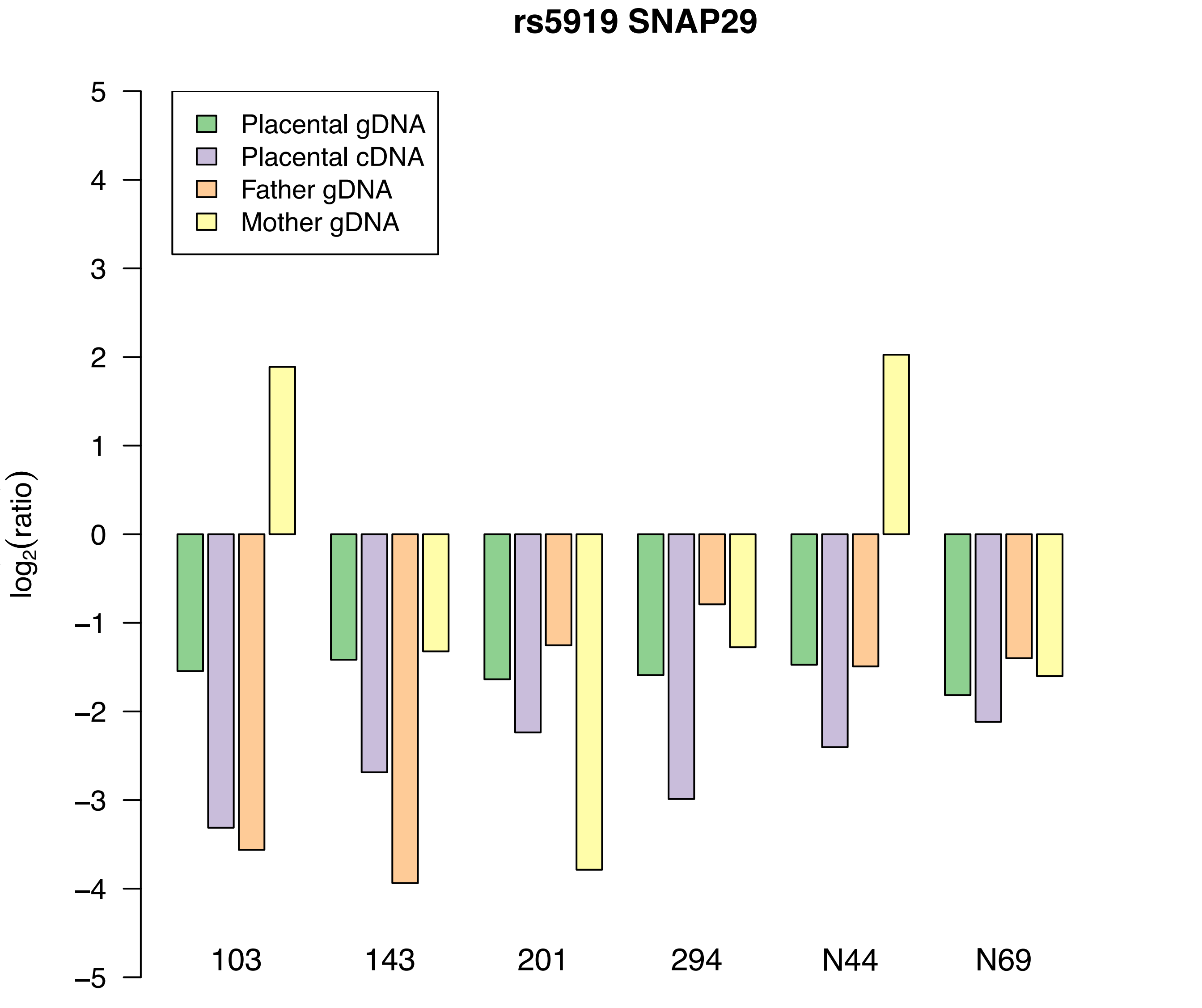

rs5919 SNAP29
Placental gDNA
Placental cDNA
Father gDNA
Mother gDNA
log2(ratio)
5
4
3
2
1
0
−1
−2
−3
−4
−5
103
143
201
294
N44
N69

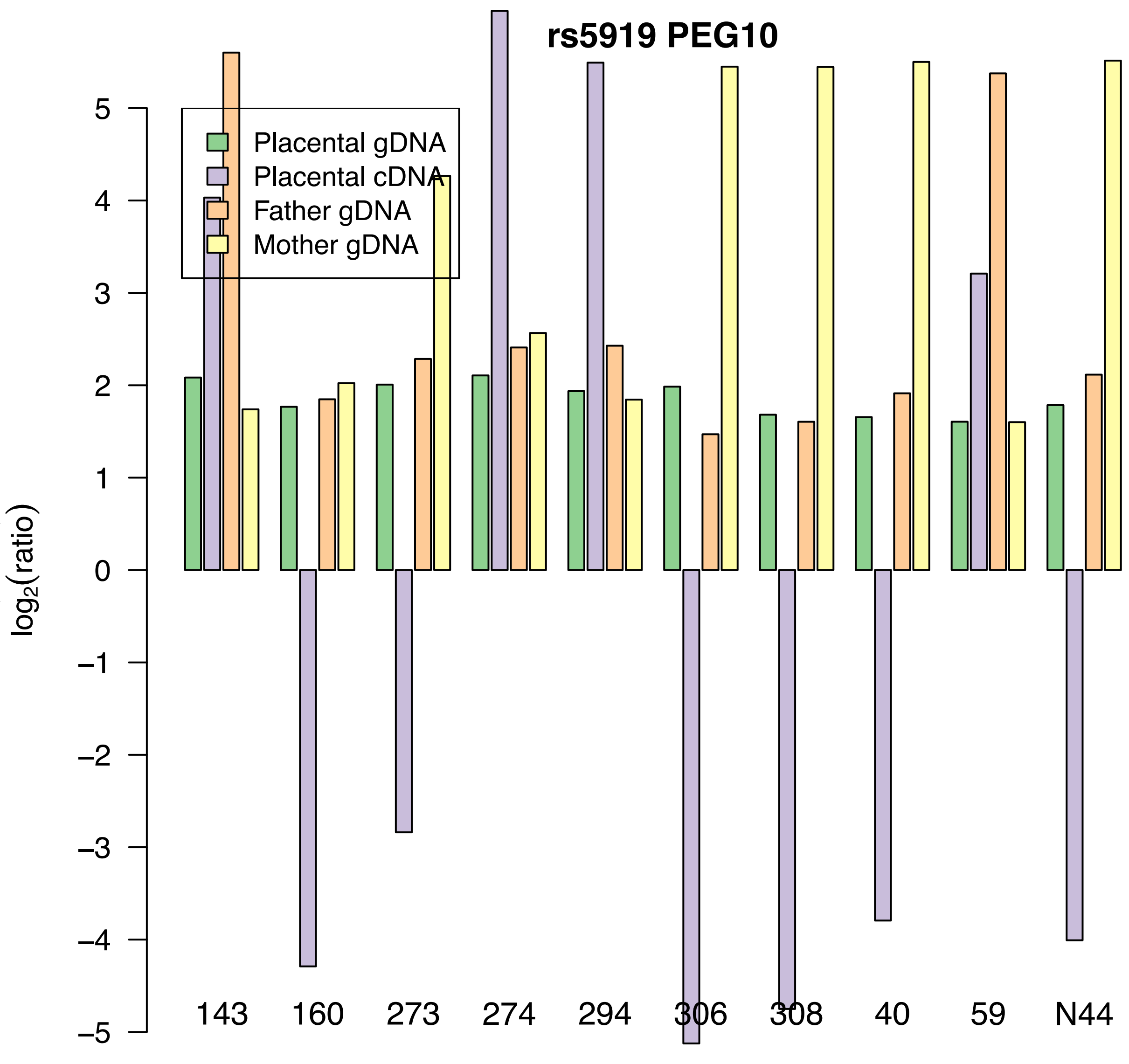

rs5919 PEG10
Placental gDNA
Placental cDNA
Father gDNA
Mother gDNA
log2(ratio)
5
4
3
2
1
0
−1
−2
−3
−4
−5
143
160
273
274
294
306
308
40
59
N44

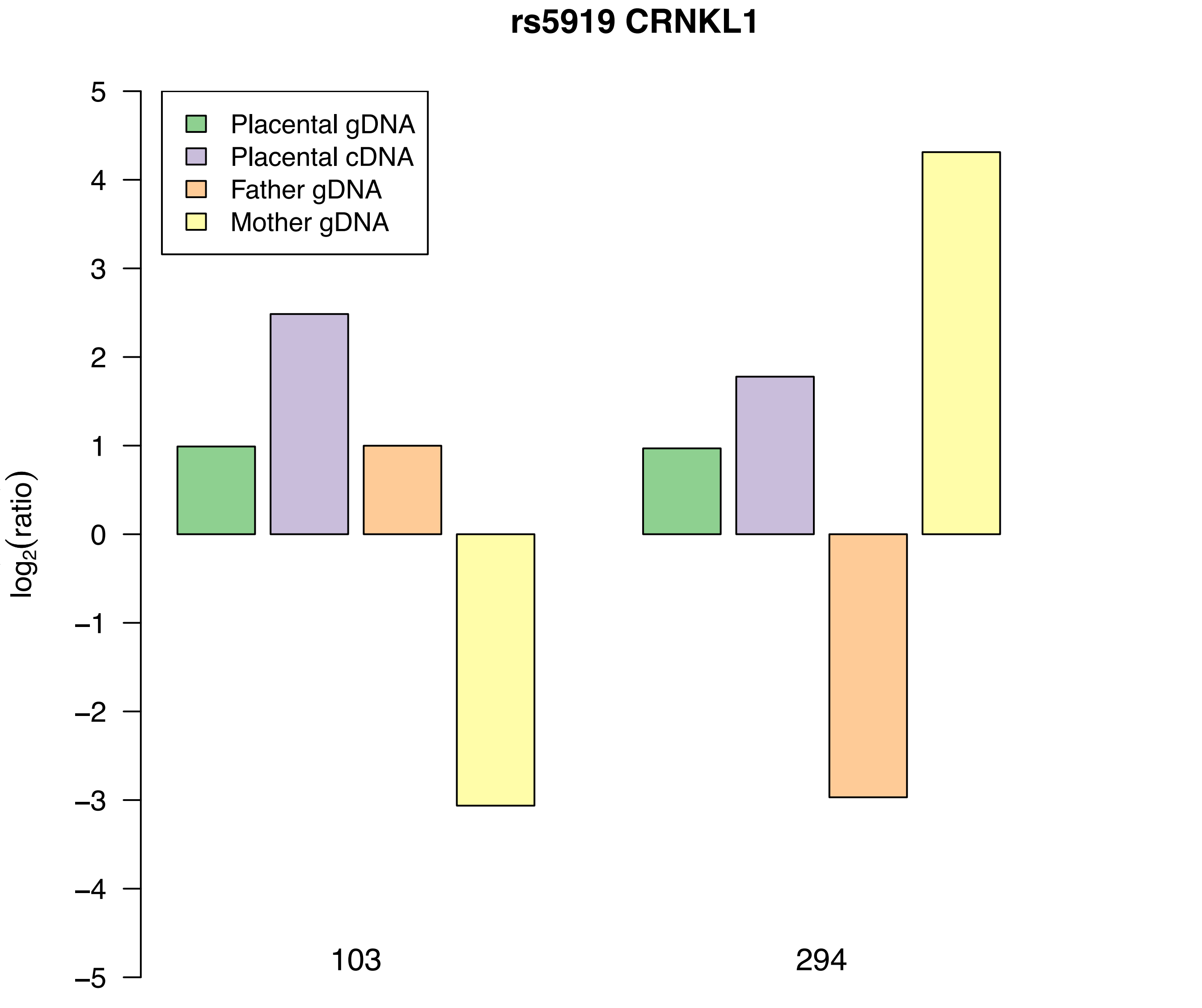
rs5919 CRNKL1
Placental gDNA
Placental cDNA
Father gDNA
Mother gDNA
log2(ratio)
5
4
3
2
1
0
−1
−2
−3
−4
−5
103
294

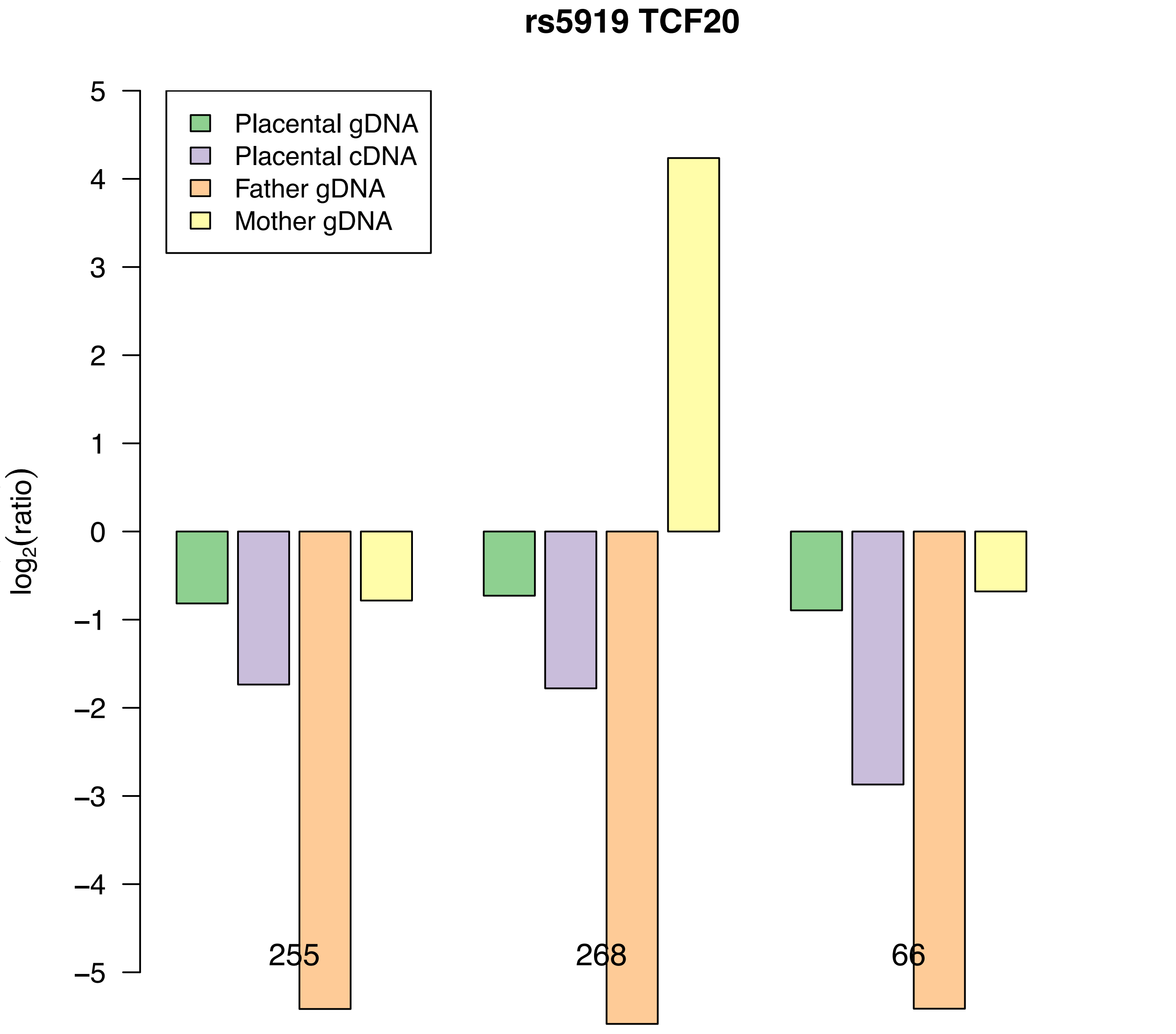

rs5919 TCF20
Placental gDNA
Placental cDNA
Father gDNA
Mother gDNA
log2(ratio)
5
4
3
2
1
0
−1
−2
−3
−4
−5
255
268
66

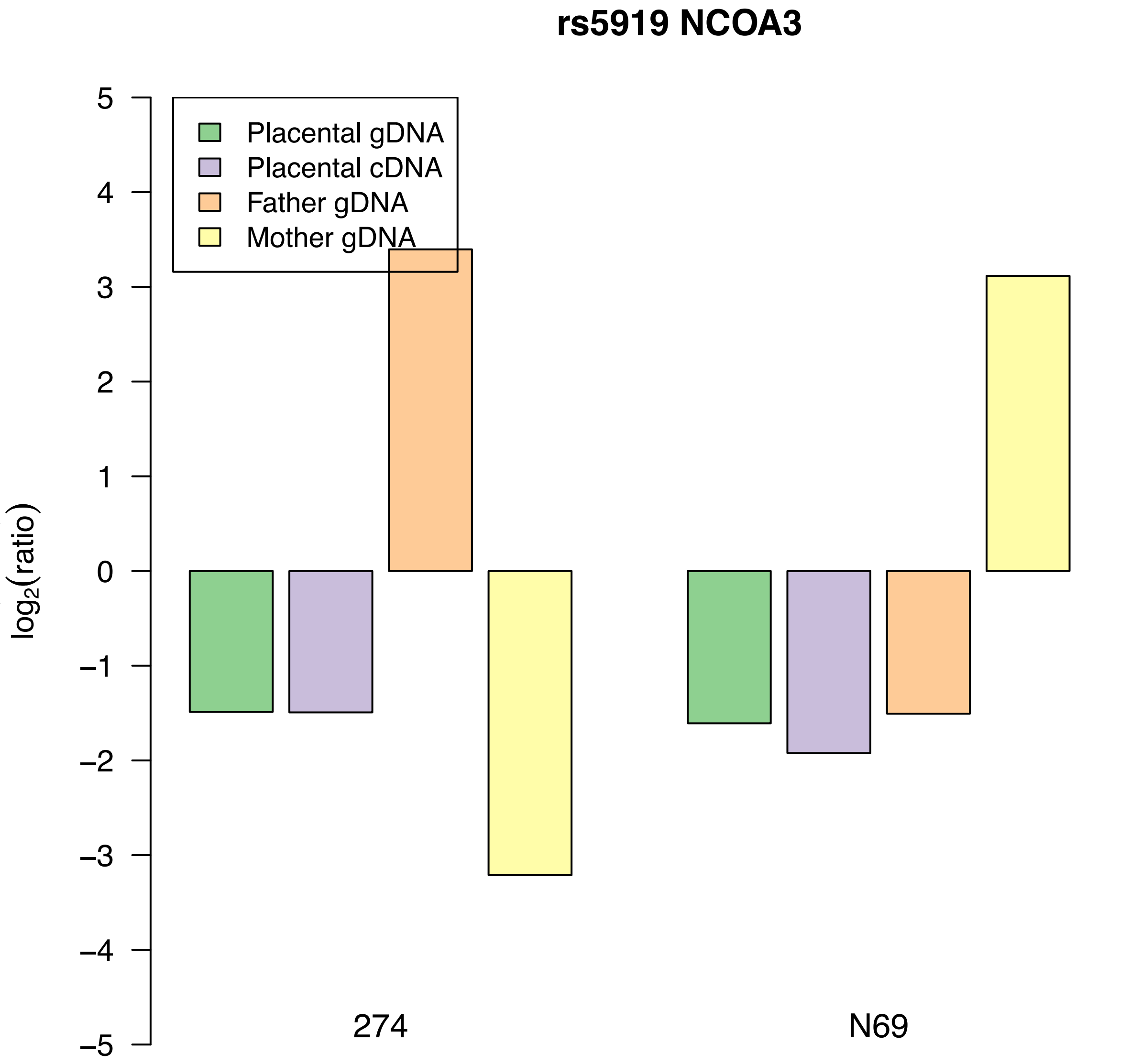

rs5919 NCOA3
Placental gDNA
Placental cDNA
Father gDNA
Mother gDNA
$\log_2$(ratio)
5
4
3
2
1
0
−1
−2
−3
−4
−5
274
N69

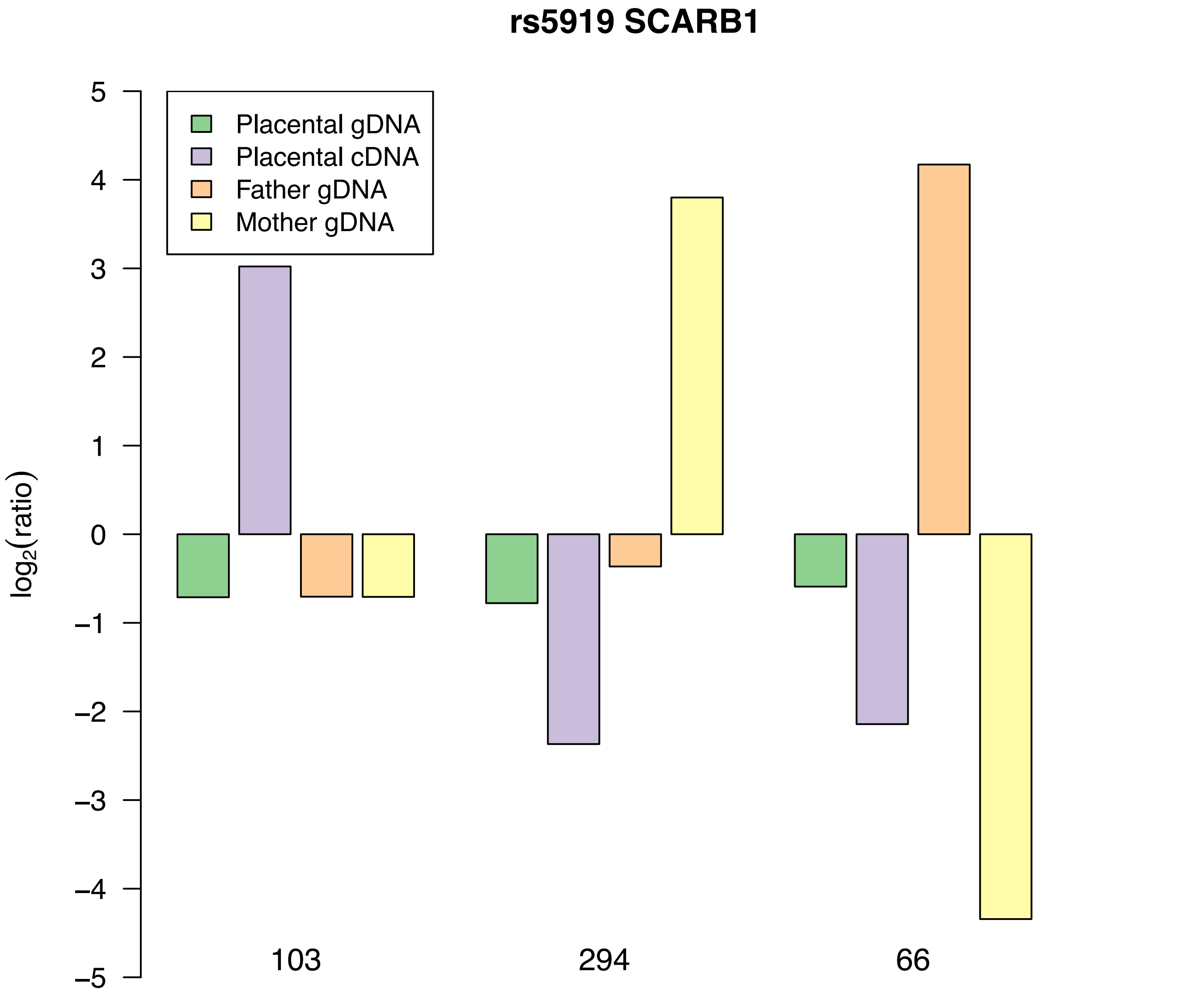

rs5919 SCARB1
Placental gDNA
Placental cDNA
Father gDNA
Mother gDNA
5
4
3
2
1
0
−1
−2
−3
−4
−5
log2(ratio)
103
294
66

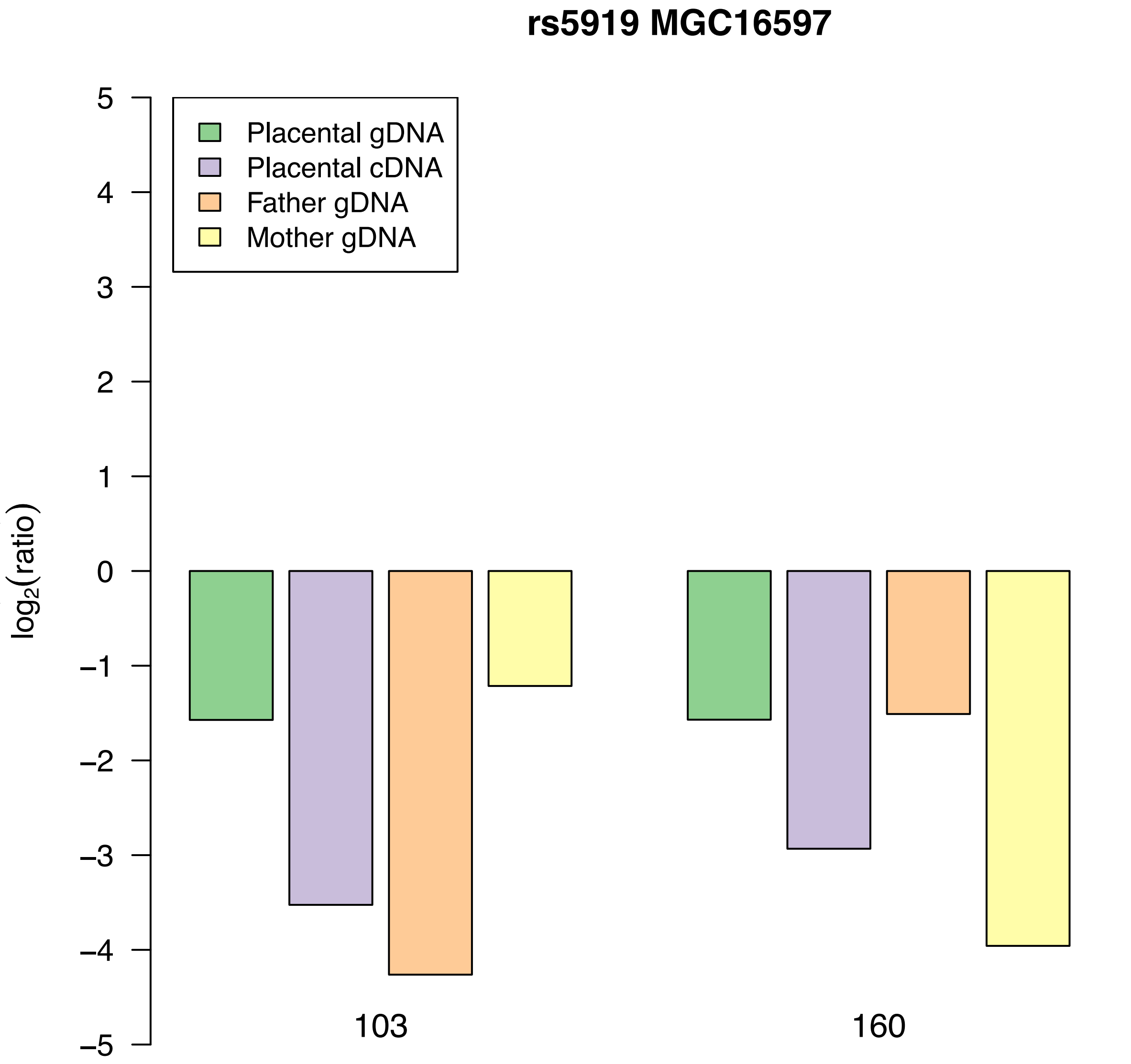
rs5919 MGC16597
Placental gDNA
Placental cDNA
Father gDNA
Mother gDNA
log2(ratio)
5
4
3
2
1
0
−1
−2
−3
−4
−5
103
160

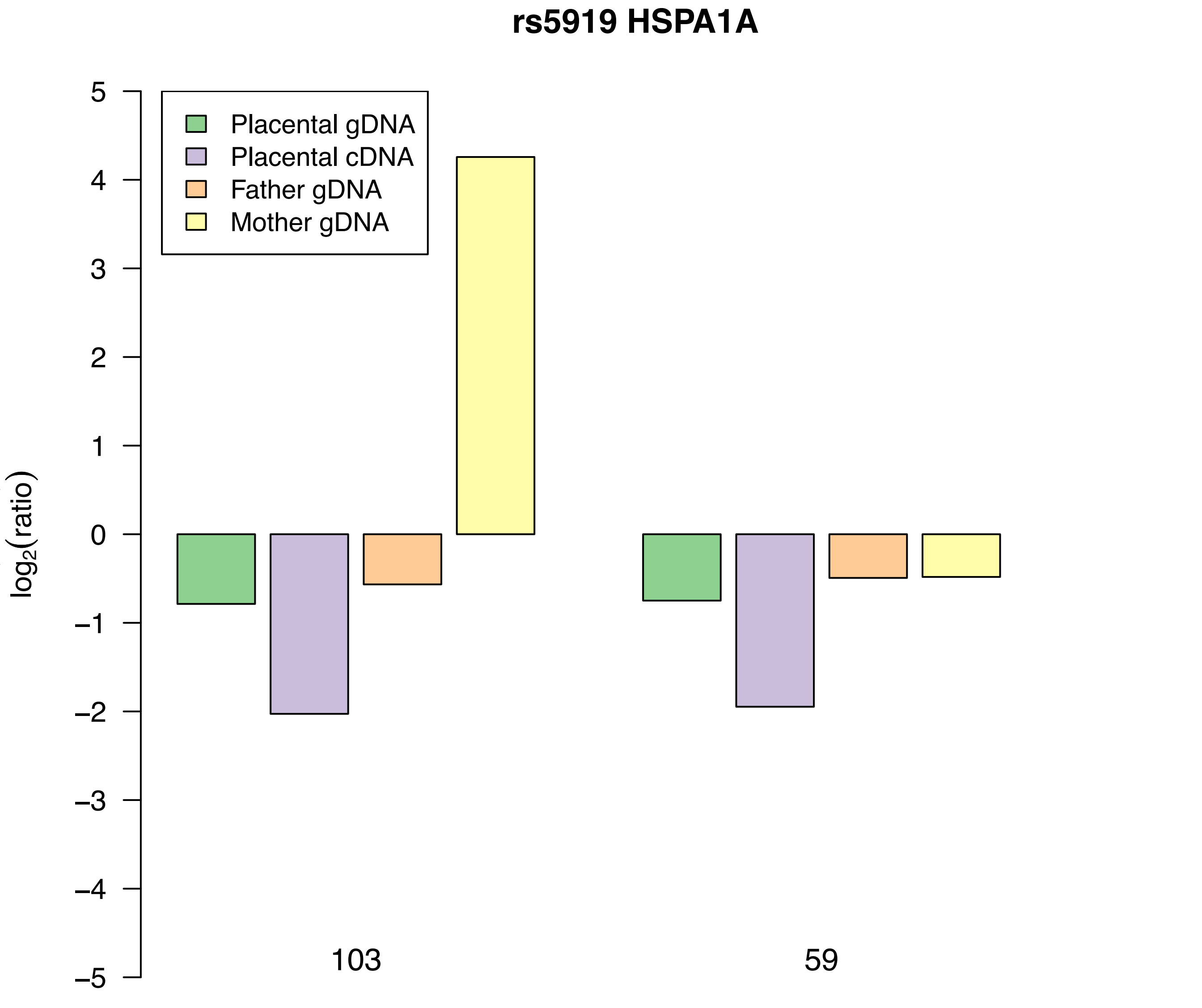

rs5919 HSPA1A
Placental gDNA
Placental cDNA
Father gDNA
Mother gDNA
log2(ratio)
5
4
3
2
1
0
−1
−2
−3
−4
−5
103
59

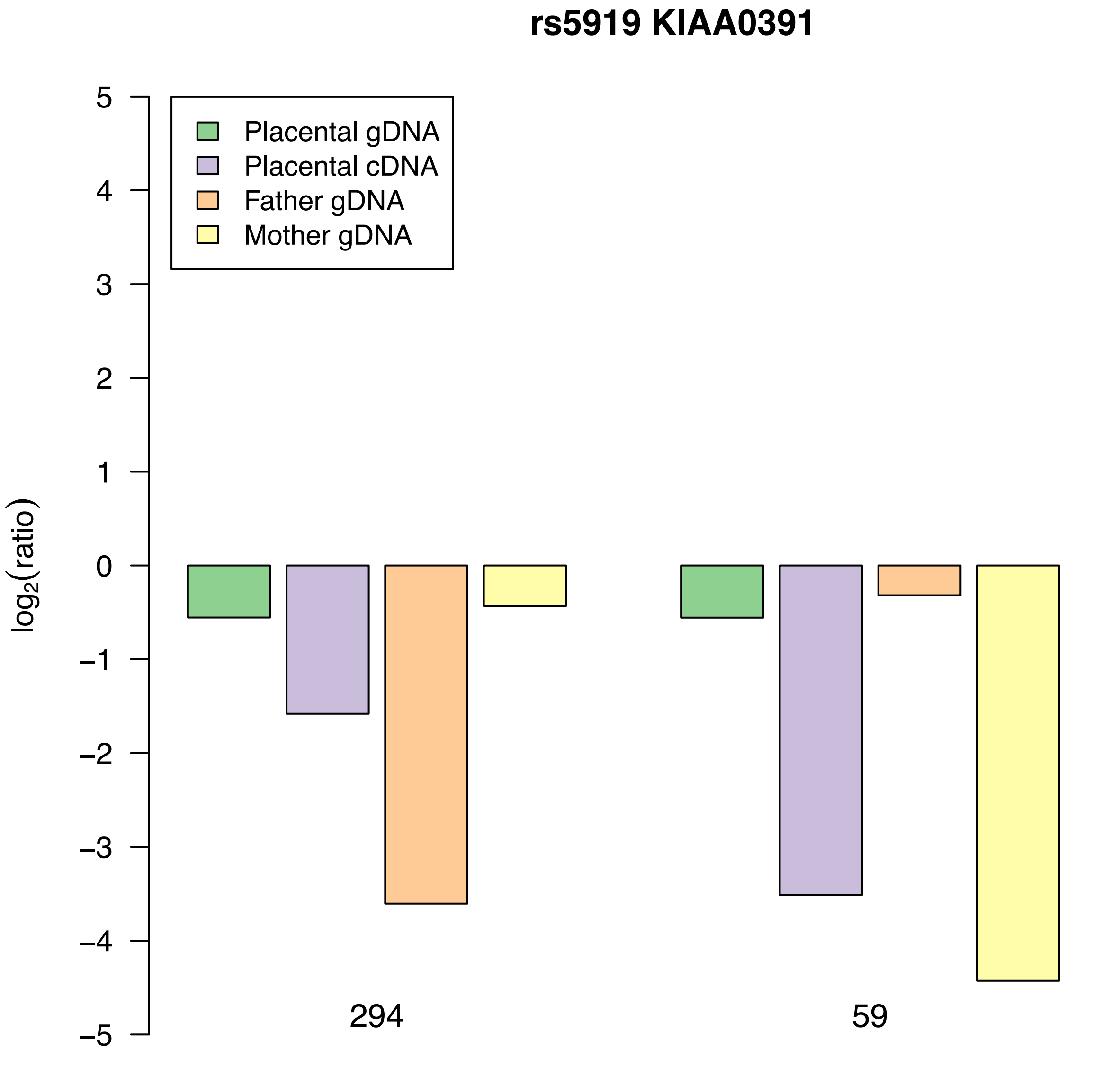
rs5919 KIAA0391
Placental gDNA
Placental cDNA
Father gDNA
Mother gDNA
$\log_2$(ratio)
5
4
3
2
1
0
−1
−2
−3
−4
−5
294
59

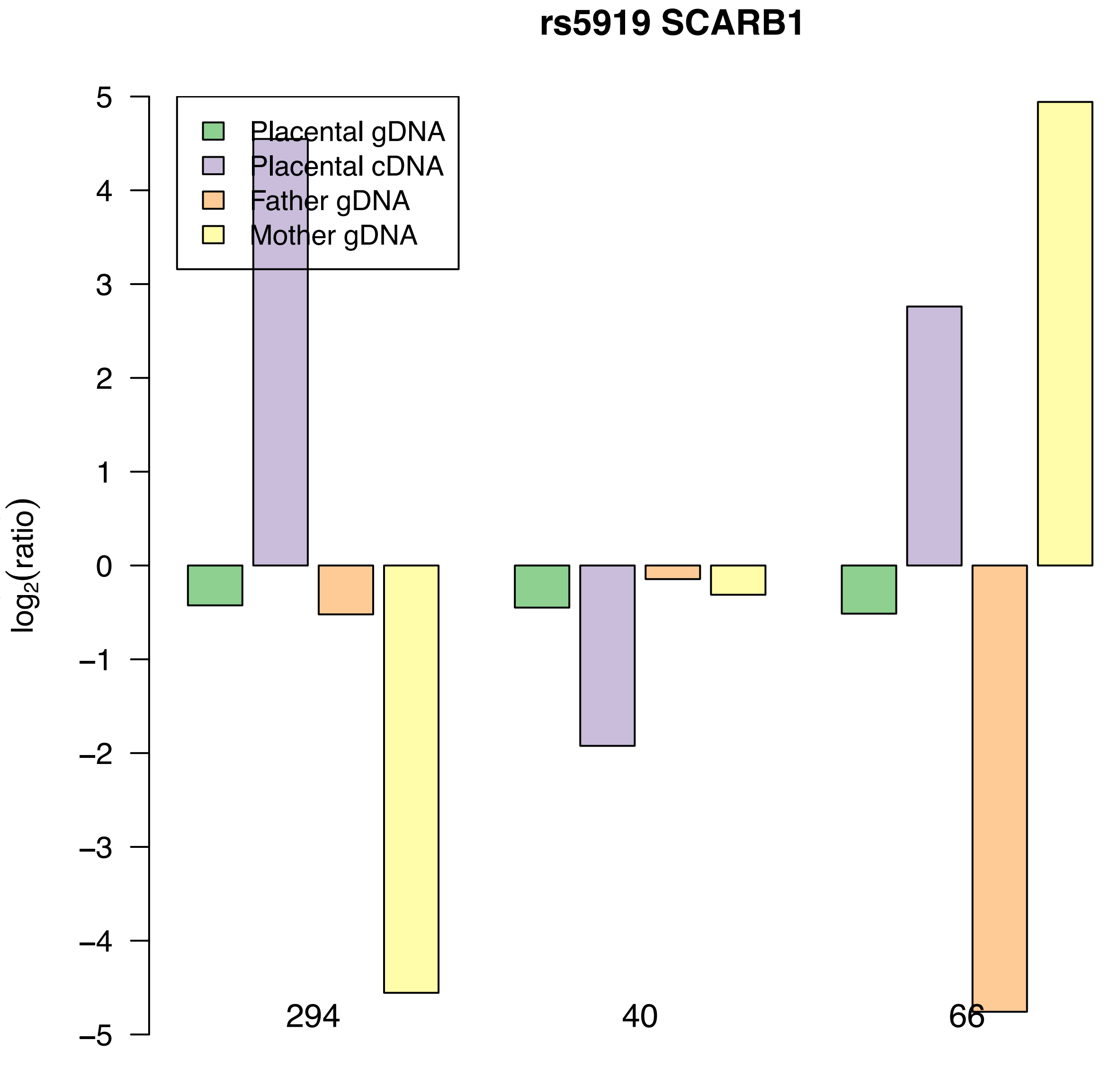

rs5919 SCARB1
Placental gDNA
Placental cDNA
Father gDNA
Mother gDNA
log2(ratio)
5
4
3
2
1
0
−1
−2
−3
−4
−5
294
40
66

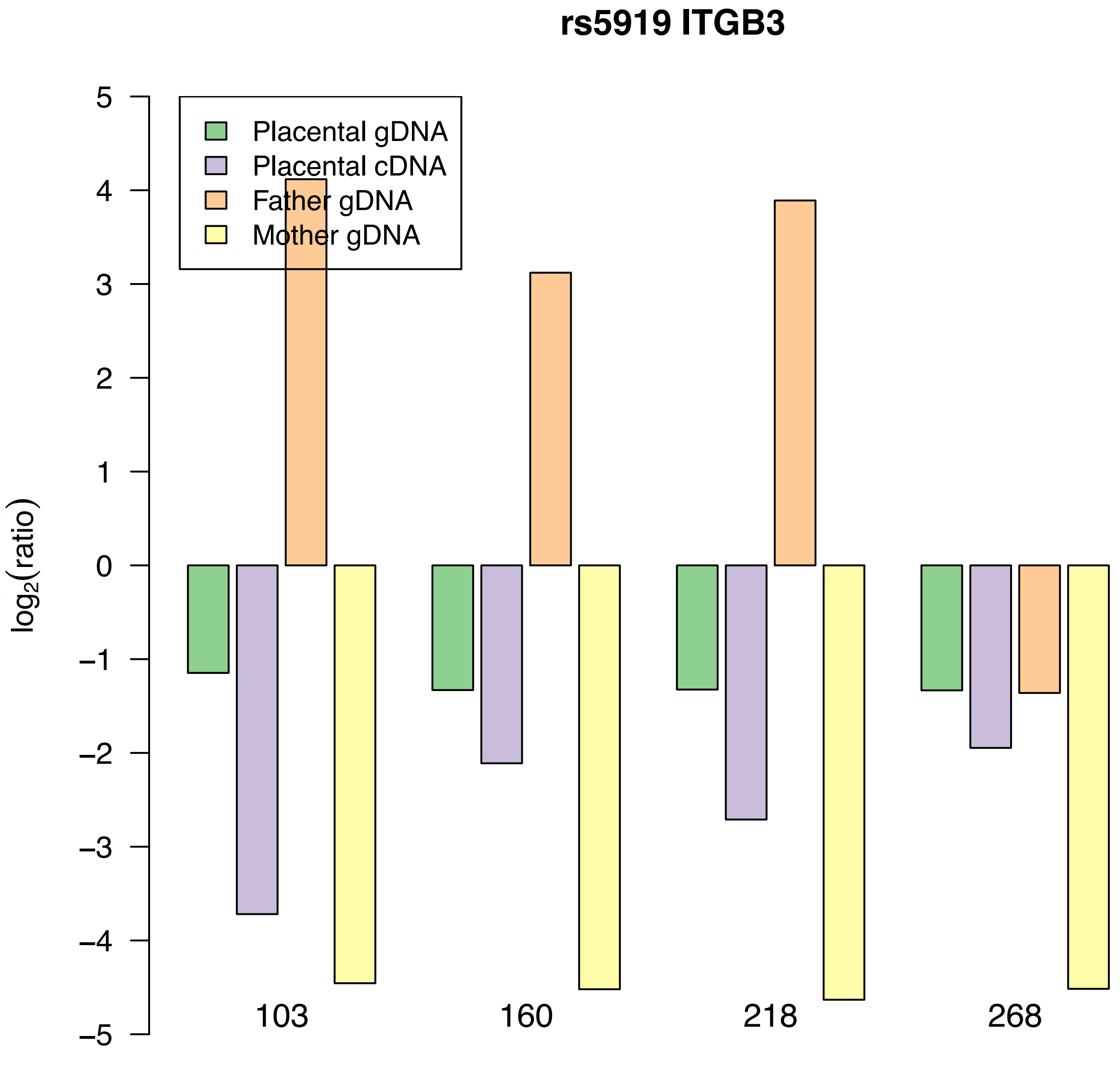

rs5919 ITGB3
Placental gDNA
Placental cDNA
Father gDNA
Mother gDNA
log2(ratio)
5
4
3
2
1
0
−1
−2
−3
−4
−5
103
160
218
268

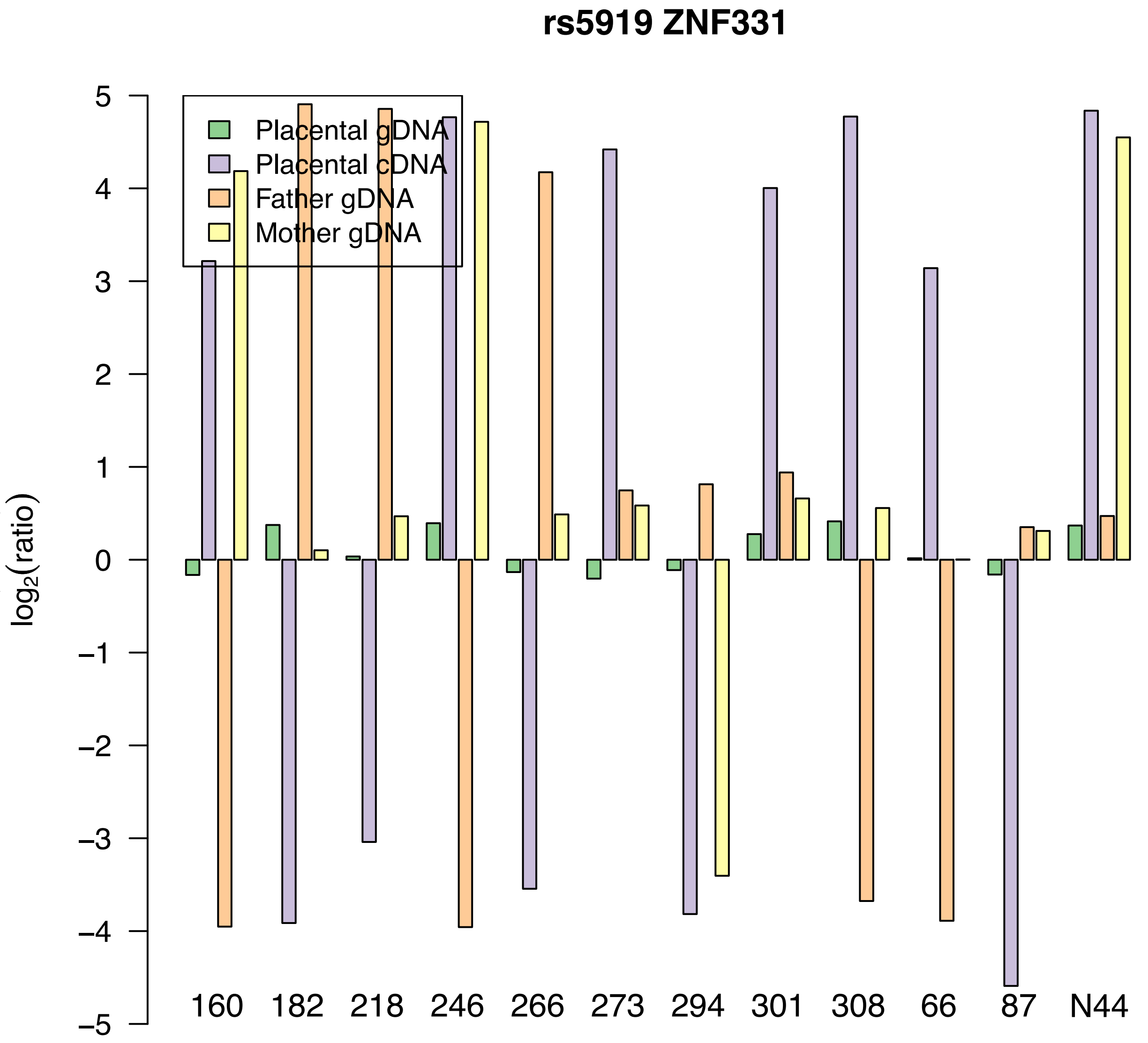
rs5919 ZNF331
Placental gDNA
Placental cDNA
Father gDNA
Mother gDNA
5
4
3
2
1
0
−1
−2
−3
−4
−5
log2(ratio)
160
182
218
246
266
273
294
301
308
66
87
N44

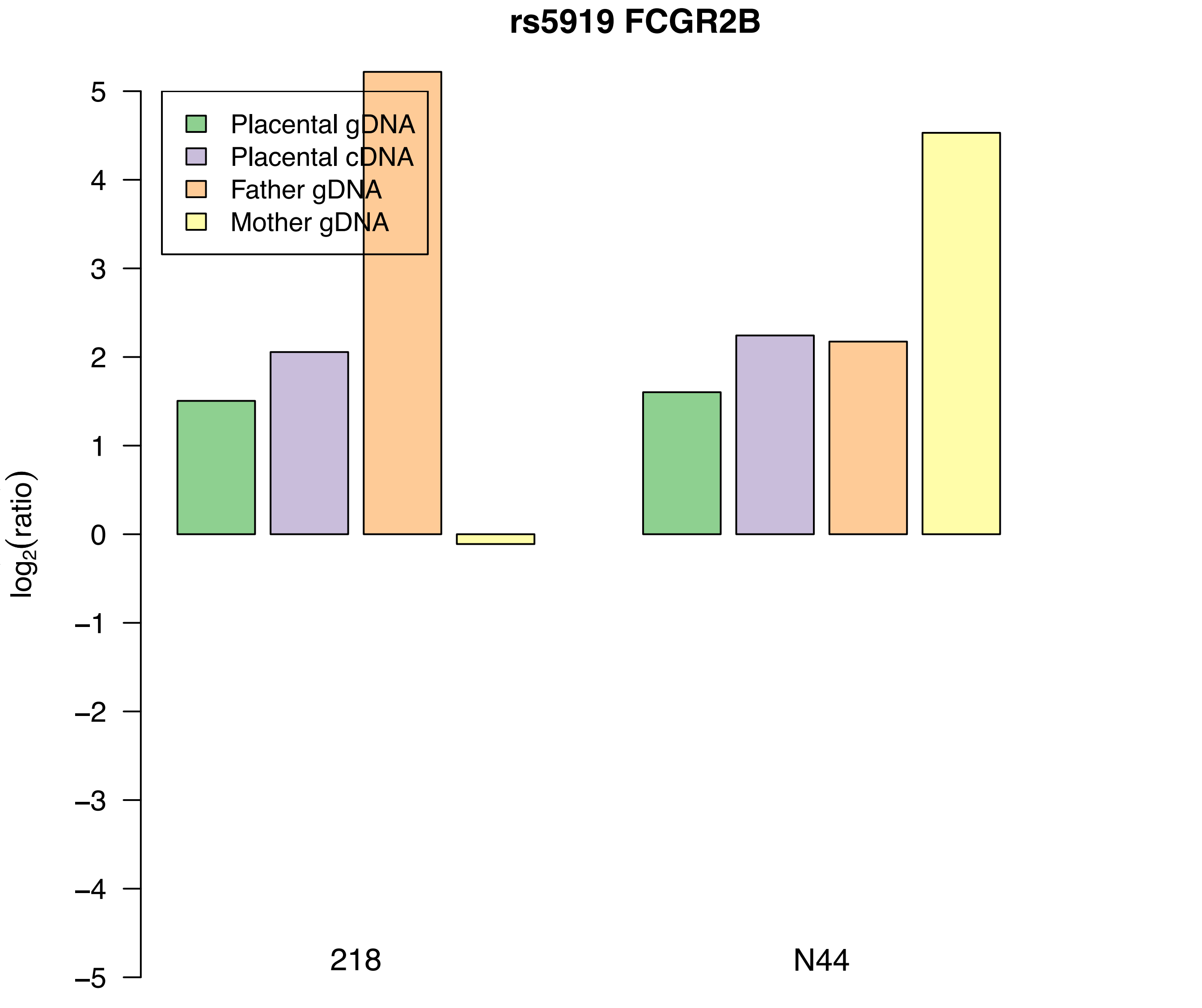

rs5919 FCGR2B
Placental gDNA
Placental cDNA
Father gDNA
Mother gDNA
log2(ratio)
5
4
3
2
1
0
−1
−2
−3
−4
−5
218
N44

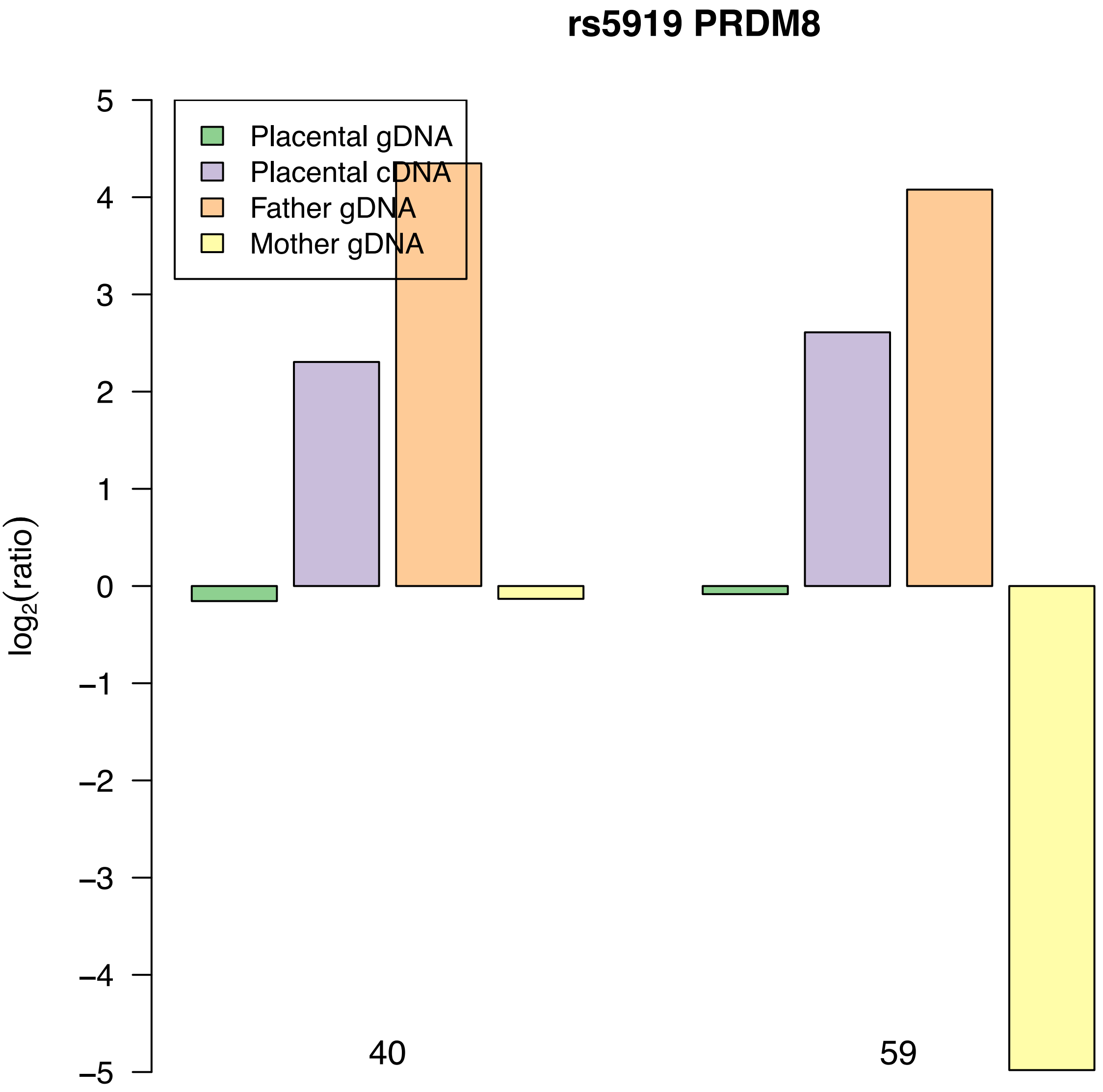

rs5919 PRDM8
Placental gDNA
Placental cDNA
Father gDNA
Mother gDNA
log2(ratio)
5
4
3
2
1
0
−1
−2
−3
−4
−5
40
59

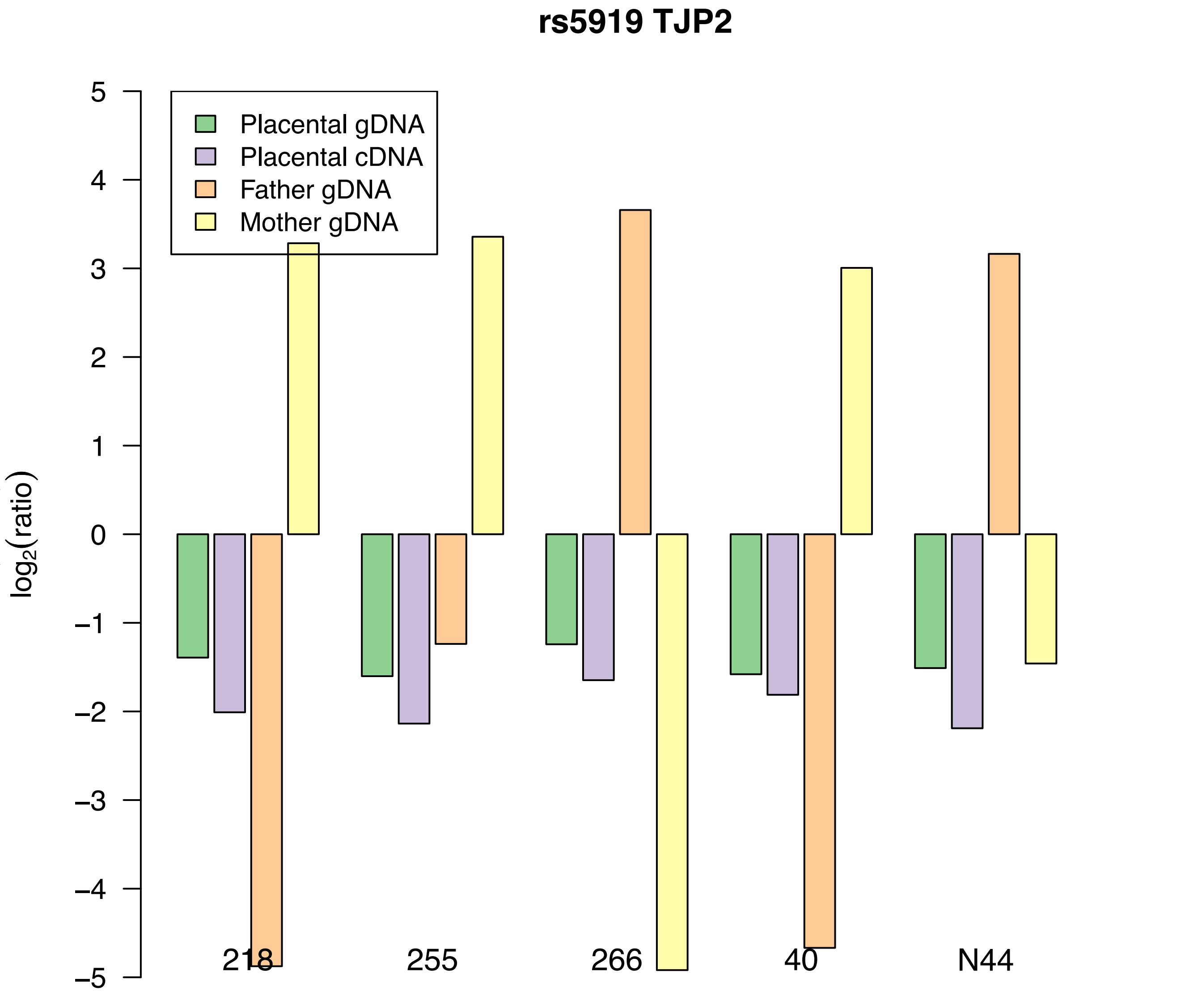
rs5919 TJP2
Placental gDNA
Placental cDNA
Father gDNA
Mother gDNA
log2(ratio)
5
4
3
2
1
0
−1
−2
−3
−4
−5
218
255
266
40
N44

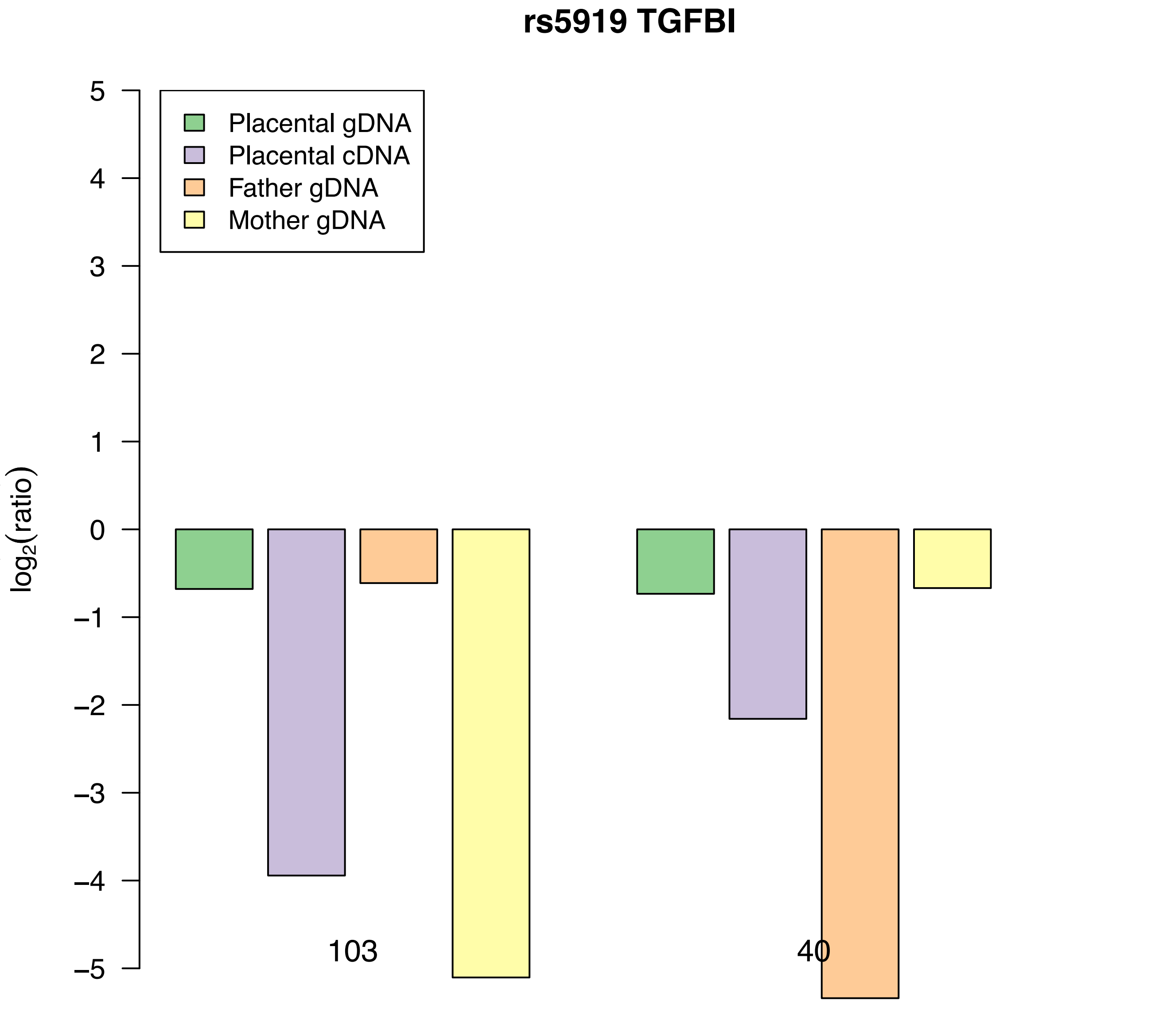
rs5919 TGFBI
Placental gDNA
Placental cDNA
Father gDNA
Mother gDNA
log2(ratio)
5
4
3
2
1
0
−1
−2
−3
−4
−5
103
40

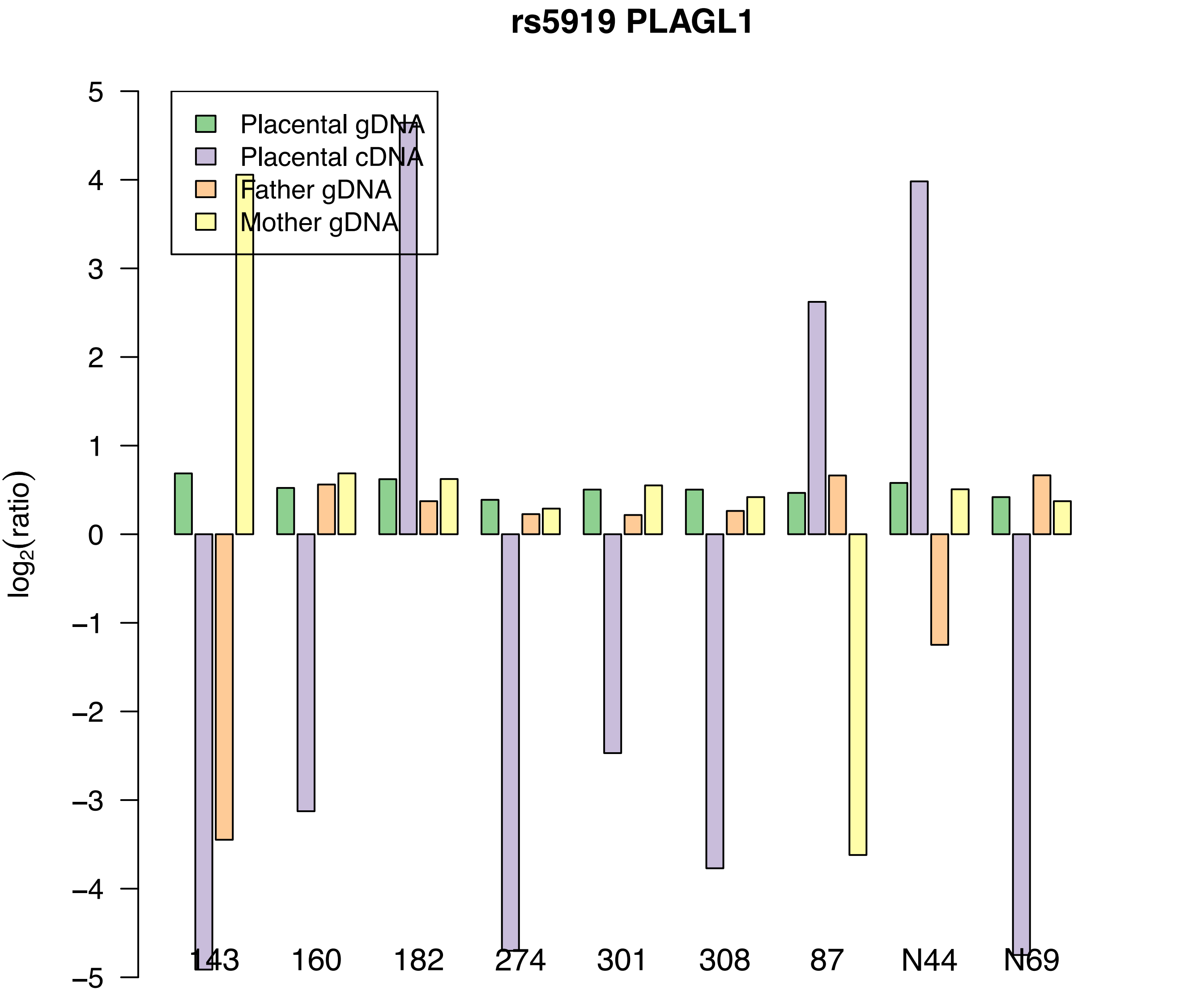

rs5919 PLAGL1
Placental gDNA
Placental cDNA
Father gDNA
Mother gDNA
log2(ratio)
5
4
3
2
1
0
−1
−2
−3
−4
−5
143
160
182
274
301
308
87
N44
N69

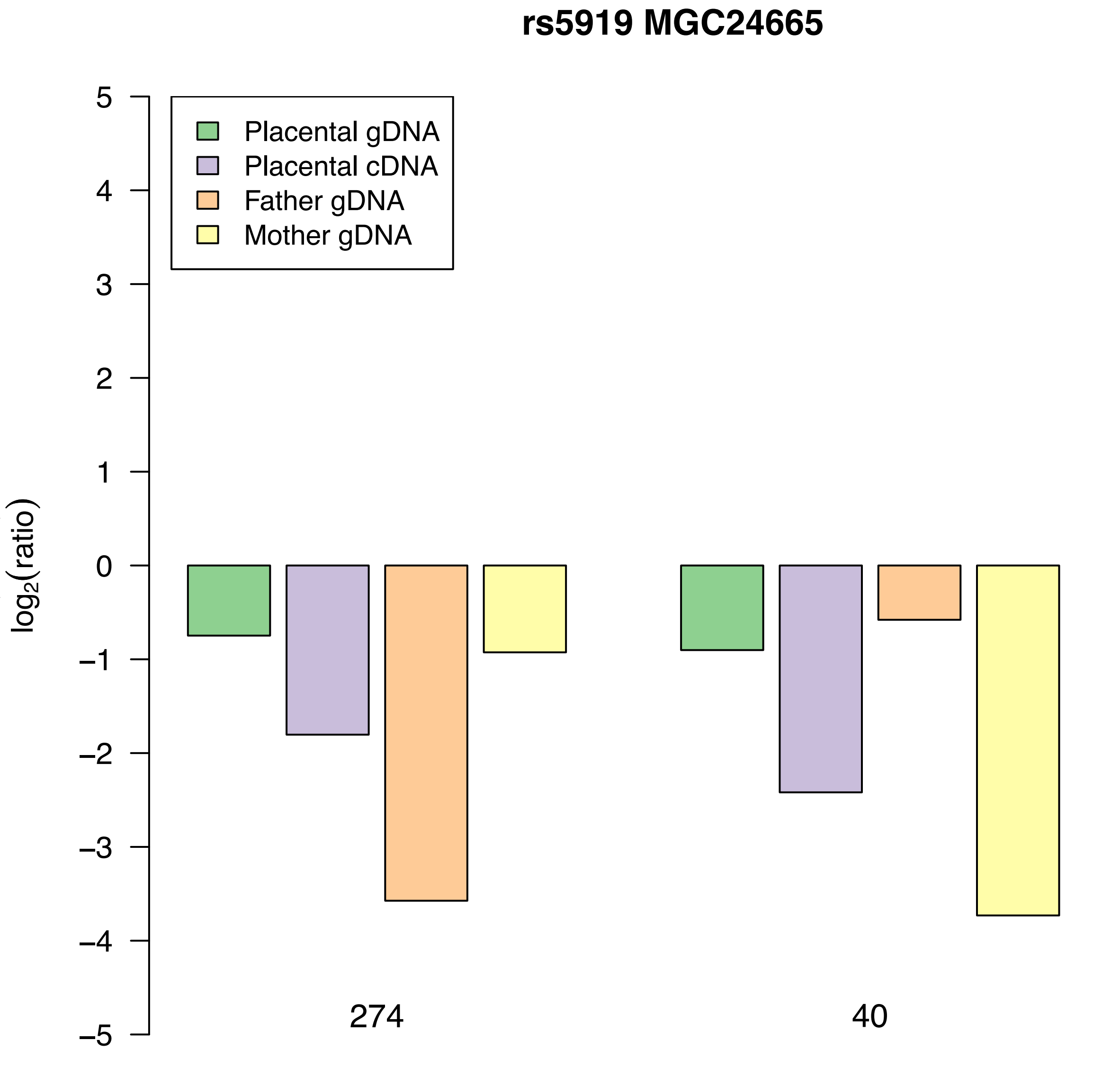
rs5919 MGC24665
log2(ratio)
5
4
3
2
1
0
−1
−2
−3
−4
−5
Placental gDNA
Placental cDNA
Father gDNA
Mother gDNA
274
40

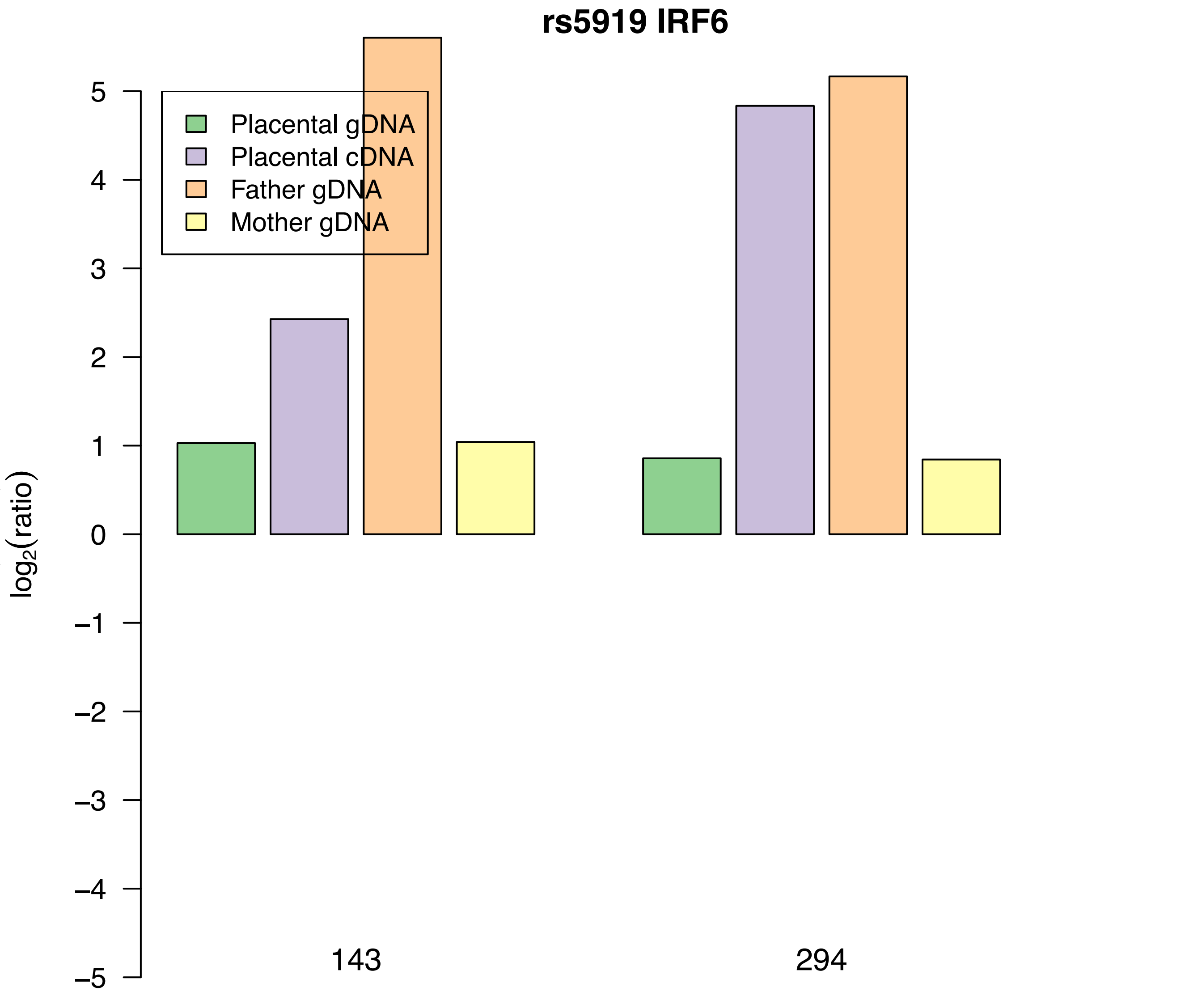

rs5919 IRF6
Placental gDNA
Placental cDNA
Father gDNA
Mother gDNA
log2(ratio)
5
4
3
2
1
0
−1
−2
−3
−4
−5
143
294

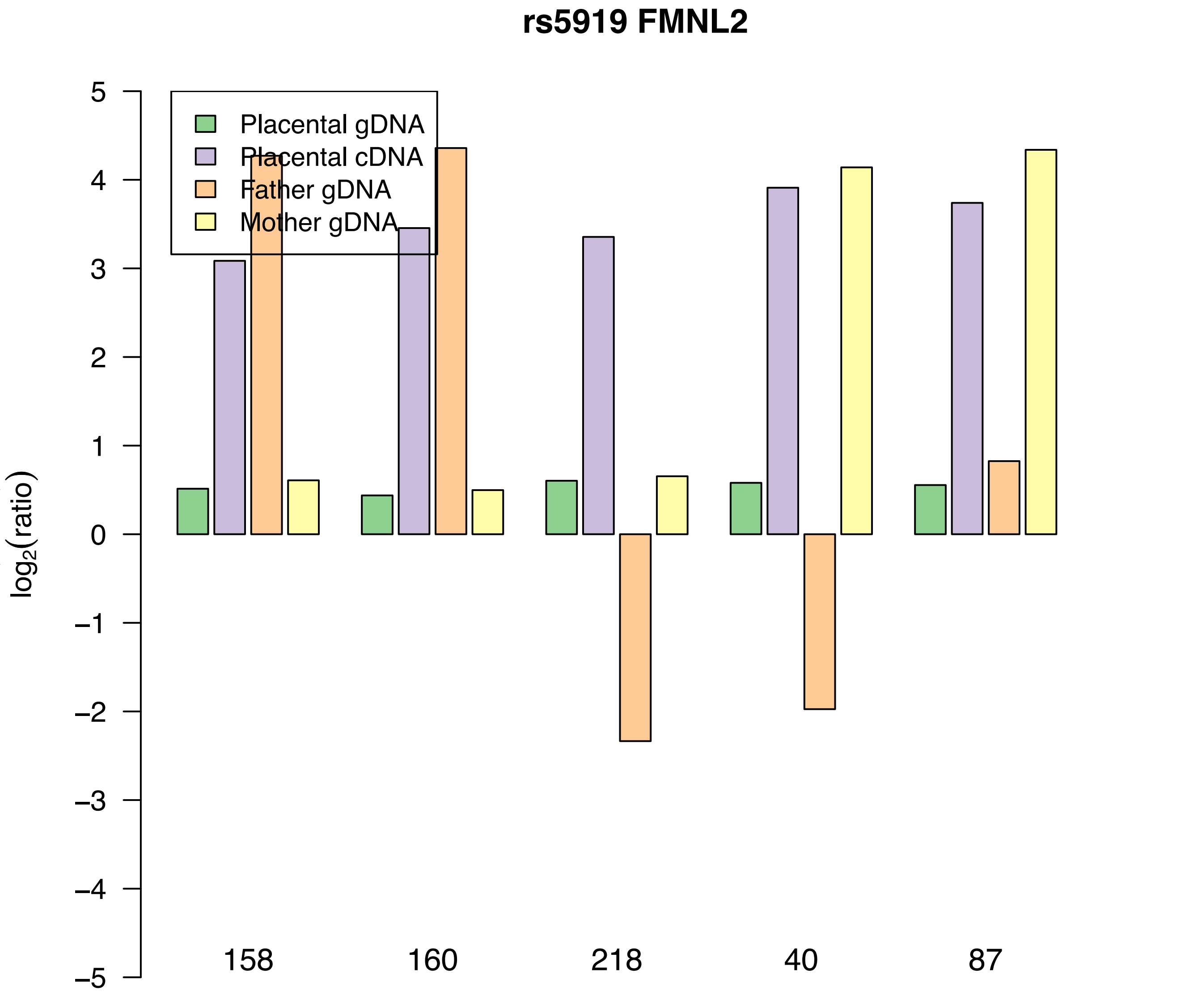
rs5919 FMNL2
Placental gDNA
Placental cDNA
Father gDNA
Mother gDNA
log2(ratio)
5
4
3
2
1
0
−1
−2
−3
−4
−5
158
160
218
40
87

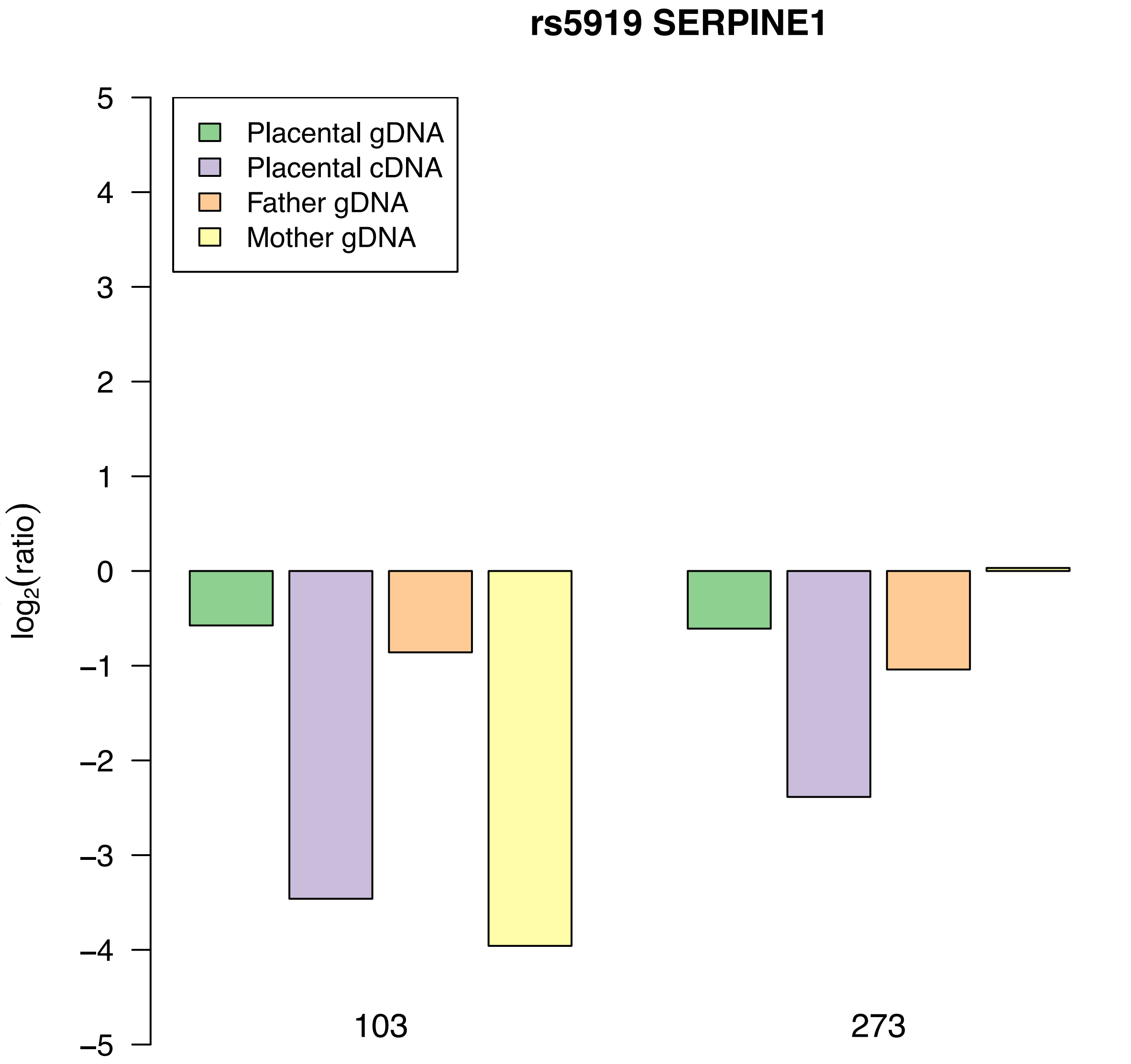

rs5919 SERPINE1
Placental gDNA
Placental cDNA
Father gDNA
Mother gDNA
log2(ratio)
5
4
3
2
1
0
−1
−2
−3
−4
−5
103
273

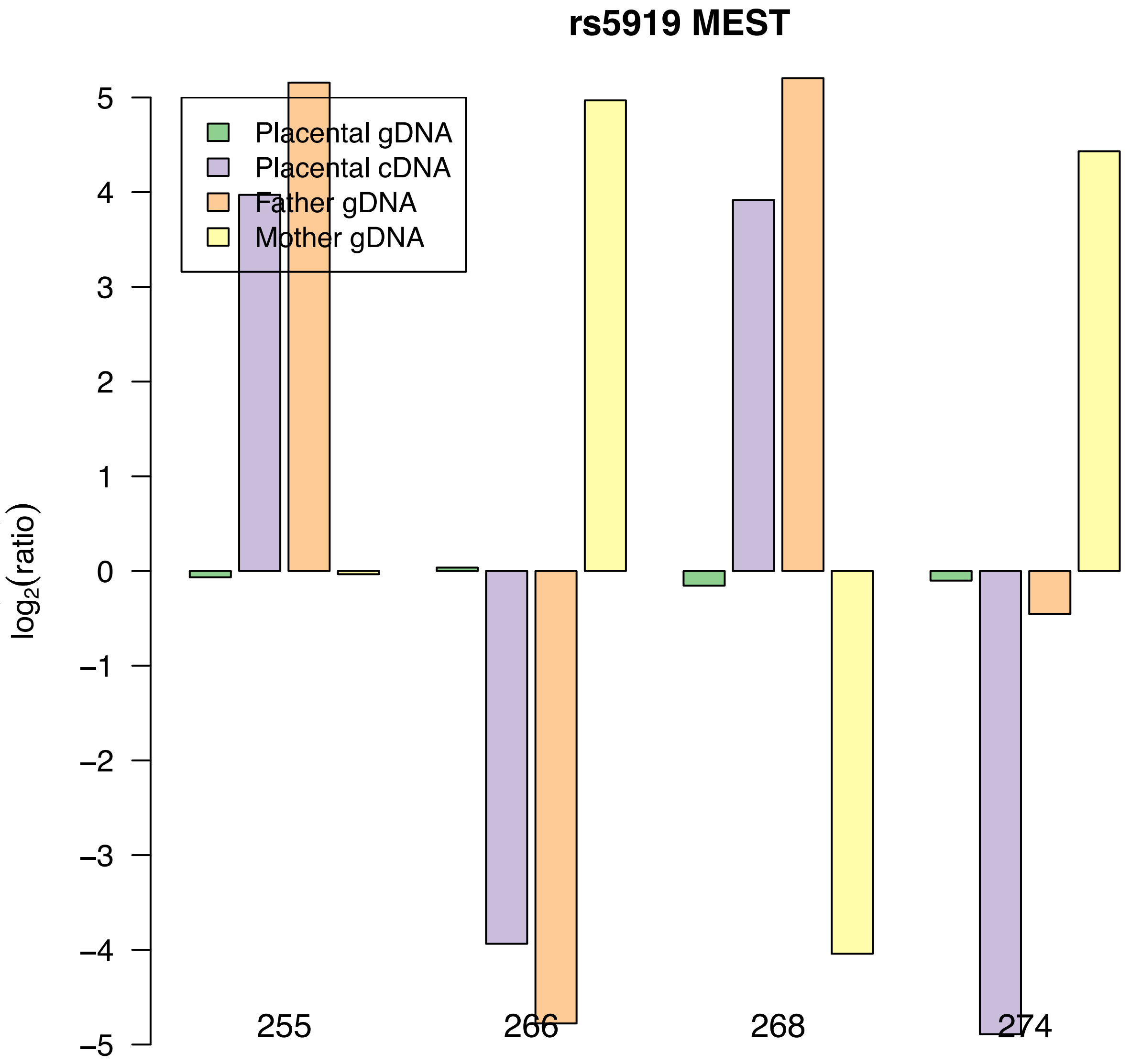
rs5919 MEST
Placental gDNA
Placental cDNA
Father gDNA
Mother gDNA
log2(ratio)
5
4
3
2
1
0
−1
−2
−3
−4
−5
255
266
268
274

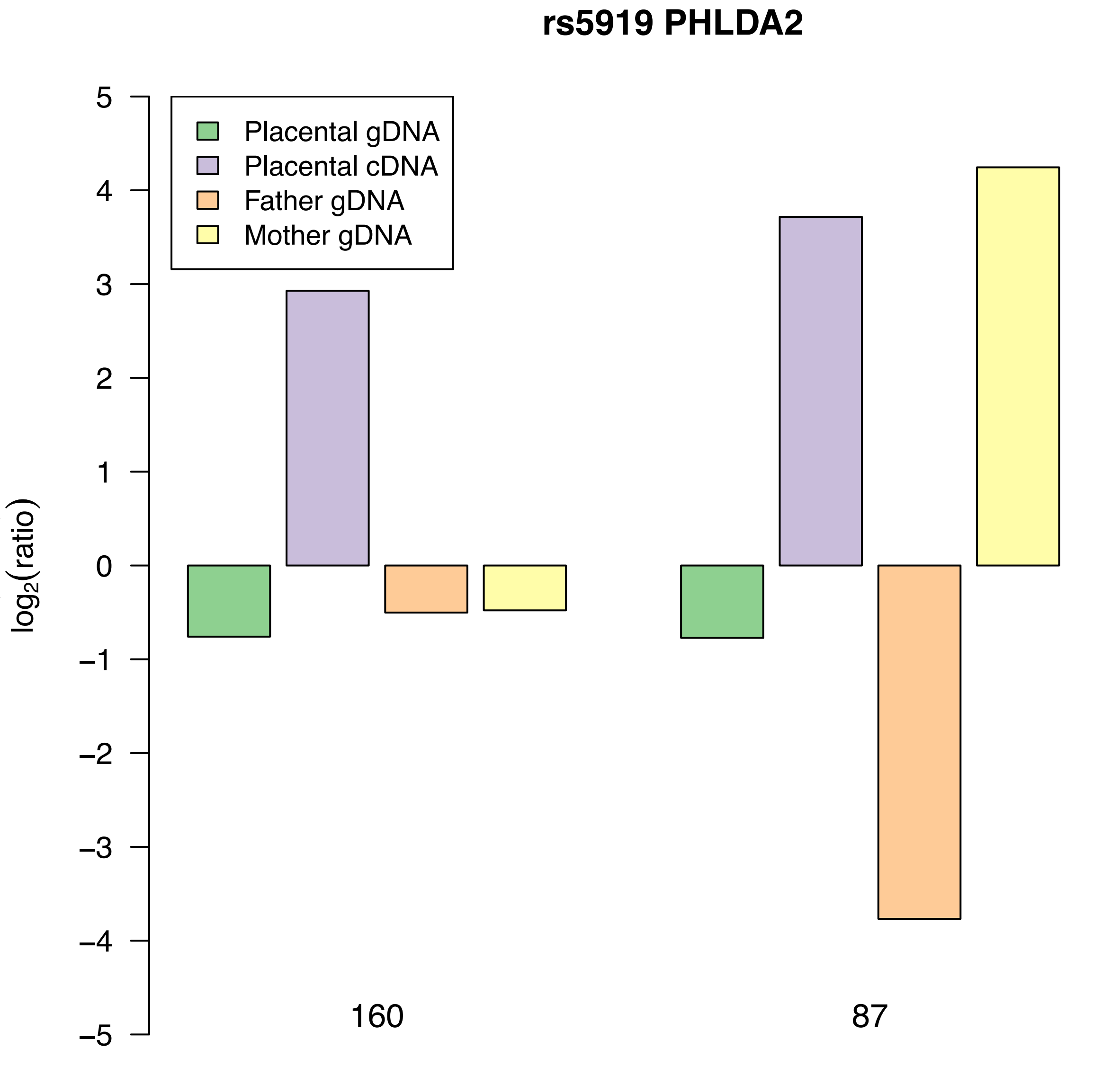
rs5919 PHLDA2
Placental gDNA
Placental cDNA
Father gDNA
Mother gDNA
$\log_2(\text{ratio})$
5
4
3
2
1
0
−1
−2
−3
−4
−5
160
87

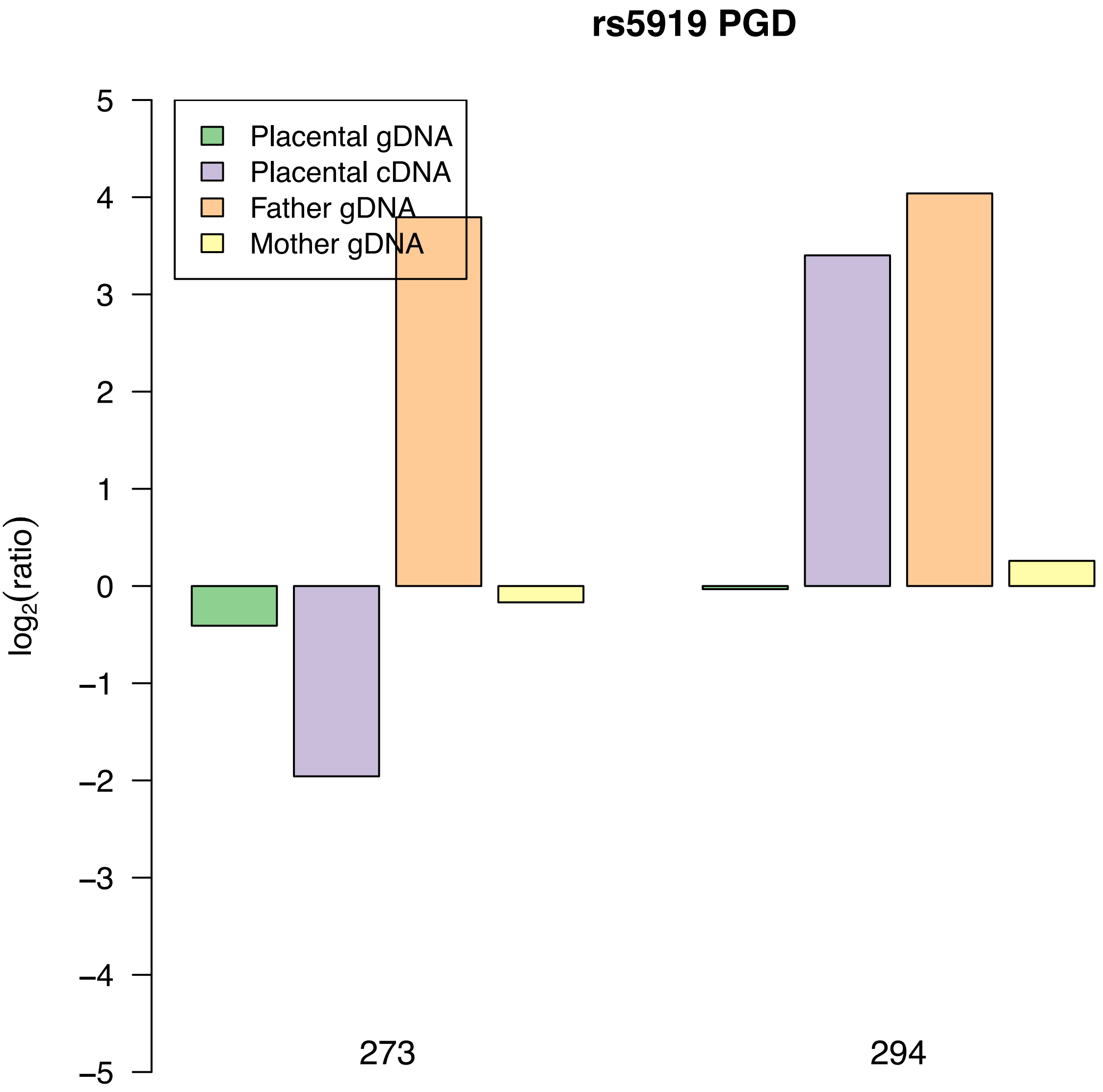
rs5919 PGD
Placental gDNA
Placental cDNA
Father gDNA
Mother gDNA
5
4
3
2
1
0
−1
−2
−3
−4
−5
log2(ratio)
273
294

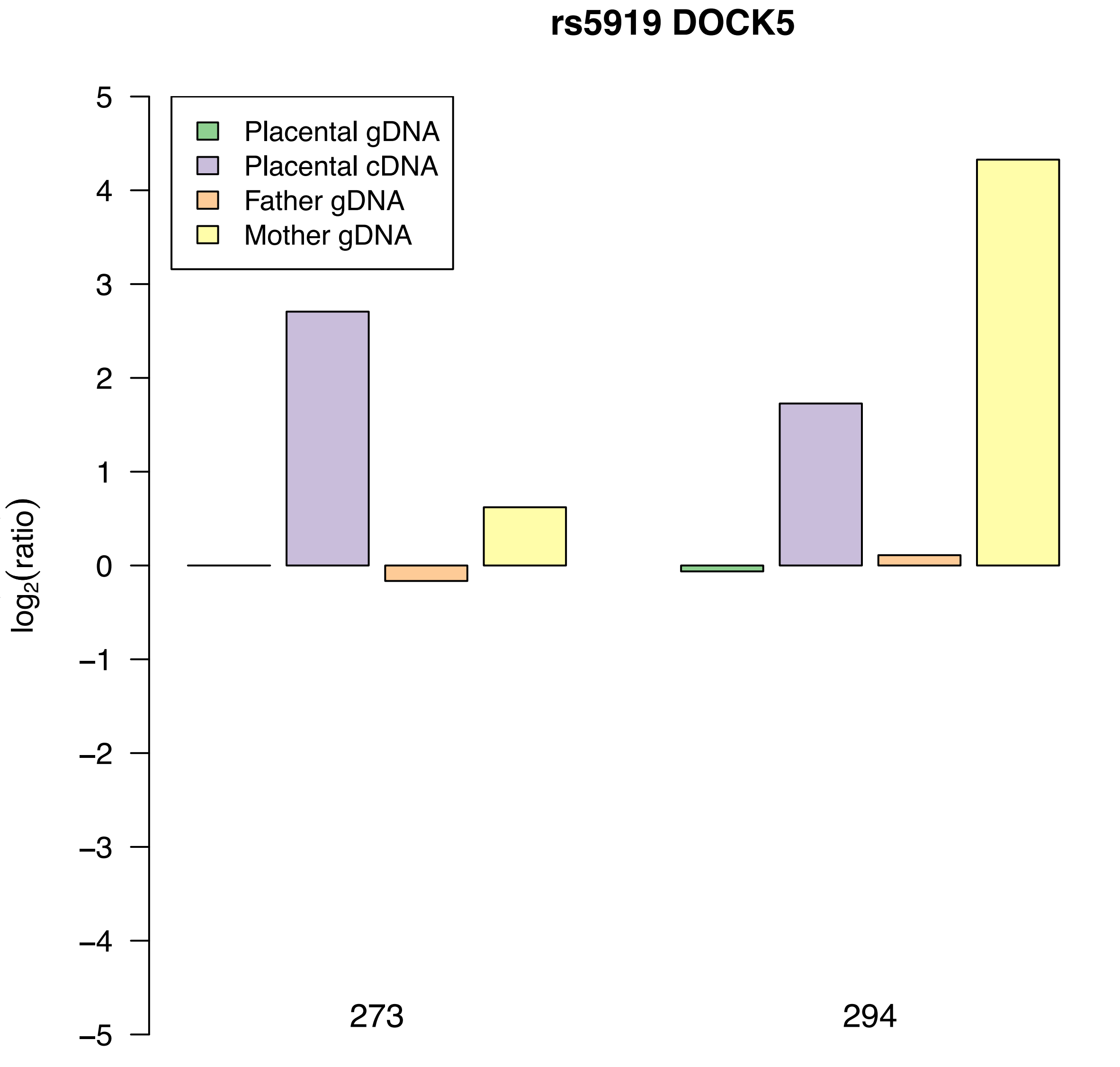

rs5919 DOCK5
Placental gDNA
Placental cDNA
Father gDNA
Mother gDNA
log2(ratio)
5
4
3
2
1
0
−1
−2
−3
−4
−5
273
294

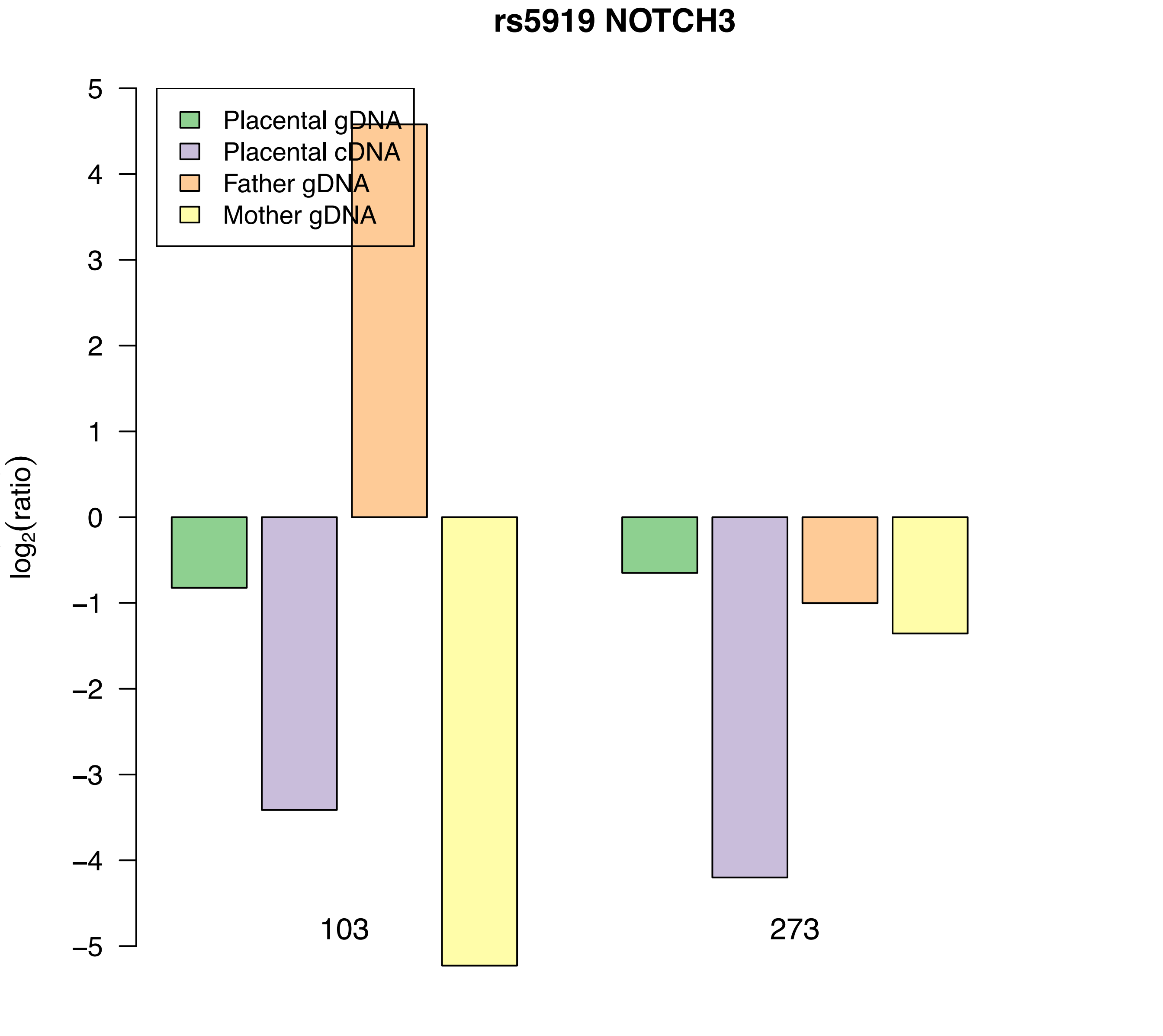
rs5919 NOTCH3
Placental gDNA
Placental cDNA
Father gDNA
Mother gDNA
log2(ratio)
5
4
3
2
1
0
−1
−2
−3
−4
−5
103
273

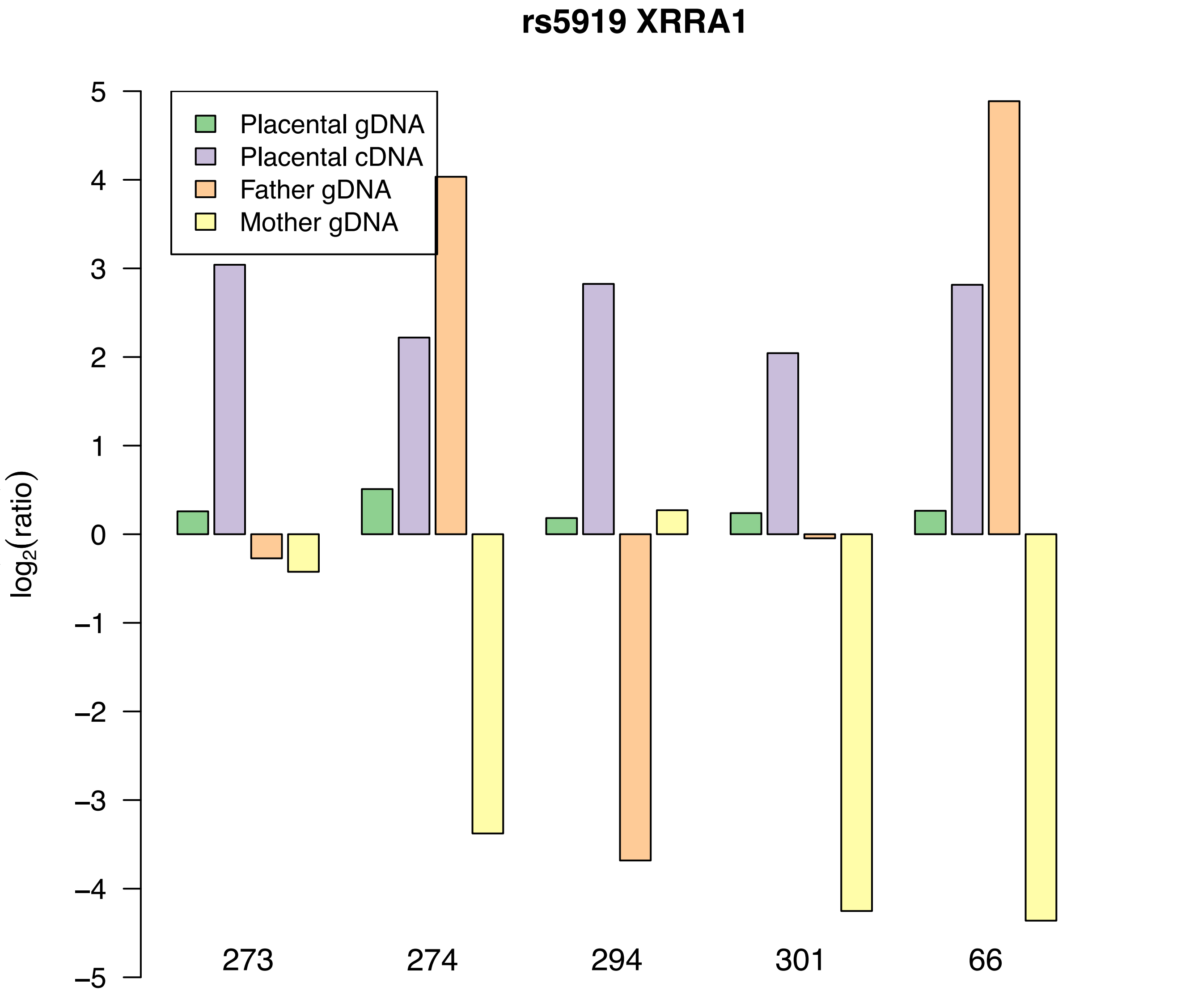

rs5919 XRRA1
Placental gDNA
Placental cDNA
Father gDNA
Mother gDNA
log2(ratio)
5
4
3
2
1
0
−1
−2
−3
−4
−5
273
274
294
301
66

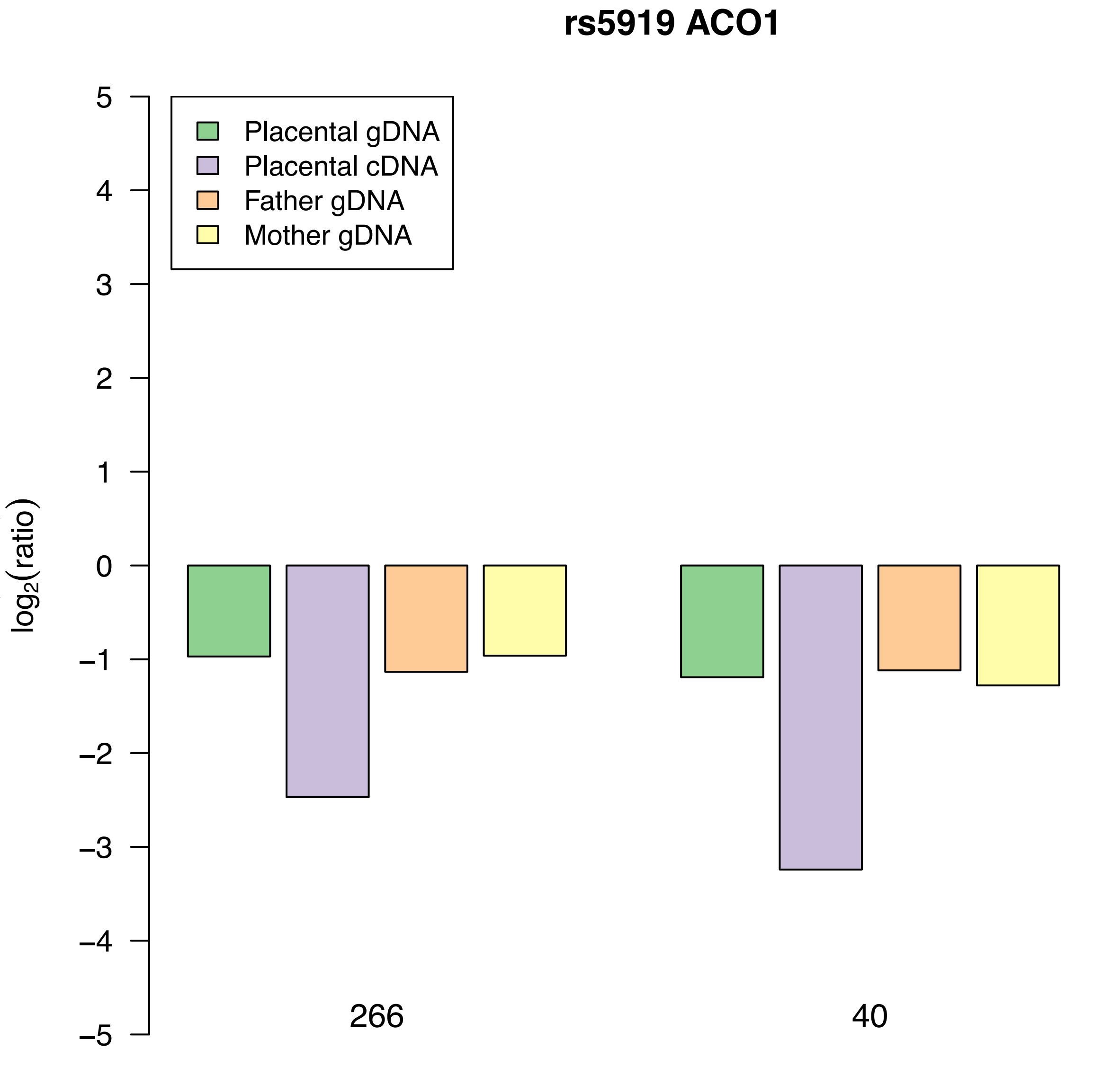
rs5919 ACO1
Placental gDNA
Placental cDNA
Father gDNA
Mother gDNA
log2(ratio)
5
4
3
2
1
0
−1
−2
−3
−4
−5
266
40

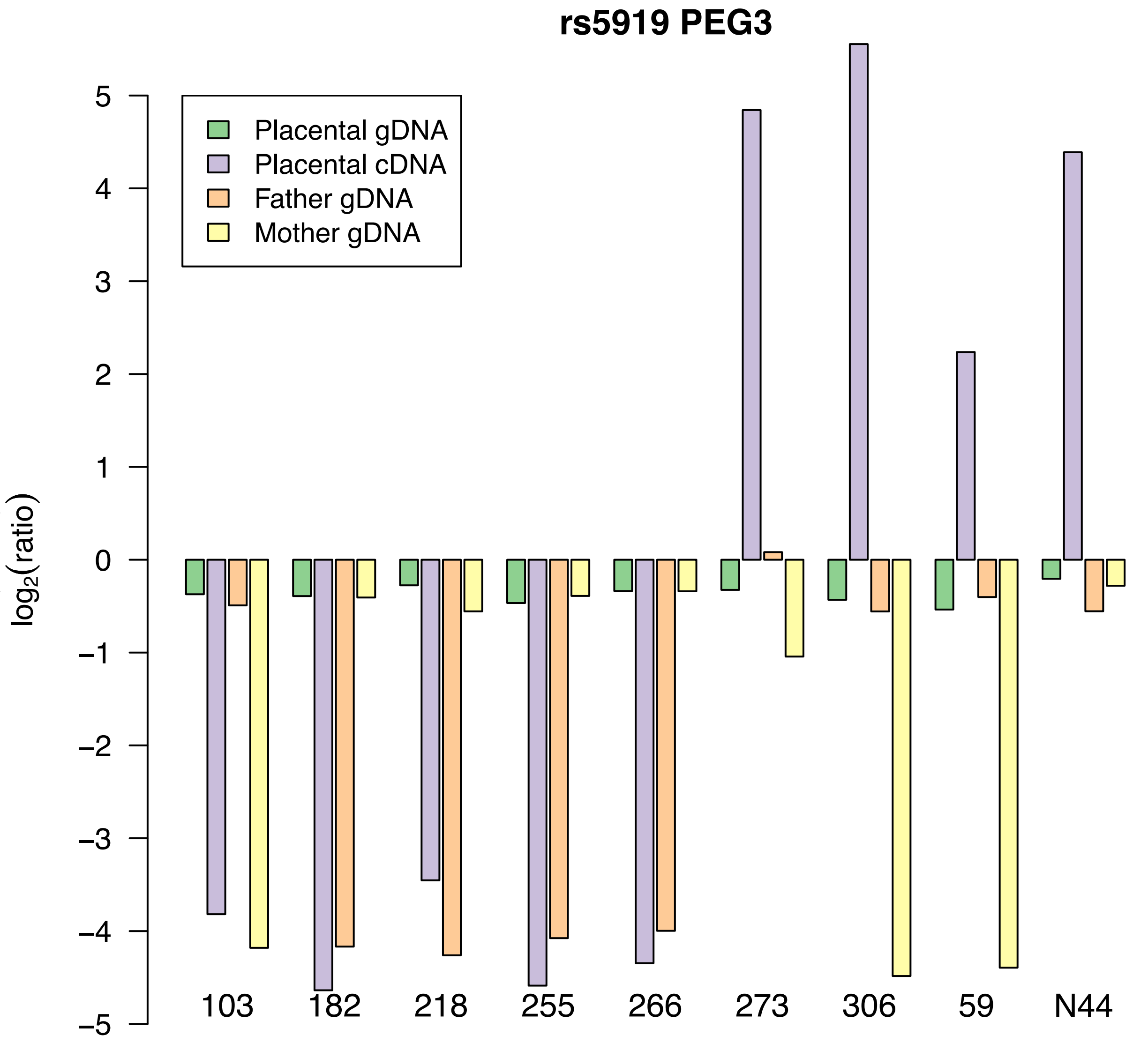
rs5919 PEG3
Placental gDNA
Placental cDNA
Father gDNA
Mother gDNA
log2(ratio)
5
4
3
2
1
0
−1
−2
−3
−4
−5
103
182
218
255
266
273
306
59
N44

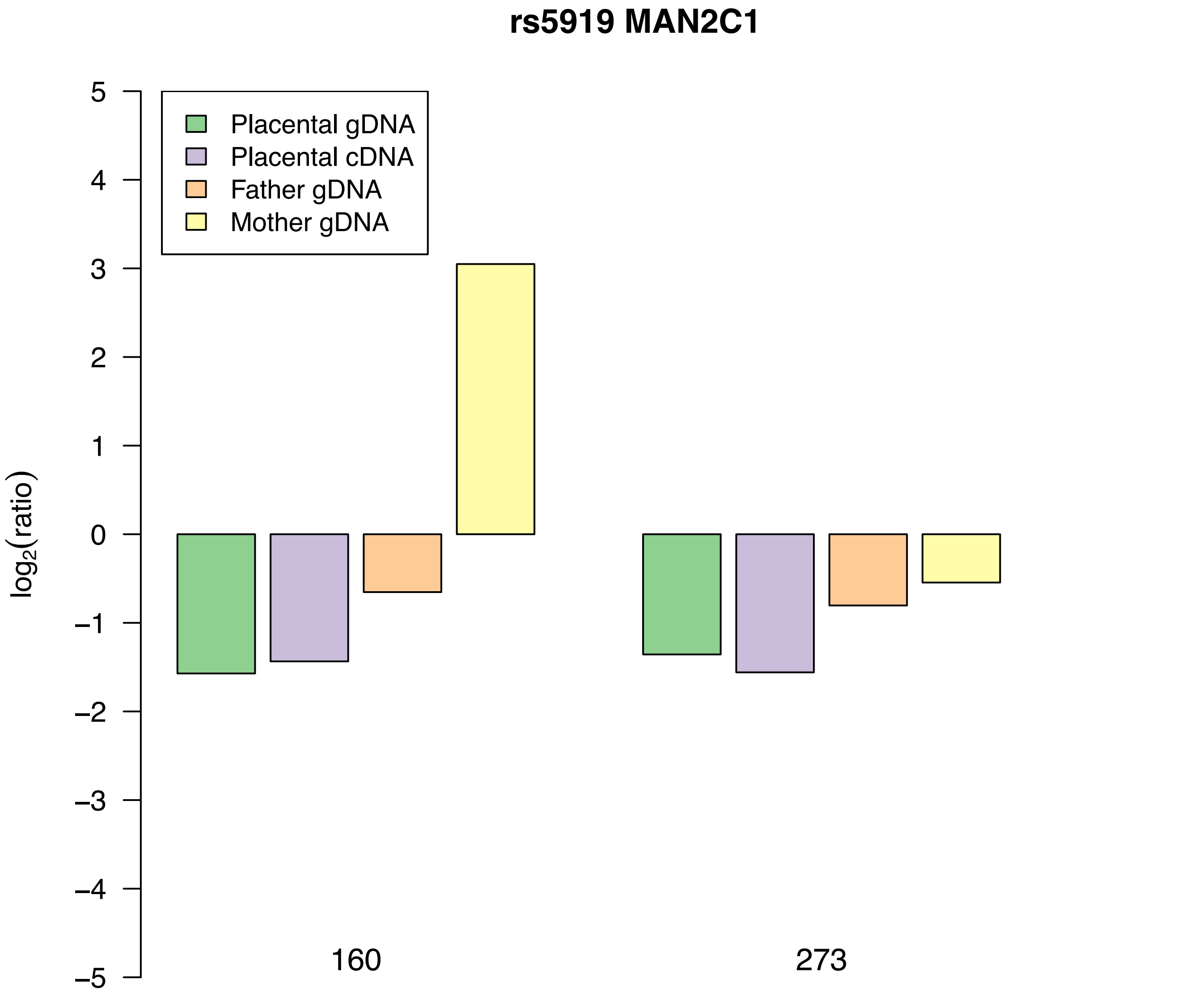
rs5919 MAN2C1
Placental gDNA
Placental cDNA
Father gDNA
Mother gDNA
log2(ratio)
5
4
3
2
1
0
−1
−2
−3
−4
−5
160
273
