# Supplementary material for: High-throughput analysis of candidate imprinted genes and allele-specific gene expression in the human term placenta
Source: BMC Genet. 2010 Apr 19;11:25. doi: 10.1186/1471-2156-11-25 (PMC2871261; doi:10.1186/1471-2156-11-25)
Supplement: Additional file 4 — Figure showing statistically significant genes exhibiting preferential ASE on the Illumina array. ASE for SQSTM1, UBE2V1 and XRRA1 is evident while the effect for CAST and MAN2C1 is more subtle. [file 1471-2156-11-25-S4.PPT]

## Slide 1
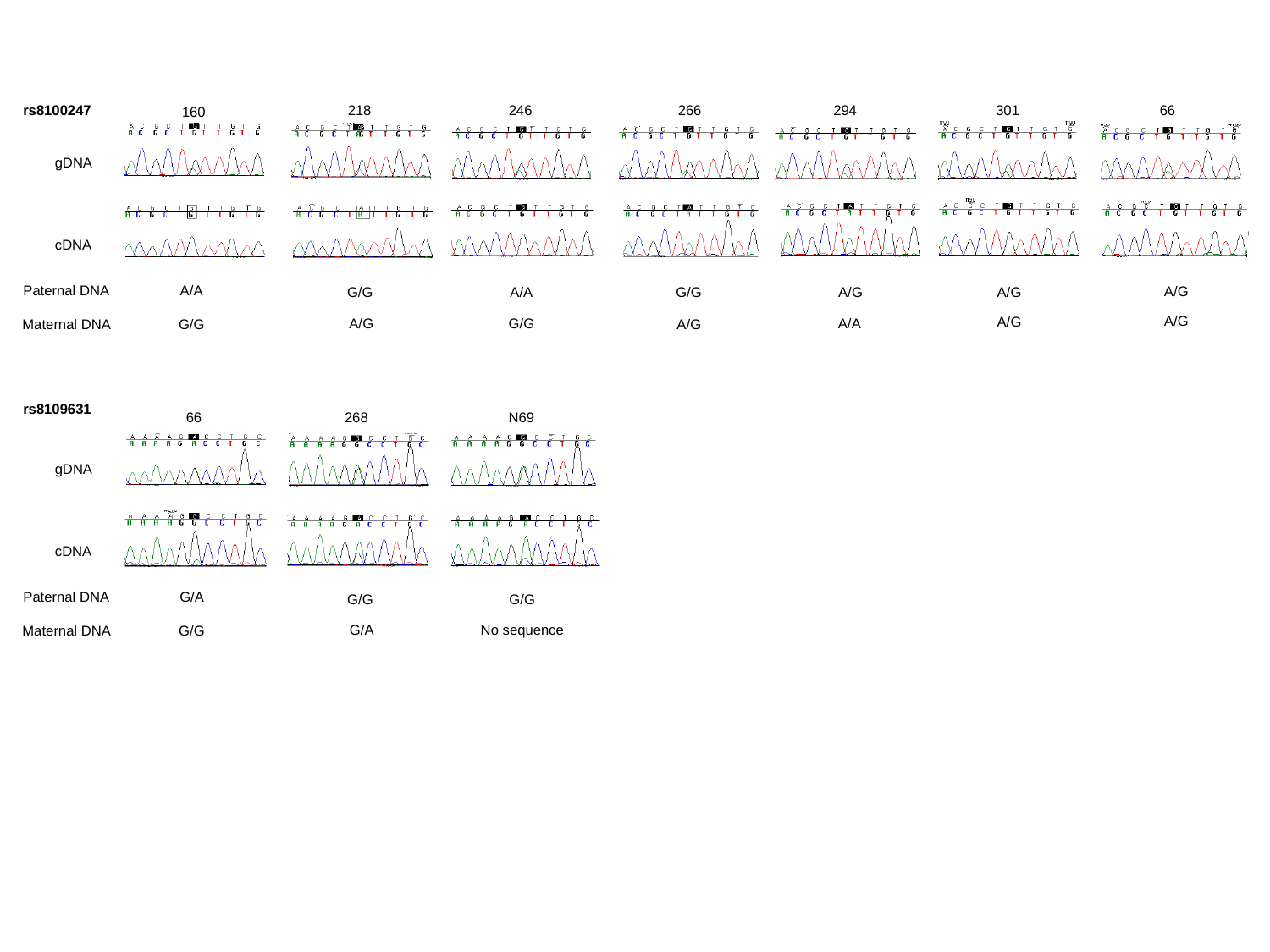

rs8100247
218
246
266
294
301
66
160
gDNA
cDNA
Paternal DNA
A/A
A/G
G/G
A/A
G/G
A/G
A/G
A/G
A/G
A/G
G/G
A/A
Maternal DNA
G/G
A/G
rs8109631
66
268
N69
gDNA
cDNA
Paternal DNA
G/A
G/G
G/G
G/A
No sequence
Maternal DNA
G/G
